# Evaluating strategies to improve HIV care outcomes in Kenya: a modelling study

**DOI:** 10.1016/S2352-3018(16)30120-5

**Published:** 2016-10-19

**Authors:** Jack J Olney, Paula Braitstein, Jeffrey W Eaton, Edwin Sang, Monicah Nyambura, Sylvester Kimaiyo, Ellen McRobie, Joseph W Hogan, Timothy B Hallett

**Affiliations:** a Department of Infectious Disease Epidemiology, Imperial College London, London, UK; b Dalla Lana School of Public Health, University of Toronto, Toronto, ON, Canada; c Moi University, College of Health Sciences, School of Medicine, Department of Medicine, Eldoret, Kenya; d Academic Model Providing Access to Healthcare, Eldoret, Kenya; e Department of Biostatistics and Center for Statistical Sciences, Brown University School of Public Health, Providence, RI, USA

## Abstract

**Background:**

With expanded access to antiretroviral therapy (ART) in sub-Saharan Africa, HIV mortality has decreased, yet life-years are still lost to AIDS. Strengthening of treatment programmes is a priority. We examined the state of an HIV care programme in Kenya and assessed interventions to improve the impact of ART programmes on population health.

**Methods:**

We created an individual-based mathematical model to describe the HIV epidemic and the experiences of care among adults infected with HIV in Kenya. We calibrated the model to a longitudinal dataset from the Academic Model Providing Access To Healthcare (known as AMPATH) programme describing the routes into care, losses from care, and clinical outcomes. We simulated the cost and effect of interventions at different stages of HIV care, including improvements to diagnosis, linkage to care, retention and adherence of ART, immediate ART eligibility, and a universal test-and-treat strategy.

**Findings:**

We estimate that, of people dying from AIDS between 2010 and 2030, most will have initiated treatment (61%), but many will never have been diagnosed (25%) or will have been diagnosed but never started ART (14%). Many interventions targeting a single stage of the health-care cascade were likely to be cost-effective, but any individual intervention averted only a small percentage of deaths because the effect is attenuated by other weaknesses in care. However, a combination of five interventions (including improved linkage, point-of-care CD4 testing, voluntary counselling and testing with point-of-care CD4, and outreach to improve retention in pre-ART care and on-ART) would have a much larger impact, averting 1·10 million disability-adjusted life-years (DALYs) and 25% of expected new infections and would probably be cost-effective (US$571 per DALY averted). This strategy would improve health more efficiently than a universal test-and-treat intervention if there were no accompanying improvements to care ($1760 per DALY averted).

**Interpretation:**

When resources are limited, combinations of interventions to improve care should be prioritised over high-cost strategies such as universal test-and-treat strategy, especially if this is not accompanied by improvements to the care cascade. International guidance on ART should reflect alternative routes to programme strengthening and encourage country programmes to evaluate the costs and population-health impact in addition to the clinical benefits of immediate initiation.

**Funding:**

Bill & Melinda Gates Foundation, United States Agency for International Development, National Institutes of Health.

## Introduction

The provision of antiretroviral therapy (ART) has substantially reduced HIV mortality.^1^ With timely diagnosis, treatment initiation, and good adherence, life-expectancy for people with HIV can approach that of uninfected people.^2^ However, life-years are still lost to AIDS, and in sub-Saharan Africa, hundreds of thousands of AIDS-related deaths occur each year.^3^

Reasons for continued health losses to HIV when ART is widely available are poorly understood. The care cascade describes the series of engagements with the health system through which people with HIV must pass to benefit fully from ART, beginning with HIV testing, and ending with regular monitoring of patients in a state of sustained viral suppression. In 2011, Rosen and Fox^4^ showed that fewer patients reach each successive stage of HIV care in sub-Saharan Africa, and representations of the care cascade in different settings followed.^5^ However, many attempts to quantify the care cascade have been limited by not being able to follow a cohort of patients through all stages of care. Data are typically available only for people with HIV who present to clinics and therefore exclude those who never engage in care and who are likely to have the greatest health losses.^6^, ^7^ Differences in care-seeking behaviour between patients who actively seek care (through clinics) and those who are actively sought (through outreach programmes) lead to fundamental uncertainties in the operation of the care cascade, in particular, the extent to which health-care-seeking behaviour enables patients to present for care and initiate treatment on becoming ill, bypassing the typical stages of pre-ART care, and monitoring until reaching ART eligibility (in a process that we have termed previously as reaching ART via the side-door).^6^

**Research in context**

**Evidence before this study**

With global aspirations to eliminate AIDS as a cause of population morbidity and death, attention has turned to identification and reduction of weaknesses in HIV care programmes. As the breadth of interventions targeting aspects of the cascade of care expands, care programmes must identify which strategies will bring about the largest impact for the lowest cost. Mathematical models are well placed to answer these questions. We searched PubMed for HIV modelling studies published between Jan 1, 2000, and Aug 2, 2016, with the terms “HIV” AND (“ART” OR “antiretroviral therapy”) AND (“cascade” OR “continuum”) AND (“modelling” OR “modeling” OR “model”) without any language restrictions. The search yielded 65 abstracts and 12 met our inclusion criteria of being mathematical modelling studies. Previous studies have relied on aggregated routine data from disparate analyses and populations to estimate the cascade, and consequently most have used deterministic compartmental models to do investigations on individual interventions. Only four studies have assessed the cost-effectiveness of interventions targeting improvements in care. Among these, analysis of the effect of early ART initiation in a study in India showed such an intervention would be highly cost-effective, but the researchers stressed the importance of the modulatory effects of retention in care. Findings from analyses of the cascade in the USA corroborate these results. The only study from our search to use an individual-based model described HIV transmission and cascade progression in South Africa. Results indicated that among the interventions implemented, returning and reinitiating patients onto ART is highly cost-effective along with improving retention in care.

**Added value of this study**

Our model shows that single interventions have a modest effect on improving present care programmes. A combination of interventions concurrently strengthening various aspects of care is the most cost-effective strategy to improve outcomes for patients. This strategy is likely to be more cost-effective and generate greater impacts than immediate ART that is not accompanied by improvements to the care cascade. Our model represents HIV care and treatment in western Kenya, but our overall conclusions will have the same broad relevance to the many other settings with large generalised epidemics and established ART programmes.

**Implications of all the available evidence**

Many health systems do not capture a large proportion of HIV-related deaths occurring outside of the clinic. There is considerable scope for care programmes to improve care throughout the cascade. Detailed longitudinal data about ART health-care programmes could improve future projections, characterise country-specific gaps in care, and identify cost-effective strategies to achieve future treatment targets.

Many strategies to improve the care cascade have been proposed and tested.^8^, ^9^, ^10^ These variously aim to improve testing, linkage to care, retention in care before starting treatment, retention on ART, and rates of viral suppression. However, evidence about the effectiveness of individual interventions is only partly informative about the best strategy to improve HIV care, and most studies have not been able to measure the eventual population-health benefits resulting from improved provision of care.

The expansion of ART eligibility to potentially all people with HIV as recommended in the latest WHO guidelines (ie, immediate ART initiation)^11^ and increased outreach to populations for testing (eg, through a universal test-and-treat strategy) are proposals for transformational expansions to treatment.^12^, ^13^ In hypothetical idealised programmes, the persuasive argument in favour of such approaches is that the additional initial costs would later become offset by savings resulting from reductions in HIV transmission and need for ART.^12^ However, whether these savings will be realised with more realistic implementation assumptions is questionable.^14^

Definitions for optimal strategies to improve HIV care most efficiently in health systems require insight into the sources of HIV mortality and morbidity for patients at each stage of care and a comparison of the population-level health impact of a range of candidate interventions that act at different stages of care. We created a mathematical model of the HIV epidemic and care cascade in Kenya. The model is parameterised with data from an HIV care programme in western Kenya supported by the Academic Model Providing Access To Healthcare (AMPATH) collaboration, which uniquely includes data about people before testing and disengagement from care, through an integrated household-based testing intervention. We used the model to quantify the previous care experience of those dying from HIV in a setting with a mature (>8 years since established) ART programme, and to simulate the cost and effect of HIV care interventions, in isolation and in combinations. From this information, we aimed to generate recommendations about optimal strategies for HIV care programmes to maximise health costs effectively.

## Methods

### Data sources

We constructed an individual-based microsimulation model representing the HIV epidemic in Kenya and capturing the care experience of individuals as they progress through an ART programme.

The model represents births, ageing, deaths, and HIV transmission in the Kenyan population (appendix pp 5–8). After infection, disease progression is modelled by an individual progressing to a lower CD4 count category (>500 cells per μL, 350–500 cells per μL, 200–350 cells per μL, and <200 cells per μL) and of greater disease severity (WHO defined stages 1, 2, 3, and 4; appendix p 12). Both CD4 cell count and disease state affect the risk of mortality. Additionally, WHO disease stage affects the propensity to seek care (appendix pp 12–18). When treatment is initiated, patients can transition to a higher CD4 cell count and a lower (healthier) WHO clinical stage (appendix p 12).

### Model design

**Figure 1:**
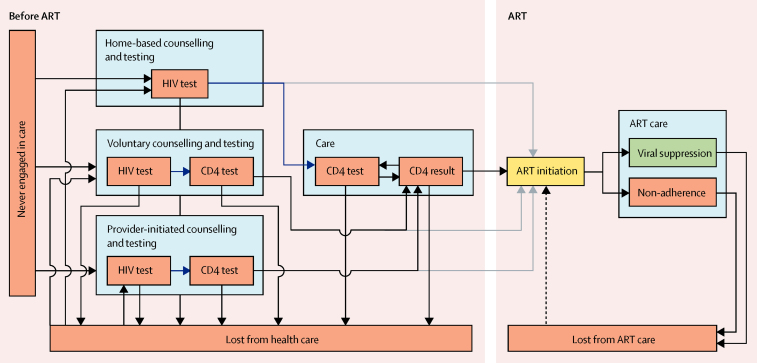
Operational steps involved in navigating an ART programme Blue arrows show linkage step in which patients were seen by a clinician and had blood taken for a CD4 test. Grey arrows show the shortcut to immediate ART initiation taken by individuals presenting with WHO stage 3 or 4 symptoms. Dashed arrow shows ART reinitiation after loss from ART care (does not occur in the baseline programme). ART=antiretroviral therapy.

The model describes the pathway through care for each individual infected with HIV (figure 1, appendix p 19). The baseline scenario represents no additional intervention.

The HIV care and treatment components of the model were parameterised with observed patients' data from Bunyala, western Kenya (appendix pp 19–44). HIV testing, HIV care, and treatment services were established in 2006 in district hospital and health centres, supported by the AMPATH collaboration. AMPATH is a partnership established in 2001 between Moi Teaching and Referral Hospital, Moi University School of Medicine, and a consortium of universities from North America in response to the HIV epidemic in Kenya (appendix pp 19–20). All patients' visits from 2004 have been recorded electronically in the AMPATH Medical Record System, furnishing information about retention and outcomes. For patients to seek care in non-AMPATH clinics they must leave the AMPATH catchment area (appendix pp 19–20). Patients lost from care are traced and actively followed up to ascertain their outcome, either through direct contact or discussions with family members if patients cannot be found. In the area of Bunyala, there have been two rounds of home-based counselling and testing campaigns since 2010, the first of which achieved more than 85% coverage of the community.^15^ These early rounds of home-based counselling and testing involved passive referral of patients infected with HIV, with no active follow-up to facilitate linkage to health care (appendix p 24). Present AMPATH home-based counselling and testing campaigns now include active follow-up, and we expect linkage rates to be substantially higher.

We characterised the main parameters of the care cascade through the analysis of the linked individual clinical and home-based counselling and testing records after extensive removal of incorrectly entered data and removal of duplication (appendix p 21).^15^ For model parameters that did not correspond to quantities that can be directly observed, values were inferred through fitting the model output to observed data (table 1, appendix pp 22, 23, 25–28). 62% of people in both AMPATH and model data were diagnosed in mid-2010: 41% through voluntary testing and counselling in AMPATH and 42% in the model; 21% provide initiated counselling and testing in each. Of those diagnosed, AMPATH data showed 34% were on ART in 2010 and the model estimated 33%; by 2014 an estimated 91% (AMPATH) and 82% (model) of diagnosed people were on ART.

Calibration yielded several sets of parameters for the model of the care cascade, which are variously in better agreement with different indicators (appendix pp 25–44). The ART programme costs at baseline were estimated from the perspective of a health-care provider. Unit cost estimates were based on the CHAI MATCH study^17^ of ART facilities, and comprised the cost of ART care, the cost of pre-ART clinic visits, and the cost of CD4 laboratory-based tests (appendix pp 45, 46). Sensitivity analysis was done with consideration to the variations in the unit cost of ART programme components (appendix pp 53–55).

### Analysis of the care cascade

Interventions on the care cascade can be divided into those that aim to increase testing, linkage, and retention in pre-ART health care, or retention and suppression for patients on ART. We reviewed the medical literature to identify realistic and empirically based assumptions for the efficacy and cost of representative interventions in each of these categories (table 2, appendix p 48).

To assess the impact of individual interventions, each was simulated for the duration 2010–30, and the effect on patients' outcomes compared with the baseline scenario in the absence of any interventions. The effect of the programme was quantified as disability-adjusted life-years (DALYs) averted (appendix p 47), additional cost of care (appendix pp 45, 46), and HIV-related deaths averted, compared with a baseline programme without any intervention (taken to be similar in structure to AMPATH before the launch of household-based testing). Because of the stochastic nature of the model, we present results as the mean of ten repeat simulations.

An optimal combination of individual cascade interventions (excluding universal test and treat, which is a composite of home-based counselling and testing and immediate ART) was identified by simulation of all possible combinations and selection of those that provided the greatest increase in health for a range of budget levels. We imposed the additional constraint that once an intervention had been included in the combination at one budget level, it cannot be removed at higher budget levels.

To assess the cost-effectiveness of interventions, the cost per DALY averted was compared with the gross domestic product (GDP) per capita for Kenya in 2013 (US$1242).^24^ We assume that an intervention is likely to be cost-effective if the cost per DALY is less than 50% GDP per capita.^25^, ^26^ Costs and DALYs were both discounted at 6% per annum from 2010.

### Role of the funding source

The funders had no role in model construction, data collection, data analysis, data interpretation, or writing of the report. The corresponding author worked with co-authors to analyse the data in the study and had final responsibility for the decision to submit for publication.

## Results

**Figure 2:**
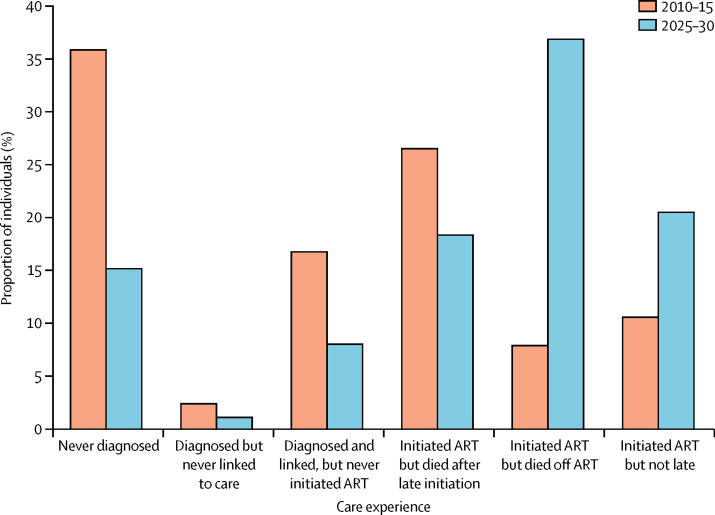
Distribution of care experience of patients who died from HIV ART=antiretroviral therapy. Late initiation is defined as a person with a CD4 count of less than 200 cells per μL.

We projected the baseline model in the absence of any interventions between 2010 and 2030 and analysed the status of care of those dying from HIV-related causes in two timeframes: 2010–2015 and 2025–2030. Between 2010 and 2030, most people will have initiated treatment (61%), but many will never have been diagnosed (25%) or will have been diagnosed but never started ART (14%). Among all AIDS-related deaths between 2010 and 2015, most occurred in individuals who had initiated treatment (figure 2). Among these, most died because they had initiated treatment late (CD4 count <200 cells per μL). The largest proportion of deaths was in people who were never diagnosed, with the remainder those who were diagnosed but did not initiate ART. By contrast, between 2025 and 2030, the distribution of mortality shifted to the latter stages of care. Most deaths still occurred in individuals who had initiated treatment, with the largest single proportion being in patients who had disengaged from ART care.

**Figure 3:**
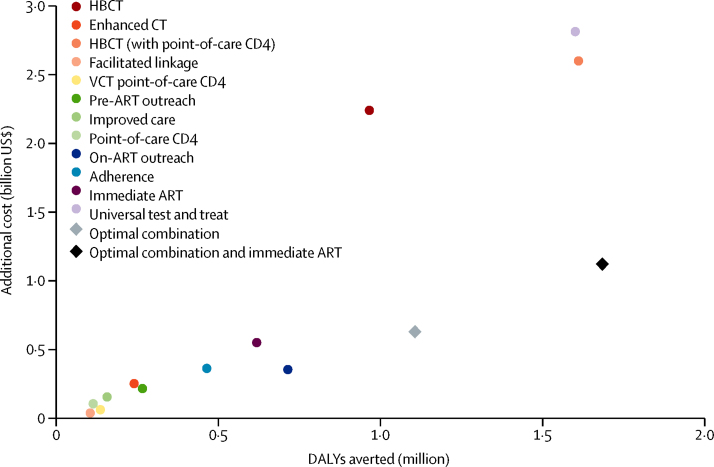
Disability-adjusted life-years averted and additional cost of care (based on 2013 US$) for interventions acting on the cascade between 2010 and 2030 Optimal combination of interventions includes facilitated linkage, on-ART outreach, VCT point-of-care CD4, pre-ART outreach, and point-of-care CD4. ART=antiretroviral therapy. HBCT=home-based counselling and testing. VCT=voluntary counselling and testing.

We applied each of the 12 interventions in isolation and calculated the DALYs averted and additional costs between 2010 and 2030 compared with the baseline scenario (figure 3 and table 3). Costs and effect are generally closely related, with low-cost interventions having a low impact. The effects of most single interventions affecting engagement in pre-ART health care cluster together with relatively low impact and low cost (figure 3).

One exception is home-based counselling and testing (with passive referral), which has a high cost per DALY averted (figure 3). We assumed that only 30% of people diagnosed for the first time at home-based counselling and testing will link to care without further intervention. This assumption is based on observations in the earliest home-based counselling and testing campaign at AMPATH, which used passive referral to care.^15^ AMPATH's present home-based counselling and testing programme incorporates active referral and, based on preliminary data, yields considerably higher linkage rates (a scenario with 90% linkage, consistent with WHO targets, is included for comparison in the appendix p 56). Home-based counselling and testing (with point-of-care CD4) averts more DALYs than any other single intervention because of the reduced time to confirm eligibility and increased probability of linkage. However, the cost per DALY averted of $1617 is 130% GDP per capita of Kenya (table 3).

By contrast, the on-ART outreach intervention, which seeks and returns 40% of people disengaged from health care after ART initiation, has the third lowest cost per DALY averted, and a larger impact than almost all interventions (table 3). These data are consistent with the large proportion of deaths and high mortality in people disengaged from care (figure 2).

The two interventions that represent large changes to ART eligibility, immediate ART and universal test and treat, differ substantially in their impact and cost. Immediate ART provides treatment to those who present for care, and is effective because it eliminates the potential for patients to be lost from care before they are confirmed to be eligible for ART. The cost per DALY averted is $895 (72% GDP per capita), which is not considered to be cost-effective at the threshold of 50% GDP per capita (which is becoming widely used), but would be considered cost-effective with previous thresholds.^27^

The universal test-and-treat intervention has a much larger impact than any other intervention because the home-based testing results in a higher population ART coverage, but is much more costly because of high HIV testing costs. This approach is also not cost-effective ($1760 per DALY averted; 142% GDP per capita).

Most of the projected benefits of single interventions are because of the direct therapeutic effect of ART averting morbidity and mortality, rather than the secondary effects of fewer HIV transmissions (appendix p 51). Furthermore, with adjusted calibrations, if health-care seeking behaviour is greater and is not strongly related to symptoms, pretreatment interventions generate less impact as patients enter the health-care system earlier without any additional intervention (appendix p 49). By contrast, if care is only sought when a patient is symptomatic, the outreach intervention before ART initiation has twice the impact, as patients do not return to care faster when lost (appendix p 50).

Finally, sensitivity analysis varying the unit costs of different aspects of care showed that the absolute cost of interventions is most sensitive to the cost of ART, but the rank order of cost per DALY averted for interventions is preserved when unit costs are varied over reasonable (±50%) ranges (appendix pp 53–55).

We identified a combination of five interventions that maximise the health gained from a budget of $700 million (table 4). This budget was chosen because it is about equal to the cost of implementing immediate ART, but is less than the cost of implementing home-based counselling and testing in isolation. The five selected interventions, in the order in which they were added with increasing budget, were: pre-ART outreach, facilitated linkage, voluntary counselling and testing point-of-care CD4, point-of-care CD4, and on-ART outreach. The next intervention to be added would be enhanced counselling and testing, comprising a set of interventions that act on each part of the cascade at a total budget 67% lower than that of the home-based counselling and testing intervention in isolation (table 4).

Collectively, this combination of interventions reduces AIDS deaths by 19% relative to baseline, the same reduction as the universal test-and-treat intervention, and averts 69% as many DALYs. It averts 77% more DALYs than the immediate ART intervention. However, the combination of cascade interventions costed 22% as much as universal test and treat and only 14% more than immediate ART. This combination approach is estimated to be cost-effective, at a cost of $571 per DALY averted (46% GDP per capita; figure 3).

The comparatively low cost and high impact of the combination cascade intervention with this budget is the result of a collection of interventions operating synergistically, whereas the universal test and treat and immediate ART interventions incur inefficiencies because of the remaining weaknesses in the cascade. These synergies among interventions are exemplified by the point-of-care CD4 and pre-ART outreach interventions, for which the incremental cost-effectiveness ratio of both interventions together is lower than for either intervention alone (table 4). A combination of both strengthening of the cascade and changing eligibility to immediate ART averted 31% of AIDS-related deaths relative to baseline, at a cost of 54% GDP per capita (table 4).

## Discussion

Our results suggest that ART programmes can be substantially and cost-effectively improved by strengthening each part of the care cascade through a combination of interventions. By contrast, simply moving to immediate ART would have less impact for the same cost as a combination of interventions. Although a universal test-and-treat strategy would generate greater health benefits than a combination of interventions, it would not be cost-effective if the weaknesses in the cascade were not resolved.

In the coming years, one of the most cost-efficient interventions would be to find people who have disengaged from ART care (on-ART outreach). However, we reported that no individual pre-ART intervention has had a large effect on patient outcomes except annual testing interventions with substantial costs (figure 3). This finding is a result of the multifaceted nature of weakness in the present pre-ART care cascade. We do find that combinations of interventions at all parts of the cascade can have a large effect and be cost-effective. The reason that interventions affecting a single care stage have a modest effect is because there are weaknesses throughout the care cascade, so any potential impact is attenuated by remaining weaknesses elsewhere.

Published trials have tended to examine the effect of single interventions on the cascade,^8^ because measurements of the effect of a combination of changes simultaneously poses challenges for robust experimental designs. Our model allows the effect of interventions, applied individually or in combination, to be assessed across the entire cascade. However, implementation studies investigating the effect and feasibility of complete combinations of interventions are needed to validate our findings. If greater synergy among interventions can be achieved than our model simulations, greater benefits might be realised at lower costs. Alternatively, the increased complexity of operations could lead to higher costs and fewer benefits. Therefore, trials of each individual and combination of interventions are needed to further confirm our results in western Kenya.

Attention has focused on the marginal therapeutic benefits to a patient who has had early initiation of ART. A potentially large benefit of earlier ART initiation is to reduce the risk of losing a patient from the pretreatment monitoring phase, so lessening the chance of further transmission and the risk of death from AIDS before such a time as the patient might return to care. Furthermore, earlier initiation in the form of immediate ART would continue to prioritise treatment for patients with low CD4 counts as per WHO guidelines.^11^ Our model did not consider the potential effect of interventions that reposition ART initiation (eg, home initiation), which have been shown in recent trials.^28^

Neither of the strategies (combinations of cascade interventions or immediate ART for those presenting) would reduce the risk of deaths from AIDS among those not already diagnosed (appendix p 52). This issue would potentially exacerbate disparities in overall health between those who are better able to seek care. The effect of immediate ART is enhanced by further strengthening of care through a combination of interventions (figure 3). This result would lead to reductions in mortality before and after starting ART by outreach strategies returning more patients to treatment than in the absence of immediate ART.

Our model suggests that a large proportion of HIV-related deaths occur in individuals never diagnosed and those who were diagnosed but never initiated treatment. These results are in agreement with data from general population cohort studies in Rakai (Uganda) and uMkhanyakude (South Africa).^29^ Many health data systems account only for individuals who have registered with the clinic, and therefore will not capture this source of AIDS mortality (figure 2). Monitoring and evaluation frameworks for the cascade should therefore seek to quantify the extent to which deaths from AIDS are among those undiagnosed and treatment guidelines should recognise testing as an integral part of treatment programmes.

Therefore, to achieve the vision of eliminating deaths from AIDS, substantial active outreach is required to identify all individuals infected with HIV before needing ART. This goal is likely to incur substantial costs, but the exact cost is unknown and in well documented existing care programmes, costs vary between studies.^17^ For instance, an independent modelling analysis of a home-based counselling and testing intervention with active follow-up of patients piloted in Kwazulu-Natal (South Africa) reported that the intervention was cost effective,^9^ whereas home-based counselling and testing with passive referral was not cost-effective in our analysis. This discrepancy is likely because the KwaZulu-Natal study with active follow-up achieved a much higher rate of linkage to care (90%) than the 30% we assumed for our simulated home-based counselling and testing intervention with passive referral. Ongoing analysis of AMPATH home-based counselling and testing rounds will provide an opportunity to examine both efficacy and cost-effectiveness of this intervention with active referral in a different setting, which might show important ways in which its efficacy can be maximised.

Comparison of our alternative model parameterisations (appendix pp 49, 50) shows how patterns of health-care seeking behaviour can modify the effect of interventions on population health, which has not been readily apparent from empirical observation.^30^ Health-care seeking behaviours have an important effect on the value of outreach interventions. Such behaviours are hard to measure empirically. Additionally, intrinsic care seeking behaviour and the functionality of provider-initiated counselling and testing are hard to distinguish from each other in many contexts. For these reasons, extrapolation of the findings of different studies from different populations into a common framework, as we had to do in this model, is hard. As a result, although each assumption about the interventions is based on a real study, our findings can only be directionally informative. Furthermore, uncertainty increases over the 20 year simulation period, particularly as major changes to health care and treatment are difficult to predict. For example, if a functional cure for HIV were developed or other important changes to treatment made, our results could become obsolete. Although the results presented rely largely on HIV health-care data from western Kenya served by AMPATH, we believe that our results will have the same broad relevance to other settings with large generalised epidemics in rural areas with an established ART programme.

Findings from other modelling studies that have relied on aggregated routine data to provide insight into care are in broad agreement with our results. For example, a similar modelling study focusing on South Africa has shown the potential impact and cost-effectiveness of a combination of interventions strengthening the cascade.^31^ Additionally, other modelling studies have described the cost-effectiveness of immediate ART (Rwanda)^32^ and treatment re-initiation interventions (South Africa).^33^ These differences in intervention cost-effectiveness emphasise variations in the state of care by location and the risk of directly comparing model outputs resulting from potential inconsistencies in approaches and assumptions.

Aspirations for HIV care and treatment have increased rapidly in recent years. The UNAIDS 90-90-90 strategy set out three ambitious targets to be achieved by 2020: 90% of people with HIV diagnosed, 90% of those on treatment, and 90% of them virally suppressed.^13^ Some countries are already moving ahead to universal eligibility for ART;^1^, ^34^ new data are emerging on clinical benefits of ART,^35^ many studies on cascade interventions are being reported,^8^ and WHO has recently released new guidance for ART programmes encouraging immediate initiation of treatment for all individuals positive for HIV.^11^ As countries move towards these targets and consider moves to new guidelines, integrating all available care cascade data with the perspective of improving health for the population is going to be especially important. Our results suggest that there is substantial scope for programmes to improve population health and that alternative sets of strategies are available that will be consistent with their particular aims and budget.

For more information on the **HIV Modelling Consortium** see http://www.hivmodelling.org

## Figures and Tables

**Table 1:** Summary of agreement between AMPATH data and the model

	**AMPATH data**			**Model**		
	2007–10	2010–11	2011–14	2007–10	2010–11	2011–14
**People diagnosed with HIV who entered care by route into care**						
HBCT	..	7%	60%	..	13%	64%
VCT	66%	47%	20%	65%	48%	20%
PICT	34%	46%	20%	35%	39%	16%
**Proportion of individuals in CD4 strata at ART initiation**						
>500 cells per μL	9%	14%	19%	10%	12%	9%
350–500 cells per μL	7%	8%	18%	11%	12%	9%
200–350 cells per μL	18%	21%	41%	14%	12%	46%
<200 cells per μL	66%	57%	22%	65%	64%	36%

AMPATH data were analysed in three discrete time periods: 2007–10 marking the period of time before household-based testing where individuals could only seek care through VCT or PICT, 2010–11 in which HBCT was rolled out in Bunyala (Kenya), and 2011–14 when HBCT was fully implemented and treatment eligibility guidelines had been updated to less than 350 cells per μL or WHO stage 3–4. See appendix pp 25–28 for corresponding figures. AMPATH=Academic Model Providing Access To Healthcare. HBCT=home-based counselling and testing. VCT=voluntary counselling and testing. PICT=provider-initiated counselling and testing. ART=antiretroviral therapy.

**Table 2:** Summary of individual interventions designed to target various aspects of care

	**Assumptions**	**Cost (2013 US$)**
HBCT (passive referral)	Every 4 years, 90% testing coverage; 30% linked to care immediately if not previously diagnosed; 40% if previously diagnosed	$18·00 per HBCT person tested ($8·00 home visit^9^* and $10·00 rapid HIV test^16^)
Enhanced counselling and testing	The rate of HIV testing is 125% that of baseline	$50·00 per person tested ($28·00 clinic visit,^17^ $10·00 rapid HIV test,^16^ and $12·00 CD4 laboratory test^17^)
HBCT (with point-of-care CD4)	Every 4 years, 90% testing coverage of population; 65% linked to care if not previously diagnosed, 70% if previously diagnosed (point-of-care CD4 reduces non-linkage by 50%)	$60·00 per HBCT person tested ($8·00 home-visit,^9^* $10·00 rapid HIV-test,^16^ and $42·00 point-of-care CD4 test^18^)
Facilitated linkage	The risk of failure-to-link is reduced by 50%	$2·61 per diagnosed but not linked patient per year^19^
VCT point-of-care CD4	At VCT, a point-of-care CD4 test is given to patients reducing the risk of not linking to 0%	$80·00 per point-of-care CD4 test ($28·00 clinic visit,^17^ $10·00 rapid HIV test,^16^ and $42·00 point-of-care CD4 test^18^
Pre-ART outreach	In the middle of each year, 20% of tested individuals lost from pre-ART care are sought and returned	$19·55 per patient sought^20^
Improved care	The risk of a patient missing an appointment is reduced by 50%	$7·05 per patient per clinic visit^21^, ^22^
Point-of-care CD4	A point-of-care CD4 test removes the 10% disengagement from care between CD4 test and receiving result	$70·00 per point-of-care CD4 test ($28·00 clinic visit^17^ and $42·00 point-of-care CD4 test^18^)
On-ART outreach	In the middle of each year, 40% of patients who had initiated ART and were lost from care are sought and returned	$19·55 per patient sought.^20^
Adherence	At ART initiation, adherence to ART increases by 50%	$33·54 per person on ART per year^23^
Immediate ART	No pre-ART care, all individuals who enter care are initiated onto ART immediately	Only additional costs due to increased usage of ART (appendix p 45)
Universal test and treat	Immediate ART and HBCT (every 4 years, 90% testing coverage. 30% linked if not previously diagnosed, 40% if previously diagnosed)	$18·00 per HBCT person tested ($8·00 home visit^9^* and $10·00 rapid HIV-test^16^)

When HBCT is applied in isolation in the model this incorporates only a passive referral of patients. Universal test and treat is a combination of HBCT and immediate ART. All interventions except immediate ART and universal test and treat, were considered when identifying the optimal combination of interventions acting on the cascade by selecting interventions with the lowest cost per disability-adjusted life-year averted. HBCT=home-based counselling and testing. VCT=voluntary counselling and testing. ART=antiretroviral therapy.

* Secondary analysis of data from van Rooyen and colleagues*;*^9^ see appendix p 48 for further details.

**Table 3:** DALYs averted and additional cost of care for individual interventions between 2010 and 2030

	**DALYS averted between 2010 and 2030 (million)**	**Additional cost between 2010 and 2030 (2013; million US$)**	Cost per DALY averted compared with baseline (ACER)*	**AIDS deaths averted (%)**
HBCT	0·96	$2241·11	$2324·76	11·56
Enhanced counselling and testing	0·24	$253·76	$1062·06	2·94
HBCT (with point-of-care CD4)	1·61	$2600·75	$1616·71	19·12
Facilitated linkage	0·10	$39·75	$383·97	1·02
VCT point-of-care CD4	0·13	$63·93	$474·19	1·46
Pre-ART outreach	0·26	$217·74	$822·99	3·81
Improved care	0·16	$156·74	$1008·24	2·01
Point-of-care CD4	0·11	$107·40	$953·35	1·66
On-ART outreach	0·71	$355·92	$499·41	13·85
Adherence	0·46	$364·41	$787·45	5·56
Immediate ART	0·62	$552·02	$895·12	8·32
Universal test and treat	1·60	$2813·84	$1760·10	19·11

DALY=disability-adjusted life-year. ACER=average cost-effectiveness ratio. HBCT=home-based counselling and testing. VCT=voluntary counselling and testing. ART=antiretroviral therapy.

* Calculations had an SE of US$150.

**Table 4:** DALYs averted and additional cost of implementing a combination of interventions between 2010 and 2030

	**DALYs averted (million)**	**Additional cost relative to baseline (2013; million US$)**	ICER*	**ACER**†
Facilitated linkage	0·10	$39·75	$383·97	$383·97
Facilitated linkage and on-ART outreach	0·81	$406·78	$518·30	$501·17
Facilitated linkage, on-ART outreach, and VCT point-of-care CD4	0·88	$457·03	$783·02	$521·82
Facilitated linkage, on-ART outreach, VCT point-of-care CD4, pre-ART outreach	1·09	$623·33	$774·53	$571·57
Facilitated linkage, on-ART outreach, VCT point-of-care CD4, pre-ART outreach, and point-of-care CD4	1·10	$630·99	$543·24	$571·21
Facilitated linkage, on-ART outreach, VCT point-of-care CD4, pre-ART outreach, point-of-care CD4, and immediate ART	1·68	$1123·06	$852·10	$667·64

Intervention results in table 3 cannot be combined additively to arrive at those listed above because these results are generated with a dynamic model. Interventions were considered cost-effective if ACER was less than 50% of GDP per capita for Kenya in 2013 (US$1242).^24^ DALY=disability-adjusted life-year. ICER=incremental cost-effectiveness ratio. ACER=average cost-effectiveness ratio. ART=antiretroviral therapy. VCT=voluntary counselling and testing.

* Cost per DALY averted compared with previous increment.

† Cost per DALY averted compared with baseline.
