# Supplementary material for: Evaluating strategies to improve HIV care outcomes in Kenya: a modelling study
Source: Lancet HIV. 2016 Oct 19;3(12):e592–600. doi: 10.1016/S2352-3018(16)30120-5 (PMC5121132; doi:10.1016/S2352-3018(16)30120-5)
Supplement: Supplementary appendix [file mmc1.pdf]

Evaluating Strategies to Improve HIV Care Outcomes in Kenya

Appendix

Contents

1 Model Description **5**
- 1.1 Demography . . . 5
- 1.2 Model Overview . . . 8

2 HIV Incidence **9**

3 HIV Natural History Model **12**
- 3.1 Structure . . . 12
- 3.2 Calibration . . . 12

4 Cascade Model **19**
- 4.1 Structure . . . 19
- 4.2 Calibration . . . 19
  - 4.2.1 AMPATH Data . . . 19
  - 4.2.2 HBCT . . . 24
  - 4.2.3 Results to AMPATH Data . . . 25
  - 4.2.4 Results to Province-Level Estimates . . . 29
  - 4.2.5 Uniform Care Seeking Calibration: Results to AMPATH Data . . . 31
  - 4.2.6 Uniform Care Seeking Calibration: Results to Province-Level Estimates . . . 36
  - 4.2.7 Disease-Led Care Seeking Calibration: Results to AMPATH Data . . . 38
  - 4.2.8 Disease-Led Care Seeking Calibration: Results to Province-Level Estimates . . . 43

5 Cost **45**

6 DALYs **47**

7 Interventions **48**
- 7.1 Intervention Detail . . . 48
- 7.2 Uniform Care Seeking Calibration: Results . . . 49
- 7.3 Disease-Led Care Seeking Calibration: Results . . . 50
- 7.4 No Transmission Benefit Scenario: Results . . . 51
- 7.5 Sources of Mortality . . . 52

8 Sensitivity Analysis **53**
- 8.1 Methods . . . 53
- 8.2 Results . . . 53

9 HBCT Analysis **56**

List of Figures

S1 **Survival curves calculated from non HIV-related mortality rates from United Nations World Population Prospects Database.[1]** . . . 5
S2 **Modelled incidence against estimates from Spectrum.** . . . 10
S3 **Natural history model flow diagram.** HIV progression through CD4 categories (>500, 350-500, 200-350 and <200 cells/μl) and WHO Stages (I, II, III, IV) depends on an individuals treatment status. If an individual initiates ART but subsequently stops, they transition back to the pre-ART side of the model. . . . 12
S4 **Natural history model calibration results compared to original data sources.** . . . 16
S4 **Natural history model calibration results compared to original data sources (continued).** . . . 17
S4 **Natural history model calibration results compared to original data sources (continued).** . . . 18
S5 **Map of Ministry of Health-AMPATH Clinic Sites in Western Kenya.** Port Victoria, Bunyala, is 26 (blue circle). Modified image from: `http://ampath-uoft.ca/about-us/activities/map/`. . . . 20
S6 **Proportion of infected individuals aware of HIV-status by 2010.** . . . 25
S7 **Distribution of individuals across the three routes of entry into care.** . . . 25
S8 **CD4 distribution at entry to care.** . . . 26
S9 **CD4 distribution at ART initiation.** . . . 26
S10 **Proportion of individuals initiating ART per year.** . . . 27
S11 **Prior care experience among individuals initiating ART.** . . . 28
S12 **Comparison of HIV-prevalence in the model to KAIS 2012 estimates from Western Province.** 29
S13 **Comparison of HIV-prevalence in the model to AMPATH estimates from Bunyala in 2014.** . 29
S14 **Comparison to UNAIDS estimates from Spectrum.[2]** . . . 30
S15 **Uniform Care Seeking Calibration - Proportion of infected individuals aware of HIV-status by 2010.** . . . 32
S16 **Uniform Care Seeking Calibration - Distribution of individuals across the three routes of entry into care.** . . . 32
S17 **Uniform Care Seeking Calibration - CD4 distribution at entry to care.** . . . 33
S18 **Uniform Care Seeking Calibration - CD4 distribution at ART initiation.** . . . 33
S19 **Uniform Care Seeking Calibration - Prior care experience among individuals initiating ART.** 34
S20 **Uniform Care Seeking Calibration - Proportion of individuals initiating ART per year.** . . . 35
S21 **Uniform Care Seeking Calibration - Comparison of HIV-prevalence in the model to KAIS 2012 estimates from Western Province.** . . . 36
S22 **Uniform Care Seeking Calibration - Comparison of HIV-prevalence in the model to AMPATH estimates from Bunyala in 2014.** . . . 36
S23 **Uniform Care Seeking Calibration - Comparison to UNAIDS estimates from Spectrum.[2]** . . 37
S24 **Disease-Led Care Seeking Calibration - Proportion of infected individuals aware of HIV-status by 2010.** . . . 38
S25 **Disease-Led Care Seeking Calibration - Distribution of individuals across the three routes of entry into care.** . . . 39
S26 **Disease-Led Care Seeking Calibration - CD4 distribution at entry to care.** . . . 39
S27 **Disease-Led Care Seeking Calibration - CD4 distribution at ART initiation.** . . . 40
S28 **Disease-Led Care Seeking Calibration - Prior care experience among individuals initiating ART.** . . . 41
S29 **Disease-Led Care Seeking Calibration - Proportion of individuals initiating ART per year.** . 42
S30 **Disease-Led Care Seeking Calibration - Comparison of HIV-prevalence in the model to KAIS 2012 estimates from Western Province.** . . . 43
S31 **Disease-Led Care Seeking Calibration - Comparison of HIV-prevalence in the model to AMPATH estimates from Bunyala in 2014.** . . . 43
S32 **Disease-Led Care Seeking Calibration - Comparison to UNAIDS estimates from Spectrum.[2]** 44
S33 **Flow diagram describing the cost function in the model.** The cost of the individual components of care accumulate over time and additional costs are incurred when interventions are implemented. . . . 46
S34 **DALYs averted and additional cost of care for individual interventions between 2010 and 2030 when care seeking behaviour is not a function of current health status or previous care experience.** . . . 49

S35 **DALYs averted and additional cost of care for individual interventions between 2010 and 2030 when care seeking behaviour is only a function of current health state.** 50
S36 **DALYs averted and additional cost of care for individual interventions between 2010 and 2030 where interventions provide no transmission benefits.** 51
S37 **Care experience of deceased individuals who suffered an HIV-related death between 2010 and 2030.** Cost at the top of each bar is the total cost of care for all individuals between 2010 and 2030 for each scenario. 52
S38 **DALYs averted and additional cost of care for interventions acting on the cascade between 2010 and 2030.** The sensitivity of each result to variations in the unit cost of each component of care is also illustrated. 54
S39 **Ranked cost-effectiveness of interventions (cost per DALY averted between 2010 and 2030).** One thousand random draws of unit costs for each intervention. The mean cost-effectivenss is denoted by the largest dot. 55
S40 **DALYs averted and additional cost of care for individual interventions between 2010 and 2030.** Here, we simulate an additional HBCT intervention with active referral, whereby 90% of diagnosed individuals are linked to care. 56

List of Tables

S1 **Population size and growth in Kenya.** 6
S2 **Age distribution of Kenya in 1970 stratified by sex.** 7
S3 **Incident cases of HIV per year.** 9
S4 **Incident Rate Ratio's (IRR's) stratified by age and sex.** 9
S5 **Infectiousness weights by health state.** 10
S6 **Data sources for natural history calibration of the pre-ART model.** 13
S7 **Data sources for natural history calibration of the ART model.** 14
S8 **Natural history model parameter definitions and fitted values.** 15
S9 **Questions used to interrogate the AMRS database for calibration of the cascade model.** . . . 21
S10 **Summary of parameters used in the cascade model.** 22
S11 **Summary of parameters used in the cascade model (continued).** 23
S12 **Summary of updated parameters used in the Uniform Care Seeking calibration.** 31
S13 **Summary of updated parameters used in the Disease-Led Care Seeking calibration.** 38
S14 **HIV-care cost breakdown.** 45
S15 **Disability weights by health state.** 47
S16 **Evidence of indicative intervention.** 48
S17 **Costs associated with individual components of care.** Row names with an * are intervention specific. All costs are in 2013 USD. 53
S18 **Ranked table of interventions.** Interventions are ranked in order of increasing cost-effectiveness (cost per DALY averted). 54

1 Model Description

An individual-based micro-simulation was created in C++ to model the HIV epidemic in western Kenya and capture the care experience of HIV-positive individuals progressing through the various stages of an ART programme.

1.1 Demography

The model describes the population of Kenya from 1970 to 2030. To begin, a cohort of individuals is created that captures the age distribution and size of the population of Kenya in 1970. Thereafter, annual population growth is captured through births uniformally distributed throughout the year.

The initial population size in the model, together with population growth is driven by data from The World Bank from 1970 onwards.[3] We initially populate the model with the correct population size for Kenya in 1970 (Table S1), along with the correct age distribution for that year (Table S2). We then calculate population growth from 1971 onwards to estimate the annual increase in population size (Table S1). This is then used to specify the number of births from 1971 onwards. However, World Bank population projections cease in 2020; and from this year onwards population growth is held constant.

The sex ratio in Kenya is defined as 1:1 by the Central Intelligence Agency World Factbook.[4] Natural life-expectancy is determined at birth using mortality rates from the United Nations World Population Prospects Database (Fig S1).[1] Acquisition of HIV is driven by incidence estimates from UNAIDS, available from Spectrum (Section 1.2 & 2).[2] Once infected, HIV-related life expectancy and disease progression are dependent upon current CD4 count and WHO stage (Section 3).

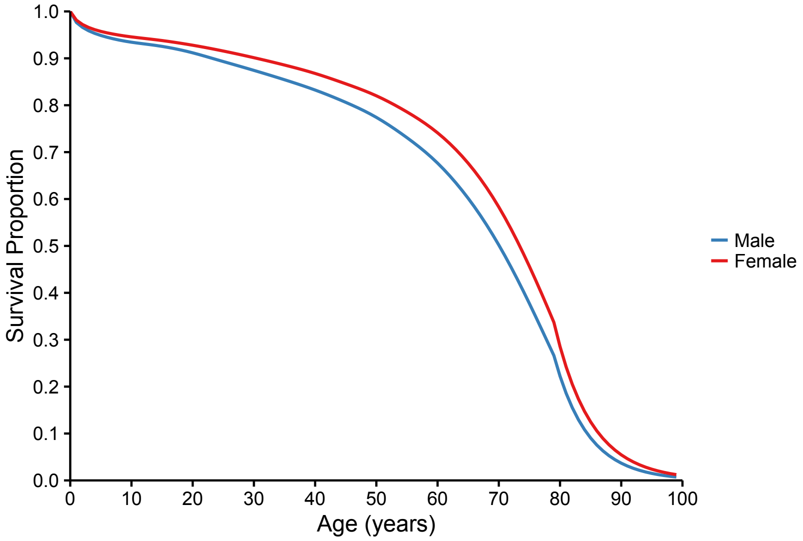

Figure S1. Survival curves calculated from non HIV-related mortality rates from United Nations World Population Prospects Database.[1]

Table S1. Population size and growth in Kenya.

Year	Population Size	Growth Since Previous Year	Increase in Population Size Since Previous Year
1970	11,252,466	-	11,252,466
1971	11,653,161	3.56%	400,695
1972	12,072,791	3.60%	419,630
1973	12,513,032	3.65%	440,241
1974	12,973,337	3.68%	460,305
1975	13,454,455	3.71%	481,118
1976	13,956,681	3.73%	502,226
1977	14,480,389	3.75%	523,708
1978	15,027,062	3.78%	546,673
1979	15,597,909	3.80%	570,847
1980	16,192,118	3.81%	594,209
1981	16,811,300	3.82%	619,182
1982	17,455,742	3.83%	644,442
1983	18,122,818	3.82%	667,076
1984	18,805,615	3.77%	682,797
1985	19,501,240	3.70%	695,625
1986	20,210,650	3.64%	709,410
1987	20,933,141	3.57%	722,491
1988	21,664,272	3.49%	731,131
1989	22,399,180	3.39%	734,908
1990	23,165,081	3.42%	765,901
1991	23,941,680	3.35%	776,599
1992	24,726,053	3.28%	784,373
1993	25,509,128	3.17%	783,075
1994	26,274,354	3.00%	765,226
1995	26,997,312	2.75%	722,958
1996	27,715,841	2.66%	718,529
1997	28,423,951	2.55%	708,110
1998	29,139,406	2.52%	715,455
1999	29,868,767	2.50%	729,361
2000	30,619,430	2.51%	750,663
2001	31,394,794	2.53%	775,364
2002	32,194,766	2.55%	799,972
2003	33,009,355	2.53%	814,589
2004	33,854,958	2.56%	845,603
2005	34,711,022	2.53%	856,064
2006	35,592,109	2.54%	881,087
2007	36,507,978	2.57%	915,869
2008	37,454,707	2.59%	946,729
2009	38,437,769	2.62%	983,062
2010	39,466,361	2.68%	1,028,592
2011	40,521,914	2.67%	1,055,553
2012	41,602,357	2.67%	1,080,443
2013	42,698,936	2.64%	1,096,579
2014	43,805,499	2.59%	1,106,563
2015	44,918,079	2.54%	1,112,580
2016	46,035,724	2.49%	1,117,645
2017	47,161,767	2.45%	1,126,043
2018	48,298,133	2.41%	1,136,366
2019	49,446,272	2.38%	1,148,139
2020	50,610,060	2.35%	1,163,788
2021	51,801,240	2.35%	1,191,180
2022	53,020,455	2.35%	1,219,216
2023	54,268,367	2.35%	1,247,912
2024	55,545,650	2.35%	1,277,283
2025	56,852,996	2.35%	1,307,346
2026	58,191,112	2.35%	1,338,116
2027	59,560,722	2.35%	1,369,610
2028	60,962,569	2.35%	1,401,846
2029	62,397,409	2.35%	1,434,841
2030	63,866,021	2.35%	-

Data from The World Bank.[3]

Table S2. Age distribution of Kenya in 1970 stratified by sex.

Age Category	Male Probability	Female Probability
0 to 4	0.1016	0.1012
5 to 9	0.0791	0.0797
10 to 14	0.0641	0.0648
15 to 19	0.0520	0.0527
20 to 24	0.0385	0.0389
25 to 29	0.0274	0.0278
30 to 34	0.0250	0.0257
35 to 39	0.0237	0.0237
40 to 44	0.0200	0.0193
45 to 49	0.0166	0.0154
50 to 54	0.0139	0.0130
55 to 59	0.0118	0.0108
60 to 64	0.0096	0.0092
64 to 69	0.0069	0.0077
70 to 74	0.0046	0.0056
75 to 79	0.0025	0.0034
$>$80	0.0015	0.0023

Data from United Nations World Population Prospects.[5]

1.2 Model Overview

The model simulates the lives of individuals concurrently until death (either naturally or from AIDS). Throughout an individual's lifetime, new events may occur (i.e. they acquire HIV). This model allows events to be scheduled and executed dynamically according to a set of rules defined by each event.

All individuals enter the model with an HIV-negative serostatus. We model the initial spread of HIV infection among our population based on national HIV incidence estimates published by UNAIDS (Section 2). Upon acquisition of HIV, we model HIV progression through declining CD4 count and the development of WHO stage infections (Section 3). We allow HIV-tests to occur from 2004 onwards to mimic the rollout of HIV-testing in Kenya. Care seeking behaviour is driven by current health status and previous experience of care. We model in detail each stage of HIV care and allow eligible individuals to initiate treatment from 2004 onwards (Section 4). Treatment eligibility guidelines in 2004 are a CD4 count of $<$200 cells/μl or WHO Stage IV.[6] These are updated in 2011 to a CD4 count of $<$350 cells/μl or WHO Stage III/IV as per Kenyan Guidelines.[7]

The model is a time-to-event simulation, and contains a chronologically ordered queue of events to be executed. When an individual is created, the model records the calendar-time at which they enter and calculates a natural death date by taking into consideration the number of years an individual will likely live (Fig S1). Life history events are then executed chronologically until an individual dies. Events can take the form of natural history events (such as the birth or death of an individual) or HIV care related events (such as an HIV-test or ART initiation).

After an event is executed, the state of an individual may change (e.g. if an individual receives a CD4 test result confirming their eligibility for treatment), the model senses this change and schedules the appropriate event (e.g. an ART initiation event). New events enter the event-queue and the model continues scheduling and executing events until time reaches 2030. At this point the model stops and produces a list of outputs from the simulation.

Source code and relevant scripts to replicate results are available at: `https://github.com/olneyjack/CareCascade`

2 HIV Incidence

In the model, from 1970 to 2002, the number of new HIV infections is determined by national estimates published by UNAIDS, sourced from the Spectrum software by Avenir Health.[2] Spectrum provides us with the number of incident infections that are estimated to have occurred in a particular year (Table S3). We then divide these infections amongst the population by age and sex, according to age and sex specific incidence rate ratios (IRR's) also provided by Spectrum (Table S4).

Table S3. Incident cases of HIV per year.

Year	Incident Cases
1980	140
1981	355
1982	1,134
1983	1,791
1984	3,418
1985	6,444
1986	11,887
1987	21,704
1988	38,623
1989	66,784
1990	108,993
1991	165,074
1992	226,131
1993	269,547
1994	275,327
1995	243,681
1996	195,612
1997	152,571
1998	121,318
1999	101,327
2000	99,767
2001	93,594

Estimates sourced from Spectrum.[2]

Table S4. Incident Rate Ratio's (IRR's) stratified by age and sex.

Age category	Male	Female
0 to 4	0	0
5 to 9	0	0
10 to 14	0	0
15 to 19	0.244859	0.431475
20 to 24	0.790423	0.979206
25 to 29	1	1
30 to 34	0.989385	0.848891
35 to 39	0.854318	0.684447
40 to 44	0.670484	0.550791
45 to 49	0.493512	0.440263
50 to 54	0.358977	0.336719
55 to 59	0.282399	0.239474
60 to 64	0.259244	0.16789
64 to 69	0.264922	0.146594
70 to 74	0.254788	0.171352
75 to 79	0.164143	0
>80	0	0

Estimates sourced from Spectrum.[2]

At the beginning of each year we calculate the number of new infections (I) occurring in each age category (a) and sex category (s) through the following equation:

$$I_{a,s} = \lambda \cdot S_{a,s} \cdot IRR_{a,s} \tag{1}$$

Where S is the number of susceptible individuals stratified by age category (a) and sex category (s). IRR is the incidence rate ratio for the corresponding age and sex category from table S4. λ is the transmission rate and is calculated annually through dividing the number of incident cases in the current year (from table S3) by the sum of all susceptible individuals multiplied by their IRR in each age and sex category as follows:

$$\lambda = \frac{Incident\ Cases}{\sum_{a,s}(S_{a,s} \cdot IRR_{a,s})} \tag{2}$$

We model the impact of interventions on incidence by weighting the infectiousness of HIV-positive individuals and deriving a transmission probability. We calculate the transmission probability in 2002 as by this time in the epidemic incidence has already peaked, and is relatively stable (Fig S2).

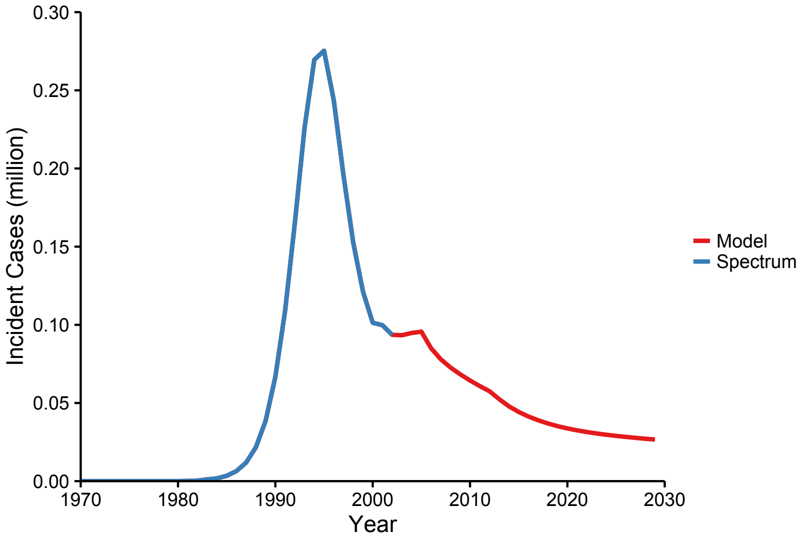

Figure S2. Modelled incidence against estimates from Spectrum.

From 2002 onwards, incidence is driven by the weighted infectiousness of HIV-positive individuals multiplied by the transmission probability (Equation 3); where β is the transmission probability, w is the infectiousness weighting and I is the number of HIV-positive individuals in each infectiousness category k (Table S5).

$$Incident\ Cases = \beta \sum_{all\ k} (w_k \cdot I_k) \tag{3}$$

Table S5. Infectiousness weights by health state.

**Health State Category**	**Infectiousness Weight (w)**	**Source**
HIV-positive, CD4 count >500 cells/μl (untreated)	1.35	Based on 3 months with acute infection and 6.25 years at CD4 >500, and 10-fold infectious with acute infection
HIV-positive, CD4 count 350-500 cells/μl (untreated)	1.00	Reference cell
HIV-positive, CD4 count 200-350 cells/μl (untreated)	1.64	Donnell *et al.* (2010)[8]
HIV-positive, CD4 count <200 cells/μl (untreated)	5.17	Donnell *et al.* (2010)[8]
HIV-positive, on ART and virally suppressed	0.1	Estimate

However, we first need to calculate the transmission probability. Therefore, we rearrange equation 2 and extract the value of β on the 31st of December 2001 (Equation 4).

$$\beta = \frac{Incident\ Cases}{\sum_{all\ k}(w_k \cdot I_k)} \tag{4}$$

With the value of β fixed, we then alter the calculation of λ to:

$$\lambda = \frac{\beta \sum_{all\ k}(w_k \cdot I_k)}{\sum_{a,s}(S_{a,s} \cdot IRR_{a,s})} \tag{5}$$

This equation is used from 1st January 2002 onwards and allows the transmission rate to be controlled by the number of infectious individuals in the model. The new definition λ is used in the calculation of new infections by age and sex category and replaces the previous definition in equation 2. Thus, when interventions are implemented from 2010, the indirect impact of these interventions on reducing HIV incidence is accounted for by allowing incidence to be driven by the infectiousness of individuals in the population.

3 HIV Natural History Model

3.1 Structure

Upon acquiring HIV, an individual is assigned an initial CD4 count category (<200, 200-350, 350-500, >500 cells/µl) and is assumed to begin with WHO Clinical Stage I infection.[9] We capture HIV progression by describing CD4 count decline and the acquisition of WHO Stage defining conditions. For example, progression from CD4 >500 and WHO Stage I is captured by competing hazards of health decline to CD4 350-500 and also to WHO Stage II infection.

Additionally, individuals are exposed to a CD4 & WHO Stage specific HIV-related mortality hazard. This hazard also competes with the hazards of CD4 decline and WHO Stage progression. HIV-related mortality hazards are independent of natural mortality. If the model encounters the HIV-related death date prior to encountering the natural death date, the individual has suffered an HIV-related death.

All individuals acquiring HIV first progress through the pre-ART side of the natural history model (Fig S3). Once individuals become eligible for treatment, and have progressed through the relevent care stages (Section 4), and if they adhere to ART, they transition to the on-ART side of the natural history model. Propensity to adhere to ART is a person-specific characteristic that is determined for each individual at the time they enter the model. When transitioning to the on-ART side of the natural history model, individuals stay in the same health statae (i.e. when an individual with CD4 <200 and in WHO Stage III initiates ART, they move into the <200 WHO Stage III category of the on-ART model).

Once an individual initiates and adheres to treatment, their CD4 decline reverses and they can recover from any WHO Stage defining conditions. It should be noted that WHO Stage conditions can still develop on this side of the model; thus allowing the model to capture potential failures of treatment among patients adhering to ART. Additionally, if a patient's CD4 count falls below 500 cells/µl prior to ART initiation, their CD4 count will not recover to more than 500 cells/µl if treatment is initiated. This assumption was made in response to findings by Lawn *et al.* (2006) illustrating CD4 count reconstitution among patients initiating ART in Cape Town, South Africa.[10] The mortality hazard for an individual of a particular health state on the on-ART side of the model is less than that of the same individual on the pre-ART side of the model, thereby giving ART a survival advantage (Section 3.2). Natural history parameters are shown in fig S3 with corresponding definitions and values detailed in table S8.

3.2 Calibration

Calibration of the natural history model involved developing a deterministic version of the natural history model and calibrating it to all available surveillance data from the literature using least square regression in Berkeley Madonna.[11] To begin, the literature was reviewed to identify relevant studies that would enable us to calibrate all

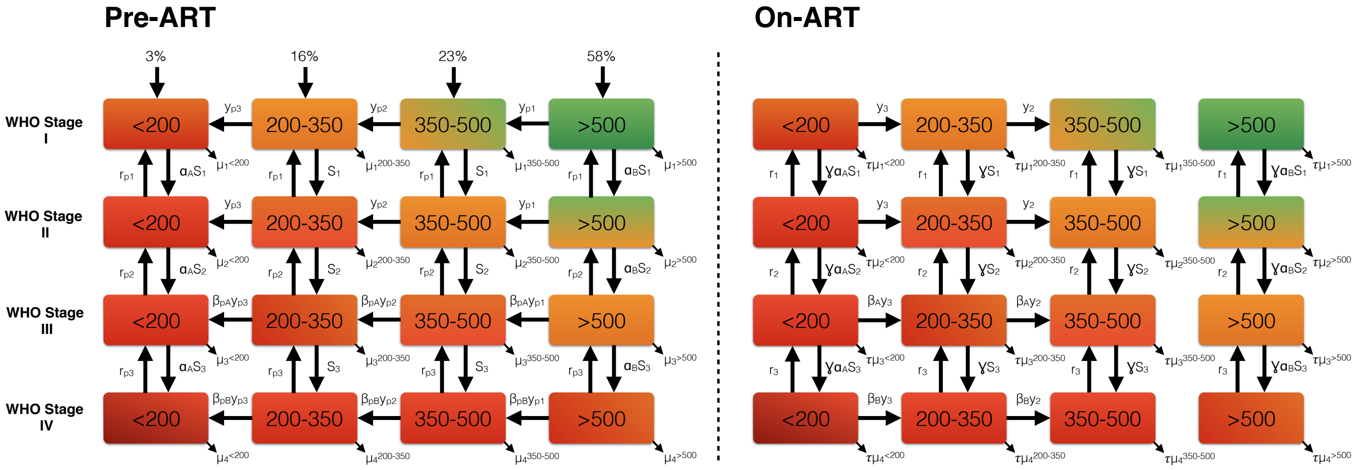

Figure S3. Natural history model flow diagram. HIV progression through CD4 categories (>500, 350-500, 200-350 and <200 cells/µl) and WHO Stages (I, II, III, IV) depends on an individuals treatment status. If an individual initiates ART but subsequently stops, they transition back to the pre-ART side of the model.

aspects of the natural history model. Where possible, data from cohort studies was utilised; although, in some situations data from cross-sectional studies was used. Data sources are listed below in tables S6 and S7.

Table S6. Data sources for natural history calibration of the pre-ART model.

Outcome	Location	Description	Conditions	Use in model	Reference
Pre-ART survival	East Africa & South Africa	Survival function (calculated from model fits with Weibull distribution)	25-29 years old, 4 years between last negative and first positive HIV test	Fitting pre-ART survival curve	Todd *et al.* (2007)[12]
CD4 count decline	Europe	Progression rates for each CD4 category (calculated by fitting compartmental model to estimates by Eaton & Fraser, *unpublished*)	≥16 years old, maximum of 3 years between last negative and first positive test	Fitting CD4 >500 to 350-500 progression rate, 350-500 to 200-350 progression rate and 200-350 to <200 progression rate	Lodi *et al.* (2011)[13]
Initial distribution of CD4 counts	Europe	Distribution of individuals across CD4 categories after seroconversion (estimated by Eaton & Fraser, *unpublished*)	≥16 years old, maximum of 3 years between last negative and first positive test	Initial distribution of individuals across pre-ART CD4 categories	Lodi *et al.* (2011)[13]
Progression through WHO clinical stages	Vancouver	Progression rates through WHO Clinical Stages	Seroincident homosexual men	Fitting WHO I to WHO II progression rate, WHO II to WHO III progression rate and WHO III to WHO IV progression rate	Schechter *et al.* (1995)[14]
	Europe & Australia	Rate of AIDS events (WHO Stage III/IV conditions) stratified by CD4 count	Treatment naive individuals or those treated in zidovudine monotherapy era, AIDS-free with at least 1 day of active follow-up with viral load and CD4 count available	Fitting pre-ART WHO stage progression and recovery rates	Cascade Collaboration *et al.* (2004)[15]
Pre-ART mortality rates stratified by CD4 count & WHO clinical stage	Cape Town, South Africa	Mortality rates by CD4 count crossed with WHO Clinical Stage	Individuals not receiving antiretroviral therapy	Fitting pre-ART mortality rates by CD4 count and WHO Clinical Stage	Badri *et al.* (2006)[16]
	Rural south-west Uganda	Median survival time from when first seen in a specific WHO Clinical Stage	Seroconverted in last 7 years	Fitting pre-ART survival by WHO Clinical Stage	Morgan *et al.* (1997)[17]
	Rural south-west Uganda	Median survival time from when first seen in a specific WHO Clinical Stage	Seroconverted in last 7 years	Fitting pre-ART survival by WHO Clinical Stage	Malamba *et al.* (1999)[18]
Person-time spend in each CD4 category and WHO clinical stage	Rakai, Uganda	CD4 category distribution (<200, >200) stratified by WHO Clinical Stage (1 & 2, 3 & 4) and vice versa	Not mentioned	Average person-time spent in each CD4 category, stratified by WHO Clinical Stage	Kagaayi *et al.* (2007)[19]
	Addis Ababa, Ethiopia	CD4 category distribution (<200, 200-499, >500) stratified by WHO Clinical Stage (1,2,3,4) and vice versa	18-44 years old	Average person-time spent in each CD4 category, stratified by WHO Clinical Stage	Kassa *et al.* (1999)[20]
	Jinja, Uganda	CD4 category distribution (<200, >200 and <350, >350) stratified by WHO Clinical Stage (1 & 2, 3 & 4) and vice versa	None mentioned	Average person-time spent in each CD4 category, stratified by WHO Clinical Stage	Jaffar *et al.* (2008)[21]
	Mengo, Jinja and Kasana in Uganda	CD4 category distribution (<200, >200 and <350, >350) stratified by WHO Clinical Stage (1,2,3,4) and vice versa	≥18 years old and ART naive	Average person-time spent in each CD4 category, stratified by WHO Clinical Stage	Baveewo *et al.* (2011)[22]
Pre-ART mortality stratified by WHO clinical stage	Rural south-west Uganda	Cumulative mortality at year 1 and year 4, crossed by WHO Clinical Stage	Seroconverted in last 7 years	Pre-ART cumulative mortality crossed by WHO Clinical Stage at year 1 and 4	Morgan *et al.* (1997)[17]
	Rural south-west Uganda	Survival estimate at 6 years, crossed by WHO Clinical Stage	Seroconverted in last 7 years	Pre-ART survival at year 6, crossed by WHO Clinical Stage	Malamba *et al.* (1999)[18]

These data were then weighted to ensure that each had equal influence during model calibration. Equal weighting of studies ensured that the model could reconcile the large amount of data and find a compromise between any potentially conflicting data.

If we consider the standardised absolute error per study:

$$e_i = \frac{1}{n_i}\sum_{j=1}^{n_i}\frac{|D_{ij}-M_{ij}|}{D_{ij}} \qquad (6)$$

Where D_{ij} refers to the value of datapoint j in study i and M_{ij} refers to the value of the model at datapoint j in study i. It then follows that e_i, is the standardised absolute error in study i, where n_i is the total number of data points in study i. Therefore, the total error across all studies, can be represented by:

$$E = \sum e_i \qquad (7)$$

Using Berkeley Madonna, we fitted the natural history model (as seen in fig S3) to the weighted data points using least squares to minimise the error between each data point and the model. All parameters on the pre-ART and ART side of the natural history were fitted simultaneously giving the model many degrees of freedom

Table S7. Data sources for natural history calibration of the ART model.

Outcome	Location	Description	Conditions	Use in model	Reference
Cumulative Mortality after ART initiation stratified by CD4 count and WHO Clinical Stage	South Africa, Malawi and Côte d'Ivoire	Cumulative mortality stratified by CD4 count and WHO Clinical Stage at 3, 6 and 12 months since ART initiation	Predominantly female population. Median age 34 years. Median body weight of 55kg and median CD4 cell count of 111 cells/µl at baseline	Fitting weighted on-ART cumulative mortality stratified by CD4 count and WHO Clinical Stage. Back-calculated mortality rate also used in fitting	May *et al.* (2010)[23]
Mortality rate after ART initiation stratified by CD4 count	South Africa	On-ART mortality rates stratified by CD4 count <200 and >200 for 0-1 year, 1-2 years, 2-3 years and 3-5 years after ART initiation	Mostly female population with a mean age of 25-35 years old and mean CD4 count of 100-199 cells/µl at baseline	Fitting a weighted average cumulative mortality rate for each period (0-1 year, 1-2 years, 2-3 years and 3-5 years), averaged across all WHO Clinical Stages	Johnson *et al.* (2013)[24]
On-ART mortality rates stratified by CD4 count	Cape Town, South Africa	Mortality rates after ART initiation, stratified by current CD4 count. Median follow-up time was 2.5 years.	Median age at baseline was 33 years, 67% of cohort were female with a median CD4 count of 101 cells/µl at baseline. 53% of cohort had WHO Clinical Stage III defining conditions	Fitting a weighted average on-ART mortality rate stratified by CD4 count at 2.5 years, averaged across all WHO Clinical Stages and weighted to replicate the distribution of CD4 counts and WHO Clinical Stages seen in this papers cohort	Lawn *et al.* (2009)[25]
CD4 count recovery rates after initiation of ART	Cape Town, South Africa	Estimates of CD4 cell count recovery extracted from figure and average time for CD4 count to reach next category from previous calculated	Median age at baseline was 32 years, 75% of cohort were female, median CD4 count was 97 cells/µl and 53% had WHO Clinical Stage III defining conditions	Fitted on-ART CD4 count recovery rates: *y3* (<200 to 200-350) and *y2* (200-350 to 350-500)	Lawn *et al.* (2006)[10]

and ensuring that survival advantage was given to patients on treatment. The resulting model fit produced the parameter values shown in table S8. Due to the complexity of the natural history model and the breadth of data used for calibration, parameter uncertainty was not computed. However, the resulting model fit is a result of the model finding a pragmatic solution that variously agrees with all relevant data, and we therefore feel that a formal analysis of uncertainty is not required. Comparisons between the calibrated model and its data sources are shown in fig S4.

Table S8. Natural history model parameter definitions and fitted values.

Parameter	Definition	Value
y_{p1}	Pre-ART CD4 progression rate from >500 to 350-500 cells/µl	0.1518 $py^{-}1$
y_{p2}	Pre-ART CD4 progression rate from 350-500 to 200-350 cells/µl	0.2398 $py^{-}1$
y_{p3}	Pre-ART CD4 progression rate from 200-350 to <200 cells/µl	0.1947 $py^{-}1$
β_{pA}	Weight applied to Pre-ART CD4 progression rate for patients in WHO Stage III	1.3552
β_{pB}	Weight applied to Pre-ART CD4 progression rate for patients in WHO Stage IV	9.9797
S_1	WHO Stage progression rate from Stage I to II	0.3075 $py^{-}1$
S_2	WHO Stage progression rate from Stage II to III	0.2193 $py^{-}1$
S_3	WHO Stage progression rate from Stage III to IV	0.4132 $py^{-}1$
α_A	Weight applied to WHO Stage progression rate for patients in CD4 category <200	8.6929
α_B	Weight applied to WHO Stage progression rate for patients in CD4 category >500	1
r_{p1}	Pre-ART WHO Stage recovery rate from WHO Stage II to I	3.0525 $py^{-}1$
r_{p2}	Pre-ART WHO Stage recovery rate from WHO Stage III to II	0.0695 $py^{-}1$
r_{p3}	Pre-ART WHO Stage recovery rate from WHO Stage IV to III	0.2973 $py^{-}1$
$\mu_1^{>500}$	Pre-ART Mortality rate for CD4 category >500 and WHO Stage I	0.0012 $py^{-}1$
$\mu_1^{350\text{-}500}$	Pre-ART Mortality rate for CD4 category 350-500 and WHO Stage I	0.0061 $py^{-}1$
$\mu_1^{200\text{-}350}$	Pre-ART Mortality rate for CD4 category 200-350 and WHO Stage I	0.0371 $py^{-}1$
$\mu_1^{<200}$	Pre-ART Mortality rate for CD4 category <200 and WHO Stage I	0.0582 $py^{-}1$
$\mu_2^{>500}$	Pre-ART Mortality rate for CD4 category >500 and WHO Stage II	0.0214 $py^{-}1$
$\mu_2^{350\text{-}500}$	Pre-ART Mortality rate for CD4 category 350-500 and WHO Stage II	0.0285 $py^{-}1$
$\mu_2^{200\text{-}350}$	Pre-ART Mortality rate for CD4 category 200-350 and WHO Stage II	0.0420 $py^{-}1$
$\mu_2^{<200}$	Pre-ART Mortality rate for CD4 category <200 and WHO Stage II	0.1215 $py^{-}1$
$\mu_3^{>500}$	Pre-ART Mortality rate for CD4 category >500 and WHO Stage III	0.0436 $py^{-}1$
$\mu_3^{350\text{-}500}$	Pre-ART Mortality rate for CD4 category 350-500 and WHO Stage III	0.0629 $py^{-}1$
$\mu_3^{200\text{-}350}$	Pre-ART Mortality rate for CD4 category 200-350 and WHO Stage III	0.1386 $py^{-}1$
$\mu_3^{<200}$	Pre-ART Mortality rate for CD4 category <200 and WHO Stage III	0.2621 $py^{-}1$
$\mu_4^{>500}$	Pre-ART Mortality rate for CD4 category >500 and WHO Stage IV	0.1028 $py^{-}1$
$\mu_4^{350\text{-}500}$	Pre-ART Mortality rate for CD4 category 350-500 and WHO Stage IV	0.1312 $py^{-}1$
$\mu_4^{200\text{-}350}$	Pre-ART Mortality rate for CD4 category 200-350 and WHO Stage IV	0.4430 $py^{-}1$
$\mu_4^{<200}$	Pre-ART Mortality rate for CD4 category <200 and WHO Stage IV	0.5610 $py^{-}1$
τ	Weight applied to Pre-ART mortality rates in on-ART model; giving survival advantage to on-ART model	0.95
γ	Weight applied to WHO Stage progression rates in on-ART model	0.8930
y_2	On-ART CD4 reconstitution rate from 200-350 to 350-500 cells/µl	2.2319 $py^{-}1$
y_3	On-ART CD4 reconstitution rate from <200 to 200-350 cells/µl	4.9467 $py^{-}1$
β_A	Weight applied to On-ART CD4 reconstitution rate for patients in WHO Stage III	0.5872
β_B	Weight applied to On-ART CD4 reconstitution rate for patients in WHO Stage IV	0.2459
r_1	On-ART WHO Stage recovery rate from WHO Stage II to I	1.5273 $py^{-}1$
r_2	On-ART WHO Stage recovery rate from WHO Stage III to II	3.0552 $py^{-}1$
r_3	On-ART WHO Stage recovery rate from WHO Stage IV to III	20.2202 $py^{-}1$

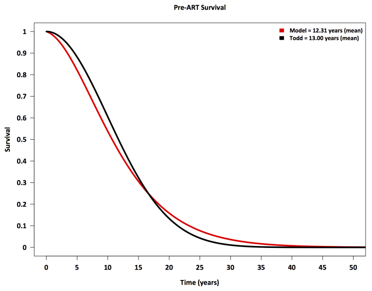

(**A**) Pre-ART survival – Todd *et al.* (2007)[12]

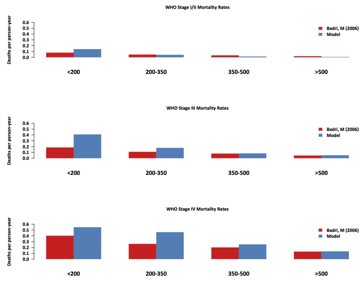

(**B**) Pre-ART mortality rates – Badri *et al.* (2006)[16]

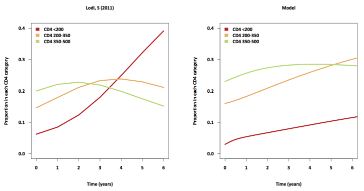

(**C**) Pre-ART CD4 progression – Lodi *et al.* (2011)[13]

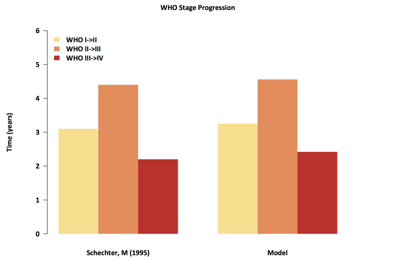

(**D**) Pre-ART WHO stage progression – Schecter *et al.* (1995)[14]

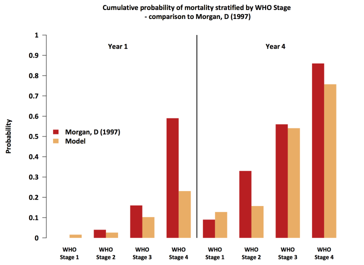

(**E**) Pre-ART cumulative mortality since seroconversion – Morgan *et al.* (1997)[17]

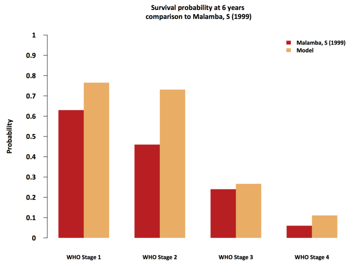

(**F**) Pre-ART survival at 6 years since seroconversion – Malamba *et al.* (1999)[18]

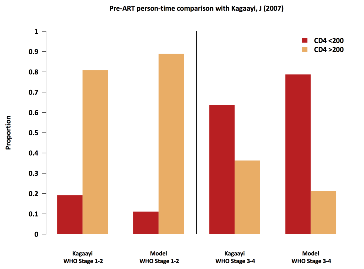

(**G**) Pre-ART person-time spent in each WHO stage, stratified by CD4 category – Kagaayi *et al.* (2007)[19]

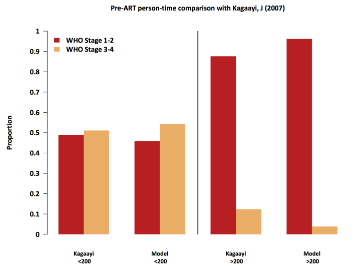

(**H**) Pre-ART person-time spent in each CD4 category, stratified by WHO stage – Kagaayi *et al.* (2007)[19]

Figure S4. Natural history model calibration results compared to original data sources.

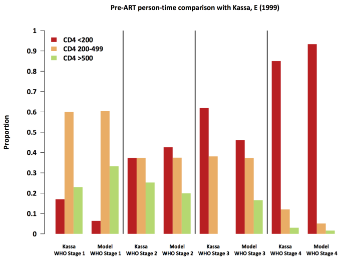

(I) Pre-ART person-time spent in each WHO stage, stratified by CD4 category – Kassa *et al.* (1999)[20]

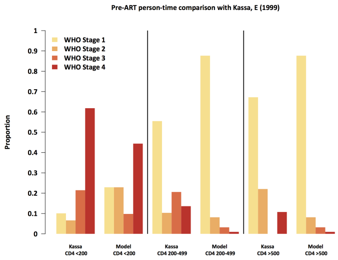

(J) Pre-ART person-time spent in each CD4 category, stratified by WHO stage – Kassa *et al.* (1999)[20]

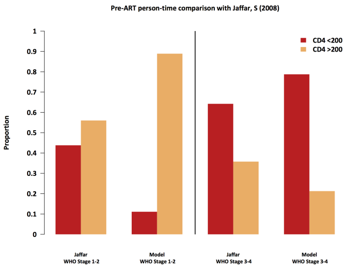

(K) Pre-ART person-time spent in each WHO stage, stratified by CD4 category (< or >200) – Jaffar *et al.* (2008)[21]

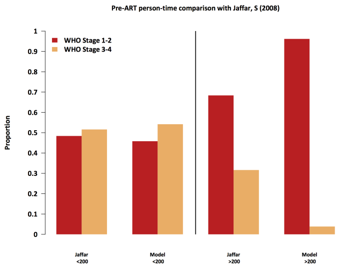

(L) Pre-ART person-time spent in each CD4 category (< or >200), stratified by WHO stage – Jaffar *et al.* (2008)[21]

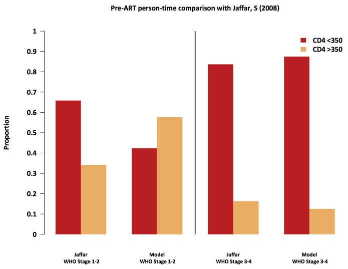

(M) Pre-ART person-time spent in each WHO stage, stratified by CD4 category (< or >350) – Jaffar *et al.* (2008)[21]

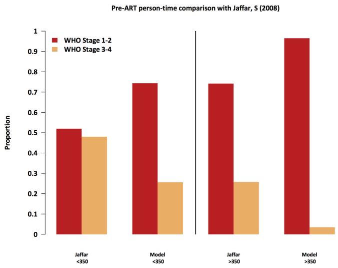

(N) Pre-ART person-time spent in each CD4 category (< or >350), stratified by WHO stage – Jaffar *et al.* (2008)[21]

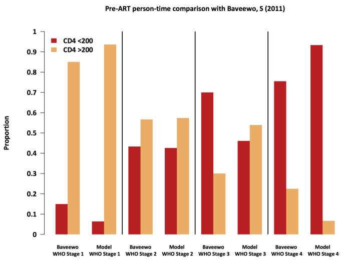

(O) Pre-ART person-time spent in each WHO stage, stratified by CD4 category (< or >200) – Baveewo *et al.* (2011)[22]

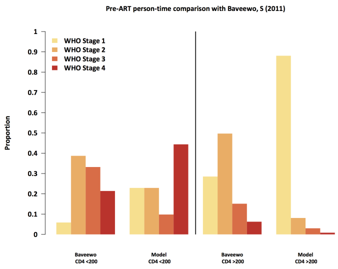

(P) Pre-ART person-time spent in each CD4 category (< or >200), stratified by WHO stage – Baveewo *et al.* (2011)[22]

Figure S4. Natural history model calibration results compared to original data sources (continued).

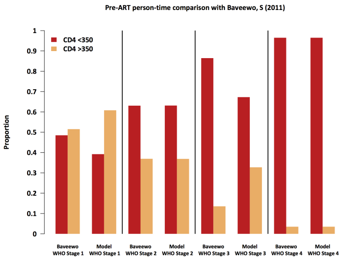

(Q) Pre-ART person-time spent in each WHO stage, stratified by CD4 category (< or >350) – Baveewo *et al.* (2011)[22]

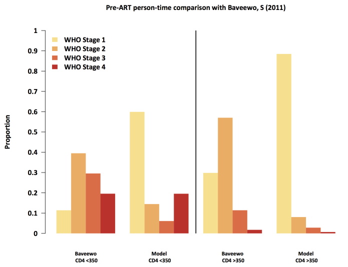

(R) Pre-ART person-time spent in each CD4 category (< or >350), stratified by WHO stage – Baveewo *et al.* (2011)[22]

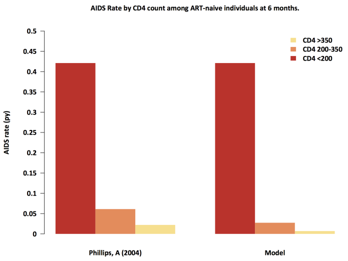

(S) Pre-ART AIDS rate among ART-naive individuals followed for six months – Phillips *et al.* (2004)[15]

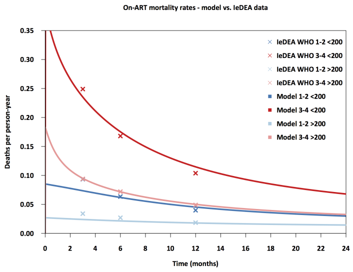

(T) ART mortality rates stratified by WHO stage and CD4 category – May *et al.* (2010)[23]

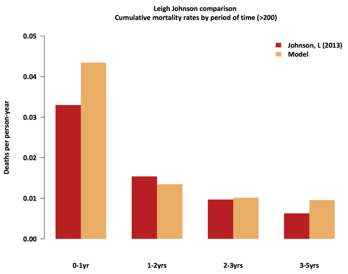

(U) ART cumulative mortality rate, among CD4 >200 – Johnson *et al.* (2013)[24]

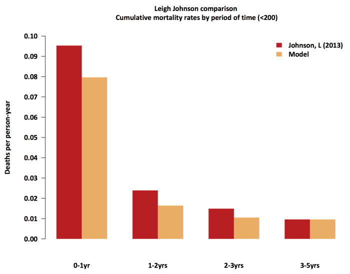

(V) ART cumulative mortality rate, among CD4 <200 – Johnson *et al.* (2013)[24]

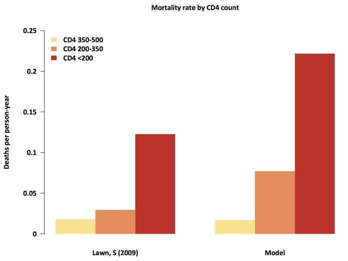

(W) ART mortality rate by CD4 category – Lawn *et al.* (2009)[25]

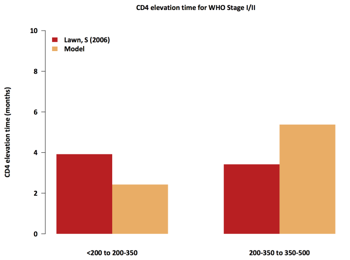

(X) ART CD4 reconstitution time, among WHO stage I & II – Lawn *et al.* (2006)[10]

Figure S4. Natural history model calibration results compared to original data sources (continued).

4 Cascade Model

4.1 Structure

The cascade model describes the events that make up an ART-programme in western Kenya (Fig 1, main text). It captures the possible routes through care and provides insight into how individuals transition between events.

Individuals enter the model as care naive and HIV-negative. The natural history model tracks HIV progression over time and assigns relevant HIV-related mortality rates (Section 3). The rate at which individuals seek care is driven by declining health and care experience, with care seeking behaviour increasing upon the onset of symptoms and also with prior experience of the care system.

With HIV-testing starting in 2004, individuals start to progress through care. Testing can occur through one of three routes: HBCT (home-based counselling and testing) where individuals are sought and tested at home, VCT (voluntary counselling and testing) where individuals voluntarily attend an HIV-clinic, or PICT (provider-initiated counselling and testing) where individuals seek care due to being symptomatic or having had previous healthcare experience. If an individual is found to be HIV-negative, they do not progress any further through care. Individuals may be tested multiple times throughout their lifetime and care will only progress upon a positive diagnosis.

Once identified as HIV-positive, individuals must successfully 'link' to care where they are seen by a clinician and are bled for an initial CD4 count. Linkage may involve travelling to a nearby clinic, and depending upon the route of entry to care, a proportion of tested individuals will be lost prior to this initial CD4 test. Individuals lost from care can re-engage at a later date, either by being picked up through HBCT, voluntarily appearing at a VCT clinic or upon the onset of HIV-related symptoms, seeking care through PICT. For patients that successfully link to pre-ART care, they are seen by a clinician who takes a blood draw for a CD4 count test. These CD4-tests are typically lab-based and have a turnaround time of two weeks.[26] Therefore, patients must return at a later date to receive the results of this test, in which their eligibility for treatment can be determined.

A proportion of individuals are lost between being bled for a CD4 test and returning to receive the results. These individuals, like those who were unsuccessful in linking to care, can re-engage at a later date through being tested via HBCT or VCT, or through PICT. The individuals who were not lost from care after being bled for their CD4 test, return to the clinic to receive their results. However, on the day of the clinic appointment, a small proportion will fail to attend and are lost. Of those that do attend, patients learn of their eligibility for treatment; but, if a patient is ineligible, they must be retained in pre-ART care until they meet the current treatment guidelines.

Pre-ART retention involves returning after one year to receive a secondary CD4 test. During this period a certain proportion of individuals will be lost from care, but can re-engage as described before. Among patients returning for a secondary CD4 test, these individuals will have blood drawn and will again need to return at a later date to receive the results. As before, a certain proportion of patients will not return and will be lost from care. This cycle of pre-ART care continues until the patient becomes eligible for treatment. When this occurs, the patient will be allowed to initiate ART after a small period of time during which they will receive counselling.

Upon initiating ART, the patient will either adhere to treatment and become virally suppressed or fail to adhere and continue to experience declining health. The propensity of a patient to adhere to treatment is an person-specific characteristic. While in ART care, individuals are exposed to a hazard of dropping out, whereby treatment ceases. For adherent individuals, this results in declining health. Yet, as patients failing to adhere to treatment do not receive any health benefits from ART, their health would continue to deteriorate as before. Once lost from ART care in this setting, patients do not naturally seek and return to care.[27]

4.2 Calibration

4.2.1 AMPATH Data

To calibrate the cascade model, we utilised a unique longitudinal dataset from western Kenya. The Academic Model for Providing Access To Healthcare (AMPATH), based in Eldoret, is a collaboration between Moi University, Moi Teaching and Referral Hospital and a consortium of North American academic health centres led by Indiana University working in partnership with the Government of Kenya.[28] AMPATH formed in 2001 with an initial goal to "establish an HIV care system to serve the needs of both urban and rural patients and to assess the barriers to and outcomes of antiretroviral therapy".[29] Since then AMPATH clinics have enrolled >140,000 HIV-infected adults and children across multiple sites in western Kenya.[29]

HIV testing through VCT and PICT, HIV care, and treatment services were established by AMPATH in 2006 in district hospital and health centres.[30] AMPATH launched a pilot HBCT programme in 2010 in Kosirai and Turbo before rolling out HBCT in 2011 to all AMPATH clinic catchment areas ensuring perfect coverage of testing to the community through the Find, Link, Treat, Retain (FLTR) programme (Section 4.2.2).[31,32] All patient visits

from 2004 have been recorded electronically in the AMPATH Medical Record System (AMRS), which furnishes information about patient retention and outcomes.[28,33,34]

The AMRS database used in our analysis contained linked data from VCT, PICT and HBCT rounds from Port Victoria, in Bunyala sub-county (circled in blue on fig S5). This subset described 3,788 HIV-positive individuals tracked over seven years between January 2007 to June 2014. The ability to track the interaction of patients with the care system over time produces a high resolution dataset that can be interrogated to calibrate the model in its entirety. Due to the longitudinal nature of the dataset, answers to each question were stratified into three discrete time periods:

1. **Time split 1** - *(01/01/2007 to 31/12/2009)* - This time period will inform us about the state of care prior to HBCT, when only VCT and PICT were available, and utilises data from the earliest possible point in time.
2. **Time split 2** - *(01/01/2010 to 31/12/2010)* - This time period covers the initial rollout of HBCT in the community prior to the adoption of new treatment guidelines in 2011.
3. **Time split 3** - *(01/01/2011 to 03/06/2014)* - This time period covers the state of care after the adoption of new treatment guidelines in 2011 (CD4 <350 cells/µl or WHO Stage III/IV), together with the full perpetual HBCT rollout as part of the Find, Link, Treat, Retain (FLTR) programme.

The questions asked of the dataset are defined in table S9, and were split into two categories to aid calibration: 'parameters' which were directly input into the model (Tables S10 & S11), or 'calibration points' which were used as targets during the calibration stage.

Two definitions were used to distinguish between a 'gap in care' from 'lost from care'. For pre-ART care, a period of 90 days must elapse after a clinic appointment for an individual to be considered lost. While for ART care, an individual was considered lost if they had a gap in care of more than one year. These definitions allow individuals to be separated into those currently engaged, or disengaged, with care, and to gain insight into the time delay between events in this setting.

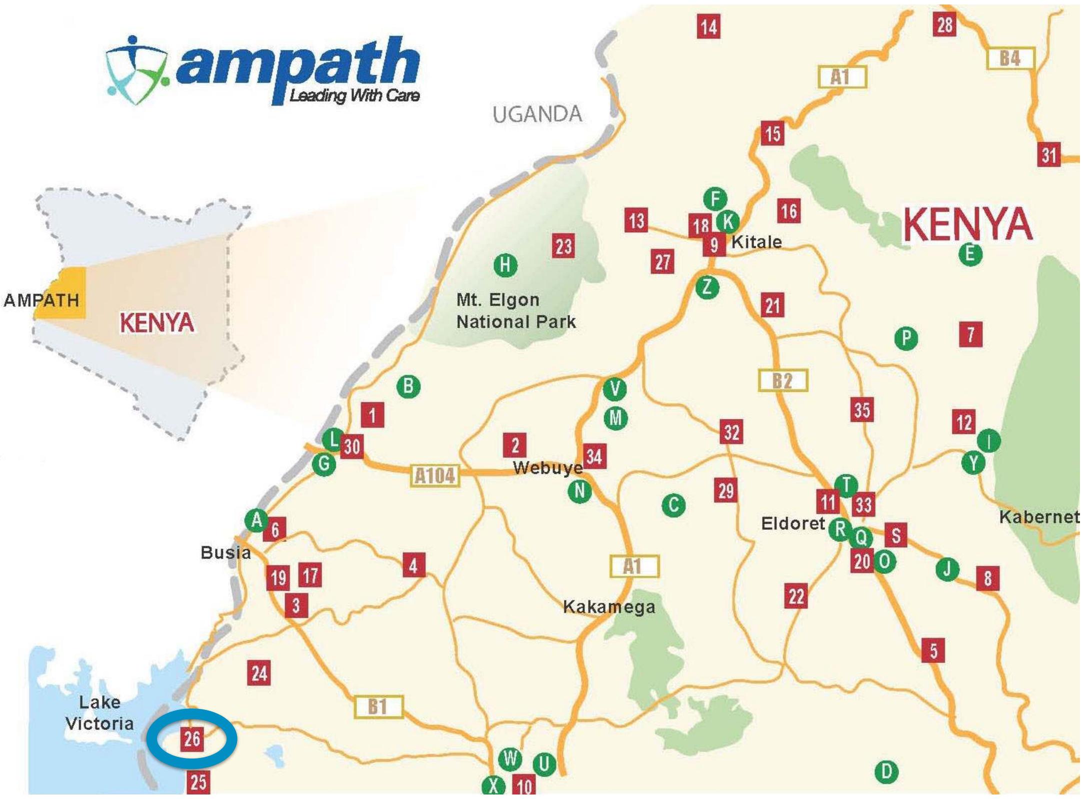

Figure S5. Map of Ministry of Health-AMPATH Clinic Sites in Western Kenya. Port Victoria, Bunyala, is 26 (blue circle). Modified image from: `http://ampath-uoft.ca/about-us/activities/map/`.

Table S9. Questions used to interrogate the AMRS database for calibration of the cascade model.

Question Type	Question	Stratification	Model Use
Testing	The proportion of individuals that enter care through HBCT, VCT and PICT	CD4 count at entry to care	Calibration point
Linkage	The proportion of tested persons who are bled for an initial CD4 test within one month of HIV-test	Route of entry to care	Parameter
	Mean time between HIV-test and initial CD4 test (days)	Route of entry to care, CD4 count at entry to care	Parameter
	Mean CD4 count at first CD4 measurement	Route of entry to care	Calibration point
	Proportion of individuals who received 1st CD4 test returning for test results within one month of test	Route of entry to care, CD4 count at entry to care	Parameter
	Mean time between initial CD4 test and receiving test result (days)	Route of entry to care, CD4 count at entry to care	Parameter
Retention	Mean time between receiving results of CD4 test and being bled for next CD4 test (days)	Route of entry to care	Parameter
	Proportion that ever return for secondary CD4 test prior to ART eligibility	Route of entry to care	Parameter
	Mean number of secondary CD4 test appointments prior to becoming eligible for treatment	Route of entry to care	Calibration point
	Proportion of individuals returning to receive results of secondary CD4 tests	Route of entry to care	Parameter
	Mean time to return to care if lost between CD4 test and receiving CD4 test results (days)	Route of entry to care	Calibration point
	Mean CD4 count when receiving secondary CD4 test results	Route of entry to care	Calibration point
ART Initiation	Mean CD4 count at ART initiation	Route of entry to care	Calibration point
	Mean time to ART initiation from becoming eligible for treatment (days)	Route of entry to care	Parameter
	Mean number of pre-ART clinic visits prior to ART initiation	Route of entry to care	Calibration point
	Proportion of patients initiating ART after diagnosis and successful retention in care until becoming eligible for treatment	Route of entry to care	Calibration point
	Mean time from positive HIV-test to initiating ART for patients diagnosed and successfully retained in care until becoming eligible for treatment (days)	Route of entry to care	Calibration point
	Proportion of patients initiating ART at their enrolment visit	Route of entry to care	Calibration point
	Mean time from HIV-test to initiating ART for patients initiating ART at their enrolment visit (days)	Route of entry to care	Calibration point
	Proportion of patients initiating ART after diagnosis, subsequent loss form pre-ART care (i.e. had at least one CD4 count) but returning prior to becoming eligible for treatment	Route of entry to care	Calibration point
	Mean time to initiating ART after diagnosis, subsequent loss from pre-ART care but returning prior to becoming eligible for treatment (days)	Route of entry to care	Calibration point
	Proportion of patients initiating ART after diagnosis, subsequent loss form pre-ART care and returning when already eligible for treatment	Route of entry to care	Calibration point
	Mean time to initiating ART after diagnosis, subsequent loss from pre-ART care and returning when already eligible for treatment (days)	Route of entry to care	Calibration point
	Proportion of patients initiating ART who had previously been on ART	Route of entry to care	Calibration point
	Dropout rate from ART care ¡1yr after ART initiation (dropout/ppy)	-	Parameter
	Dropout rate from ART care ¿1yr after ART initiation (dropout/ppy)	-	Parameter

Table S10. Summary of parameters used in the cascade model.

Type	Definition			Values	Notes	Reference
HIV-testing	HBCT test time			0.43 years (2.3256py)	In active year, achieves 90% coverage of population.	Estimated for use in intervention version of HBCT
	VCT test time			7.80 years (0.1282py)	Baseline VCT testing rate.	Estimated through calibration to AMPATH dataset
	PICT test time	Asymptomatic	No previous care experience	40 years (0.0250py)	Baseline PICT testing rate given no previous experience of care.	Estimated through calibration to AMPATH dataset
			Never received CD4 result	30 years (0.0334py)	Baseline PICT testing rate given diagnosed but unaware of CD4 count.	Estimated through calibration to AMPATH dataset
			Ever received CD4 result	10 years (0.1000py)	Baseline PICT testing rate given diagnosed and aware of CD4 count.	Estimated through calibration to AMPATH dataset
		Symptomatic	No previous care experience	1.525 years (0.6557py)	Baseline PICT testing rate given symptomatic but undiagnosed.	Estimated through calibration to AMPATH dataset
			Diagnosed	0.1 years (10py)	Baseline PICT testing rate given symptomatic and diagnosed.	Estimated through calibration to AMPATH dataset
Linkage	HBCT linkage probability	In roll-out year (2010)		5.4%	Probability of linkage after HBCT in roll-out year.	Value derived from AMPATH dataset
		Subsequent years	Newly diagnosed	30%	Probability of linkage after HBCT given previously undiagnosed.	Estimated through calibration to AMPATH dataset
			Previously diagnosed	40%	Probability of linkage after HBCT given previously aware of serostatus.	Estimated through calibration to AMPATH dataset
	HBCT time delay between HIV-test and initial clinic visit		CD4 >500	93 days	Time to attend clinic with CD4 >500.	Value derived from AMPATH dataset
			CD4 350-500	97 days	Time to attend clinic with CD4 350-500.	Value derived from AMPATH dataset
			CD4 200-350	152.5 days	Time to attend clinic with CD4 200-350.	Value derived from AMPATH dataset
			CD4 <200	142 days	Time to attend clinic with CD4 <200.	Value derived from AMPATH dataset
	VCT linkage probability			95%	Probability of linkage given tested through VCT.	Estimated through calibration to AMPATH dataset
	PICT linkage probability			70%	Probability of linkage given tested through PICT.	Estimated through calibration to AMPATH dataset

Table S11. Summary of parameters used in the cascade model (continued).

Type	Definition		Values	Notes	Reference
Pre-ART Care	Time between CD4 test and result appointment		30 days	-	Valued derived from AMPATH dataset
	Time between receiving results of CD4 test and being bled for next CD4 test		279 days	-	Valued derived from AMPATH dataset
	On day of CD4 test result appointment, probability of attending		94.89%	-	Estimated through calibration to AMPATH dataset
	Pre-ART retention probability (probability of not being lost from care after CD4 test)	HBCT	94.89%	Risk of not being lost from care after CD4 test given route into care was through HBCT.	Estimated through calibration to AMPATH dataset
		VCT	94.89%	Risk of not being lost from care after CD4 test given route into care was through VCT.	Estimated through calibration to AMPATH dataset
		PICT	94.89%	Risk of not being lost from care after CD4 test given route into care was through PICT.	Estimated through calibration to AMPATH dataset
	After receiving CD4 test result, probability of returning for secondary CD4 test	HBCT	75%	Risk of attending secondary CD4 test if route of entry to care was through HBCT.	Estimated through calibration to AMPATH dataset
		VCT	75%	Risk of attending secondary CD4 test if route of entry to care was through VCT.	Estimated through calibration to AMPATH dataset
		PICT	75%	Risk of attending secondary CD4 test if route of entry to care was through PICT.	Estimated through calibration to AMPATH dataset
ART Care	Time to ART initiation from receiving CD4 test result confirming eligibility for treatment		145.12 days	-	Value derived from AMPATH dataset
	Proportion of individuals initiating ART who adhere to ART and achieve viral suppression		86%	Value caculated from average viral suppression of individuals on ART from 35 countries by Boender *et al.* (2015). Mean proportion suppressed between 6 and 60 months after ART initiation taken from supplementary material.	Value derived from Boender *et al.* (2015).[35]
	ART dropout time	<1 year since ART initiation	21.72 years (0.045py)	Risk of being lost from ART care <1 year after initiation.	Value derived from AMPATH dataset
		>1 year since ART initiation	52.13 years (0.019py)	Risk of being lost from ART care >1 year after initiation.	Value derived from AMPATH dataset

4.2.2 HBCT

AMPATH began trialling HBCT in Port Victoria, Bunyala, from 2010 onwards, with the programme achieving >85% coverage of the community initially, but perfect coverage has since been achieved through 'perpetual' HBCT as part of the FLTR programme.[31,36] However, early HBCT rounds only involved passive referral of infected patients, but AMPATH HBCT campaigns now include active follow-up of patients, and linkage rates are expected to be considerably higher.[31] As our goal was to describe the impact of various interventions on patient outcomes relative to a 'status quo' ART programme similar in structure to AMPATH before the introduction of HBCT, we created two versions of HBCT:

1. **Calibration HBCT** – used during the model calibration to AMPATH data, simulating perpetual HBCT with 100% coverage every year from 2010 (as seen in AMPATH sites). Additionally, we assume that linkage was poor in the first year as the intervention was being rolled out.

2. **Intervention HBCT** – used for assessing the impact of a hypothetical HBCT intervention. Relies on different assumptions: 90% coverage of the population every 4 years (Table 2 & Section 7).

The "HBCT test time" in table S10 refers to the inverse of the testing rate for the intervention version of HBCT. Additionally, we make the assumption that during HBCT testing, if a patient has been previously diagnosed, they are more likely to link to care then a patient who was previously unaware of their infection (Table S10).

4.2.3 Results to AMPATH Data

Due to the complex nature of the model and the range of data available from AMPATH for calibration, the cascade model was calibrated by hand. With parameters from AMPATH entered into the model (Table S10), calibration was conducted systematically and chronologically.

Starting with data defining the proportion of persons living with HIV (PLHIV) who were were aware of their infection at the beginning of 2010, 62% of HIV-positive individuals were diagnosed, with 2/3 diagnosed through VCT services and the remaining 1/3 through PICT. By adjusting the baseline rate of seeking care through VCT ('VCT test time', Table S10) and the health care seeking rates driving individuals to seek care through PICT ('PICT test time', Table S10), we matched these values exactly as shown in fig S6.

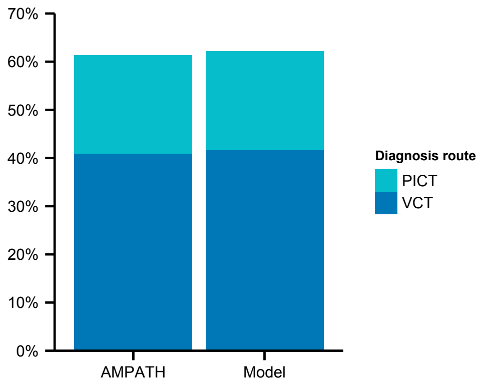

Figure S6. Proportion of infected individuals aware of HIV-status by 2010.

With knowledge of AMPATH's HBCT programme rollout in 2010, followed by the addition of the FLTR programme where HBCT became "perpetual" (home-team does not leave an area until everyone is tested, resulting in 100% coverage), we looked at the proportion of individuals entering care through each of the three routes (HBCT/VCT/PICT) over the three time splits and calibrated model output to AMPATH data (Fig S7). During calibration, we used this 'calibration version' of HBCT (Section 4.2.2), with perfect coverage, and adjusted the rates of linkage to care from VCT and PICT, before adjusting the linkage rates following HBCT in both the 'roll-out period' (2010 to 2011) and thereafter (See 'Linkage', Table S10).

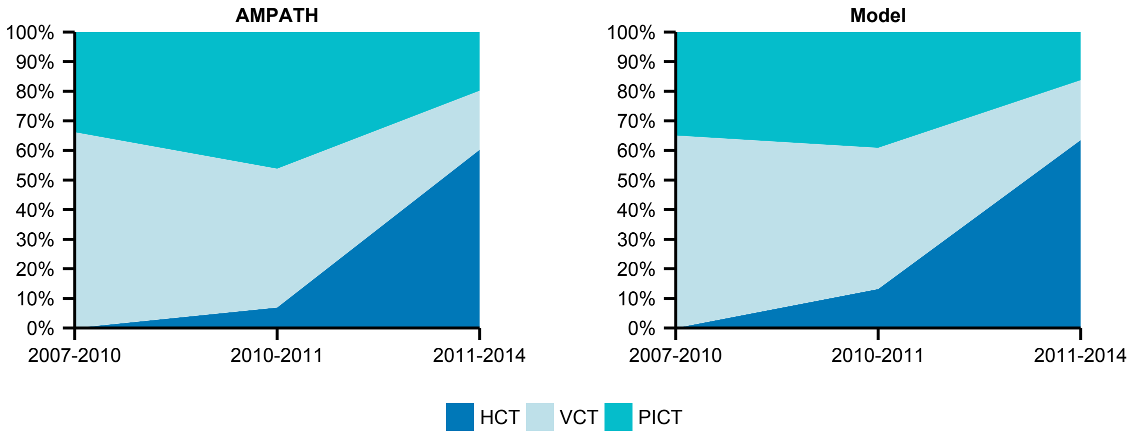

Figure S7. Distribution of individuals across the three routes of entry into care.

The CD4 distribution of individuals entering care was then calibrated to data from AMPATH. Fig S8, illustrates the calibration results; however, it can be seen that the proportion of individuals entering care through VCT and PICT with CD4 <200 in the data, show an uptick in the period 2011 to 2014.

The reason for this uptick in the data is unknown, and is not captured by the model. The data was adjusted for individuals without a CD4 count on record at entry to care, and also for individuals with no route entry recorded.

Here, we assume that all patients without a CD4 count had a CD4 of <200, and among those who had no route of entry recorded, 1/3 entered through PICT care and 2/3 through VCT services.

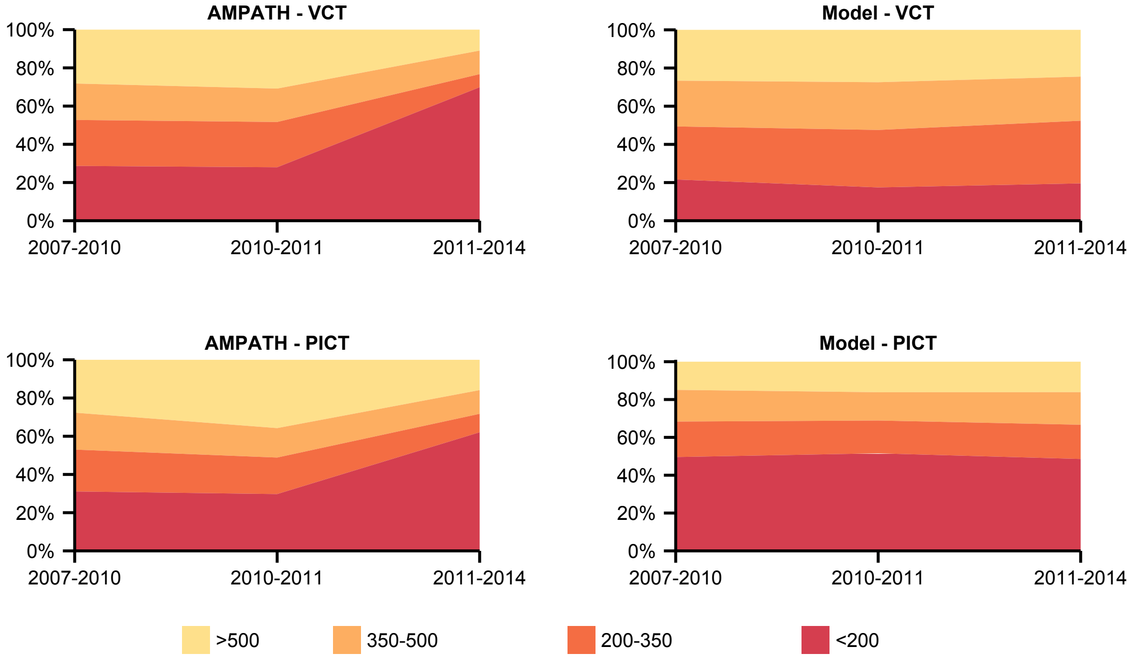

Figure S8. CD4 distribution at entry to care.

The CD4 distribution among individuals initiating ART in the model was then calibrated to AMPATH data. This distribution is altered by changes in treatment guidelines, care seeking rates and the natural history of HIV. As can be seen in fig S9, many individuals initiate ART in AMPATH with very high CD4 counts (higher than treatment guidelines); this is due to a high prevalence of opportunistic infections (OI's) in Bunyala. To reconcile the model with the data, we allow all patients with WHO stage III or IV infections to initiate treatment immediately upon presentation to care. We also allow a random 5% of patients presenting to initiate immediately, to capture additional OI's that are not defined as WHO stage infections and to match the CD4 distribution at ART initiation seen in the AMPATH data.

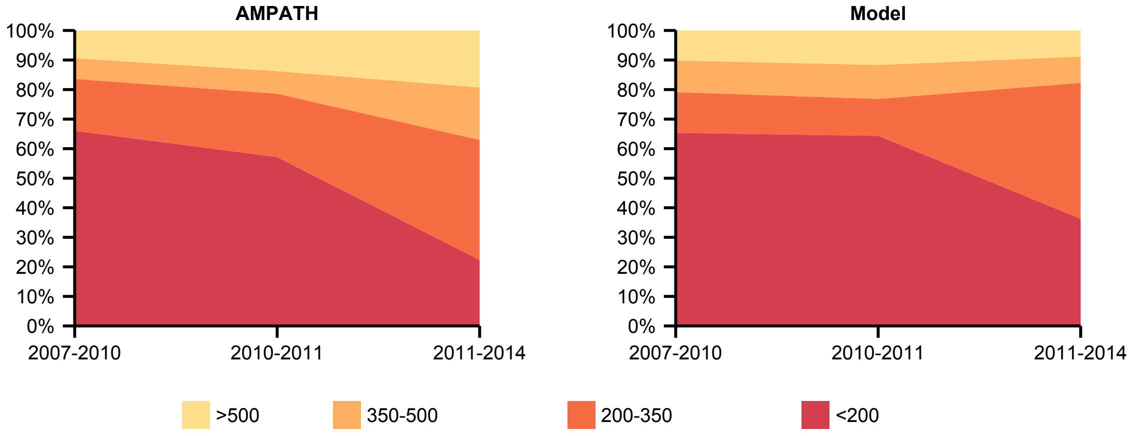

Figure S9. CD4 distribution at ART initiation.

We then calibrated the model to data on the prior experience of care among patients initiating ART (Fig S11).

We noticed an incompatibility between the data and the model during this stage of calibration. We see that in the data, the majority of individuals initiate ART following enrollment into care, but not at enrollment. In contrast, in the model, we see that the majority of individuals initiate treatment at enrollment.

In this calibration, the model fails to capture this aspect of care seen in the AMPATH data, as we assume that there is a strong tendency to seekcare when symptomatic. This process has been previously termed as “reaching ART via the ‘side-door’ ”, as patients enter care and initiate treatment immediately, bypassing the other stages of pre-ART care.[37] To reconcile this, we produced alternative calibrations that we use to explore how different intepretations of the available AMPATH data affects results (Section 4.2.5 & 4.2.7).

Finally, the proportion of HIV-positive individuals on ART in mid-2010 and the proportion of patients in care, on ART at the end of May 2014 in the model was calibrated to data from AMPATH (Fig S10). The model fails to capture the high proportion of patients in care and on ART in 2014, it is thought that this is may be due to patients being initiated early onto treatment before they become eligible. All parameter values from this calibration can be found in table S10.

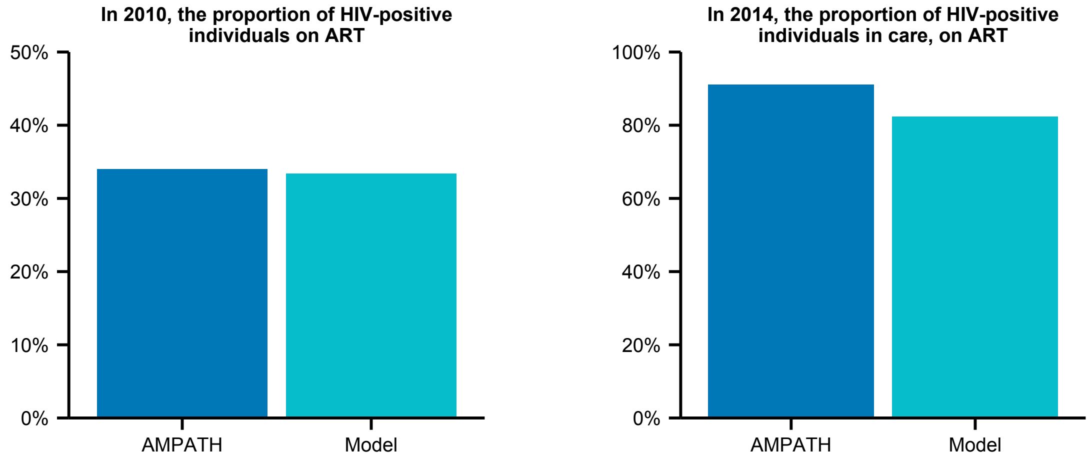

Figure S10. Proportion of individuals initiating ART per year.

(**A**) Among individuals initiating ART between 2007 and 2010.

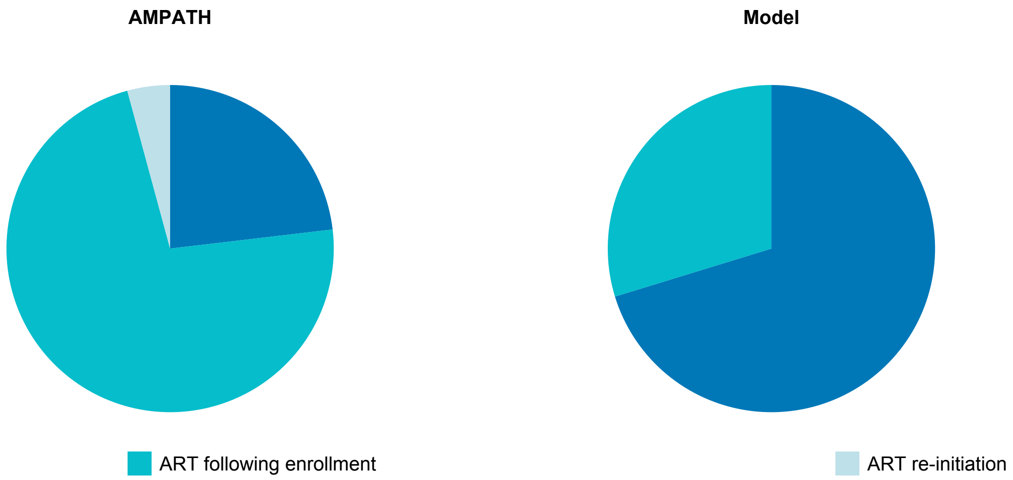

(**B**) Among individuals initiating ART between 2010 and 2011.

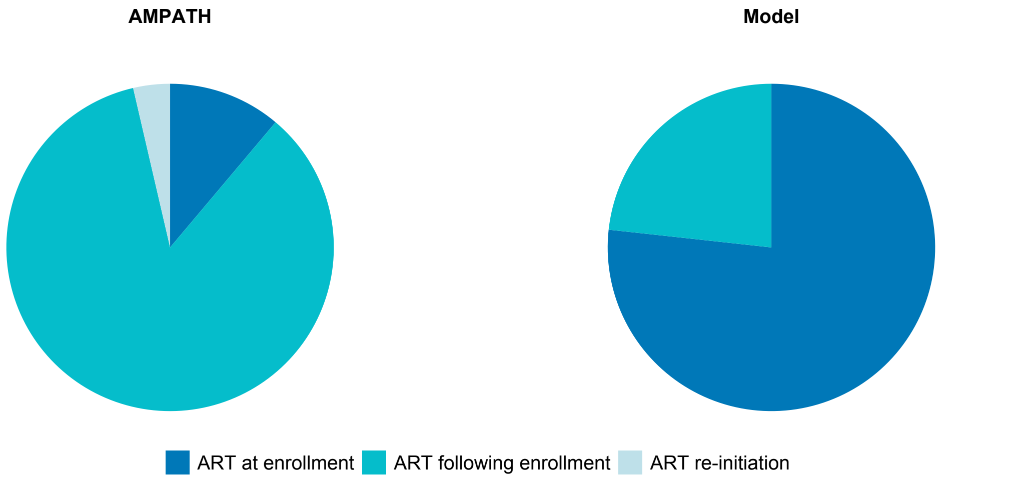

(**C**) Among individuals initiating ART between 2011 and 2014.

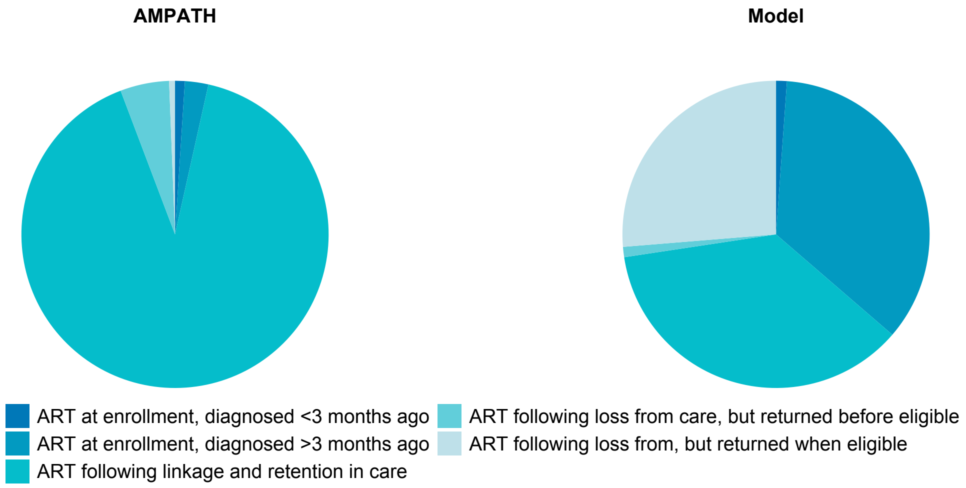

Figure S11. Prior care experience among individuals initiating ART.

4.2.4 Results to Province-Level Estimates

After calibration to data from AMPATH, we independently compared the model to estimates of HIV prevalence from the 2012 Kenya AIDS Indicator Survey at the province-level, before comparing the model to prevalence estimates in Bunyala from AMPATH, and comparing the model to national estimates from UNAIDS. With the calibration version of HBCT implemented from 2010 (Section 4.2.2), we compared HIV prevalence in the model to estimates from the 2012 Kenya AIDS Indicator Survey from Western Province (Fig S12).[38] As we are aiming to describe improvements to care in Kenya through extrapolating results from data in Bunyala, a close fit to prevalence estimates from Western Province (which contains Bunyala) is desirable.

We also compared prevalence in the model to the latest estimates from AMPATH in Bunyala (Fig S13). We fail to capture the same prevalence distribution in Bunyala for two reasons: firstly, incident infections in the model only occur among individuals aged 15 years or older as incidence is primarily driven by estimates from Spectrum (Table S4),[2] and secondly, the prevalence of HIV in Bunyala is higher than sub-national estimates. This is likely due to the location of Port Victoria in Bunyala, on the shores of Lake Victoria where the "sex for fish" trade contributes to incidence.[39] Because the AMPATH prevalence estimates indicate a higher prevalence than the KAIS estimates, we chose to make the model best match a compromise in prevalence between Western Kenya (KAIS) and AMPATH.

We also compared the model to national estimates of prevalence, AIDS-related death estimates and ART coverage to data from UNAIDS (Fig S14). The model fails to capture the decline seen in HIV-prevalence estimates from UNAIDS; however, the UNAIDS figures are likely to be indirect estimates from measuring prevalence among pregnant women attending antinatal clinics. This method of estimation has recently been shown to underestimate the trend in prevalence among the general population.[40] Additionally, the model does not match national trends in ART coverage. This is due to the presence of HBCT in Bunyala and the efficiency of the AMPATH care system.

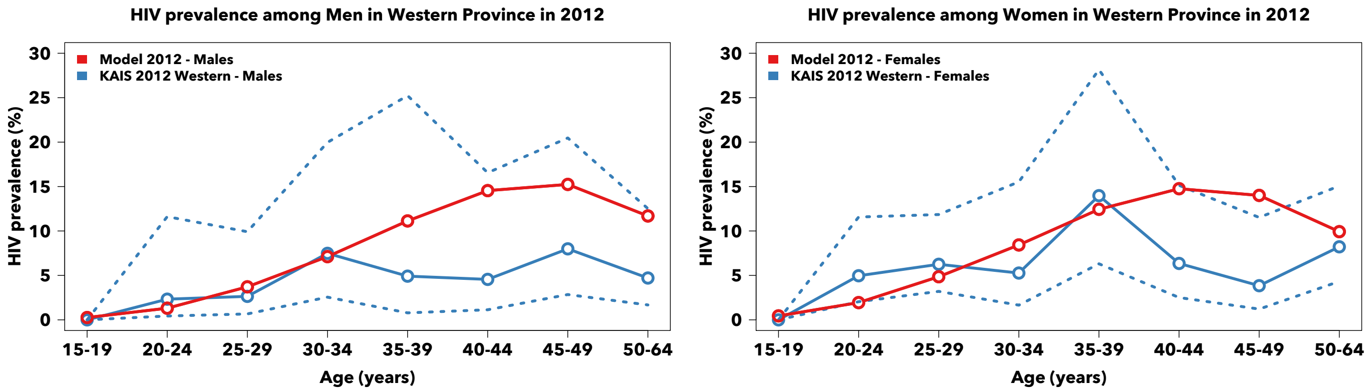

Figure S12. Comparison of HIV-prevalence in the model to KAIS 2012 estimates from Western Province.

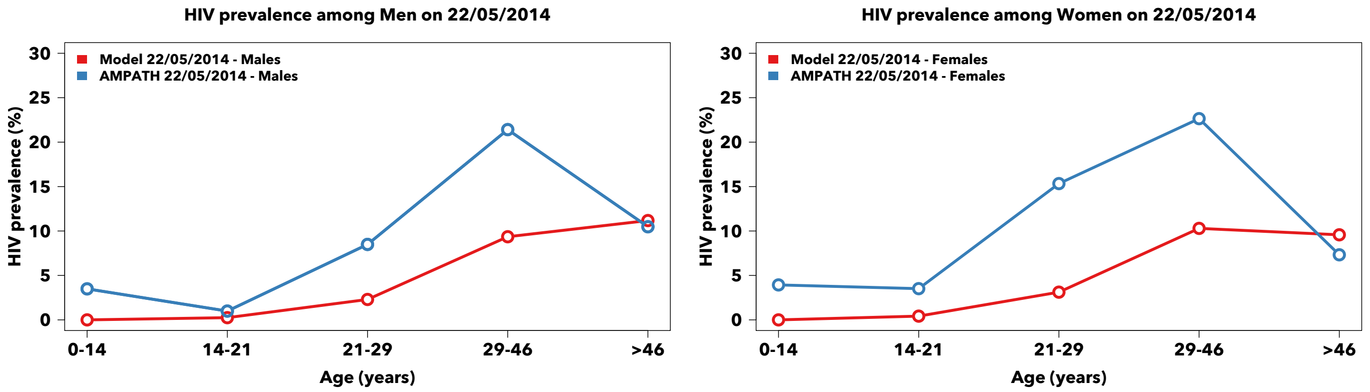

Figure S13. Comparison of HIV-prevalence in the model to AMPATH estimates from Bunyala in 2014.

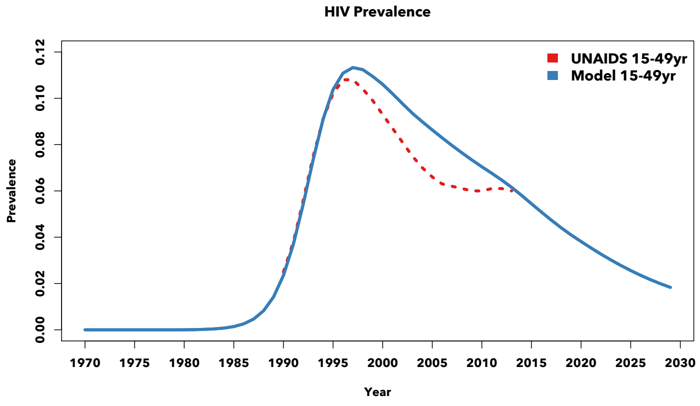

(A) Historical HIV-prevalence trend.

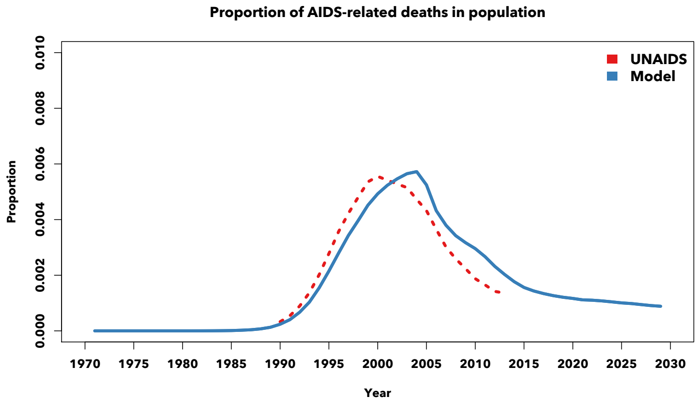

(B) AIDS deaths as a proportion of the total population.

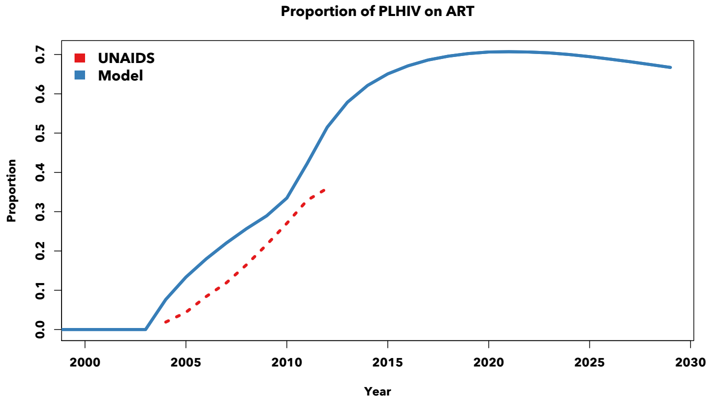

(C) ART coverage.

Figure S14. Comparison to UNAIDS estimates from Spectrum.[2]

4.2.5 Uniform Care Seeking Calibration: Results to AMPATH Data

This "Uniform Care Seeking" calibration aimed to provide a better model fit to the distribution of prior care experience among patients initiating ART. This is in response to the incompatibility between some of the AMPATH data and the model seen in the standard calibration (Section 4.2.3).

The standard calibration includes a strong tendency for individuals to seek care when they are symptomatic (a strong 'side-door').[37] To reconcile the issue seen in the standard calibration, this "Uniform Care Seeking" calibration makes the assumption that care seeking is stronger and more uniform, with patients not seeking care faster when symptomatic. Additionally, we also assume that the rate of care seeking behaviour is also not a function of previous care experience. The care seeking behaviour parameters for the standard calibration are shown in table S10, and the updated parameter values for this calibration are shown in table S12.

Table S12. Summary of updated parameters used in the Uniform Care Seeking calibration.

**Definition**			**Values**	**Notes**
VCT test time			5.50 years (0.1819py)	Baseline VCT testing rate.
PICT test time	Asymptomatic	No previous care experience	8 years (0.1250py)	Baseline PICT testing rate given no previous experience of care.
		Never received CD4 result	8 years (0.1250py)	Baseline PICT testing rate given diagnosed but unaware of CD4 count.
		Ever received CD4 result	8 years (0.1250py)	Baseline PICT testing rate given diagnosed and aware of CD4 count.
	Symptomatic	No previous care experience	8 years (0.1250py)	Baseline PICT testing rate given symptomatic but undiagnosed.
		Diagnosed	8 years (0.1250py)	Baseline PICT testing rate given symptomatic and diagnosed.
On day of CD4 test result appointment, probability of attending			98.99%	-
Pre-ART retention probability (probability of not being lost from care after CD4 test)		HBCT	98.99%	Risk of not being lost from care after CD4 test given route into care was through HBCT.
		VCT	98.99%	Risk of not being lost from care after CD4 test given route into care was through VCT.
		PICT	98.99%	Risk of not being lost from care after CD4 test given route into care was through PICT.
After receiving CD4 test result, probability of returning for secondary CD4 test		HBCT	98%	Risk of attending secondary CD4 test if route of entry to care was through HBCT.
		VCT	98%	Risk of attending secondary CD4 test if route of entry to care was through VCT.
		PICT	98%	Risk of attending secondary CD4 test if route of entry to care was through PICT.

Below are the "Uniform Care Seeking" calibration results with no increase in care seeking behaviour for symptomatic patients. In order to produce the same fraction of individuals on ART in 2010 and 2014 (Fig S20), the baseline rates of care-seeking behaviour were increased to compensate for the lack of faster care-seeking among sick individuals (Table S12). As a result, the fraction of PLHIV diagnosed by 2010 increases above the estimates provided by AMPATH (Fig S15).

Additionally, the model fit to the distribution of individuals at entry to care by route of entry is weaker than in the standard calibration (Fig S16).

A by-product of increasing baseline rates of care-seeking behaviour in this alternate calibration is that the CD4 distribution of individuals entering care in the model is a better fit to the AMPATH data than in the standard calibration, particularly with respect to the proportion of individuals with CD4 >500 entering care (Fig S17 vs. Fig S8).

However, the CD4 distribution of patients at ART initiation is lower because fewer individuals are allowed to initiate ART at enrolment (Fig S18). In this calibration, only patients with WHO stage IV infections and a random 2% of patients presenting to care were allowed to initiate treatment immediately. This restriction was implemented due to the need for this calibration to be aligned with the distribution of prior care experience among patients initiating ART (Fig S19). Additionally, rates of retention in pre-ART care were marginally increased to

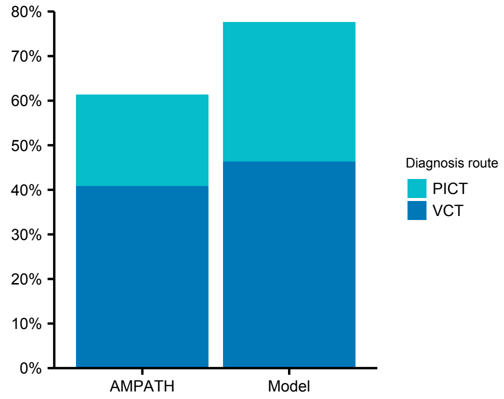

Figure S15. Uniform Care Seeking Calibration - Proportion of infected individuals aware of HIV-status by 2010.

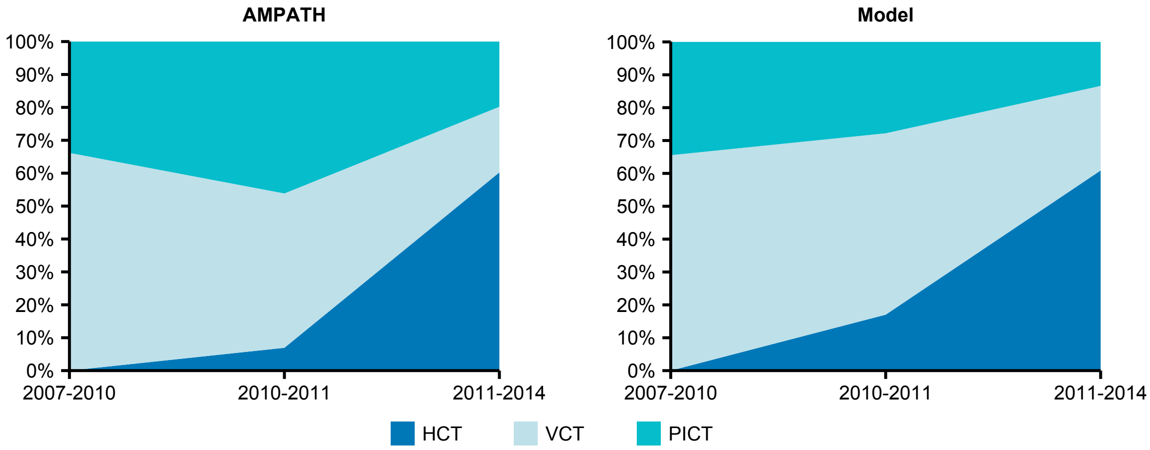

Figure S16. Uniform Care Seeking Calibration - Distribution of individuals across the three routes of entry into care.

achieve a better model fit (Table S12).

Additionally, this calibration needed to produce the same number of individuals on ART in 2010 and 2014 as in the standard calibration. This was achieved through increasing the baseline rates of seeking care and allows the model to match AMPATH data in 2010, but falls short of matching AMPATH data in 2014 (Fig S20).

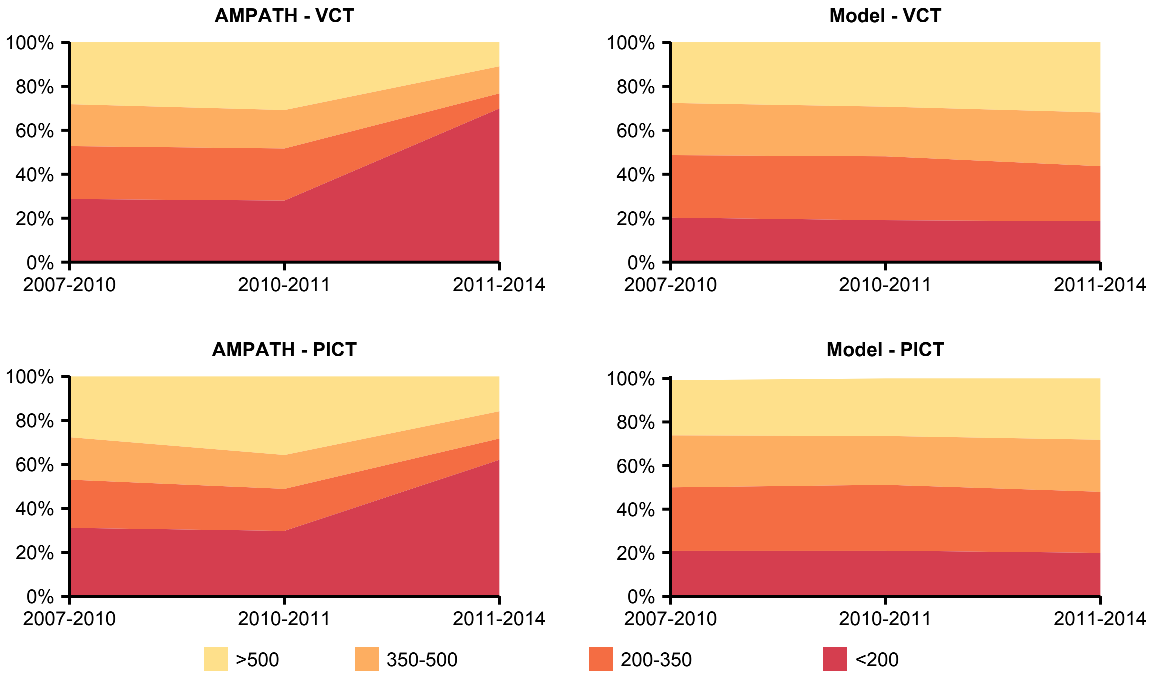

Figure S17. Uniform Care Seeking Calibration - CD4 distribution at entry to care.

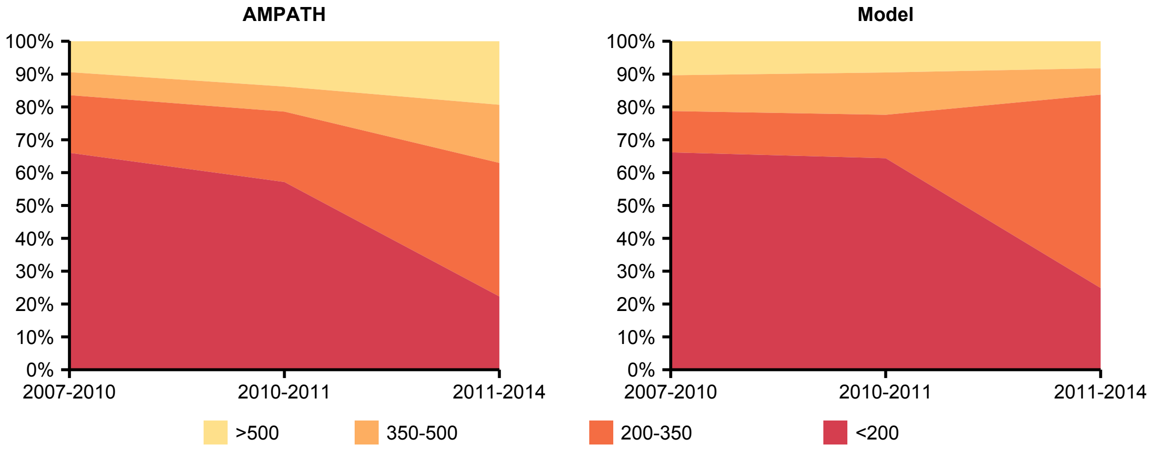

Figure S18. Uniform Care Seeking Calibration - CD4 distribution at ART initiation.

(**A**) Among individuals initiating ART between 2007 and 2010.

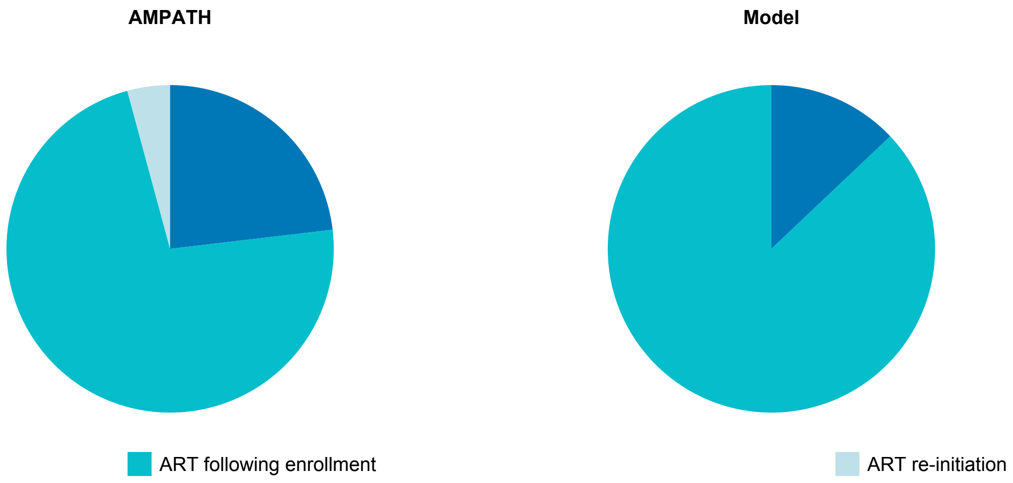

(**B**) Among individuals initiating ART between 2010 and 2011.

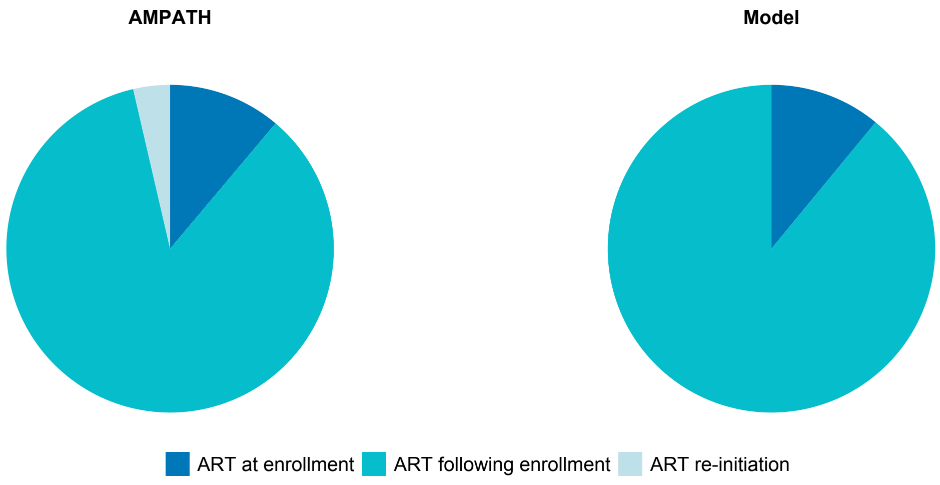

(**C**) Among individuals initiating ART between 2011 and 2014.

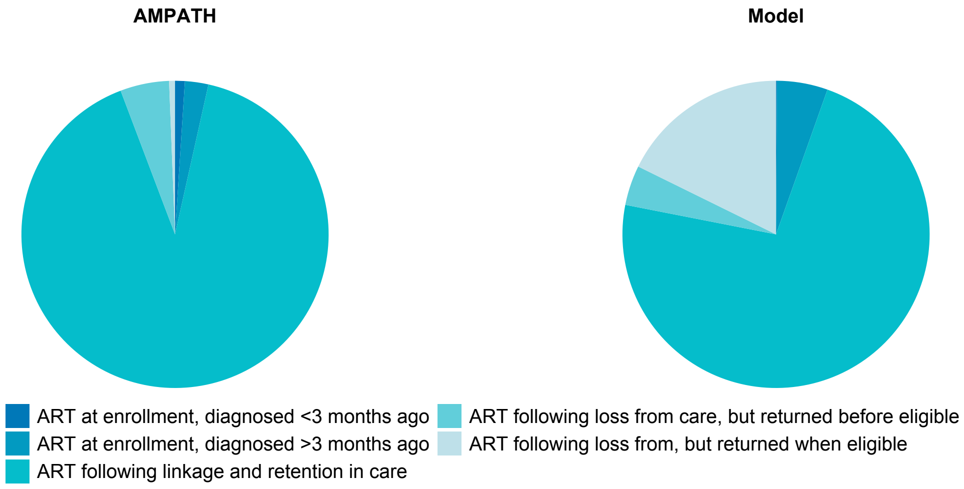

Figure S19. Uniform Care Seeking Calibration - Prior care experience among individuals initiating ART.

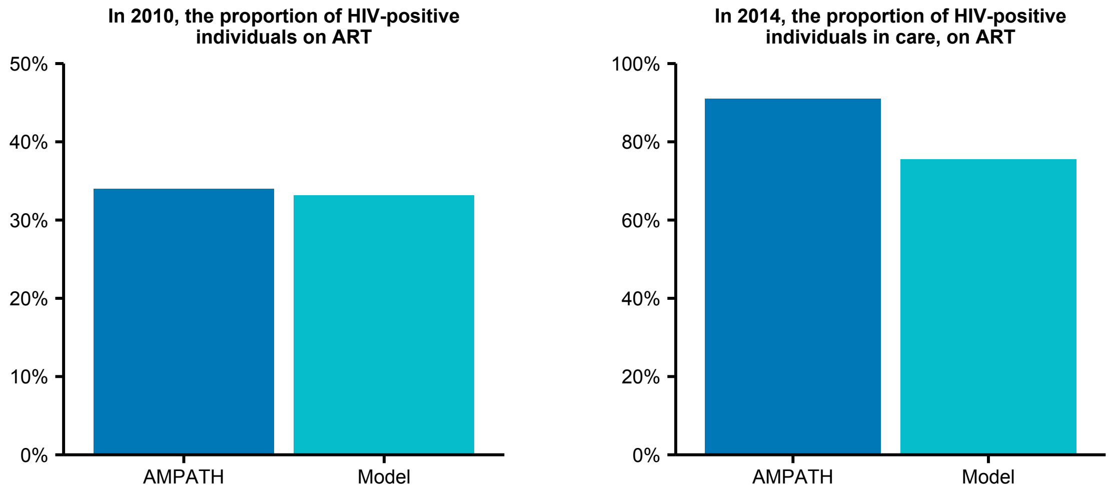

Figure S20. Uniform Care Seeking Calibration - Proportion of individuals initiating ART per year.

4.2.6 Uniform Care Seeking Calibration: Results to Province-Level Estimates

After calibration to data from AMPATH, we independently compared this "Uniform Care Seeking" version of the model to estimates of HIV prevalence from the 2012 Kenya AIDS Indicator Surveys at the province level, prevalence estimates from AMPATH, and to national UNAIDS estimates. With the 'calibration version' of HBCT implemented from 2010 (Section 4.2.2), we compared HIV prevalence in the model to estimates from the 2012 Kenya AIDS Indicator Survey (Fig S21),[38] estimates from AMPATH (Fig S22), and to national estimates of prevalence, AIDS-related death estimates and ART coverage to data from UNAIDS (Fig S23).

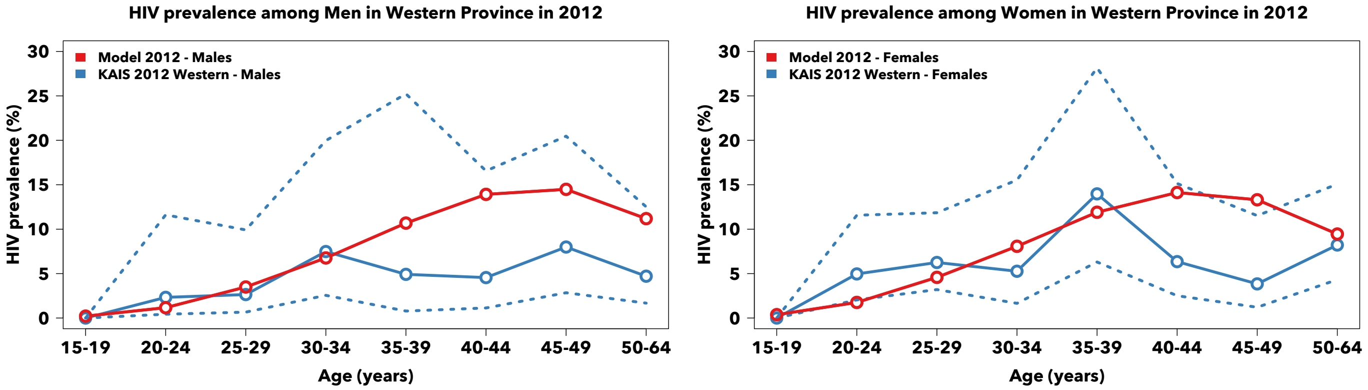

Figure S21. Uniform Care Seeking Calibration - Comparison of HIV-prevalence in the model to KAIS 2012 estimates from Western Province.

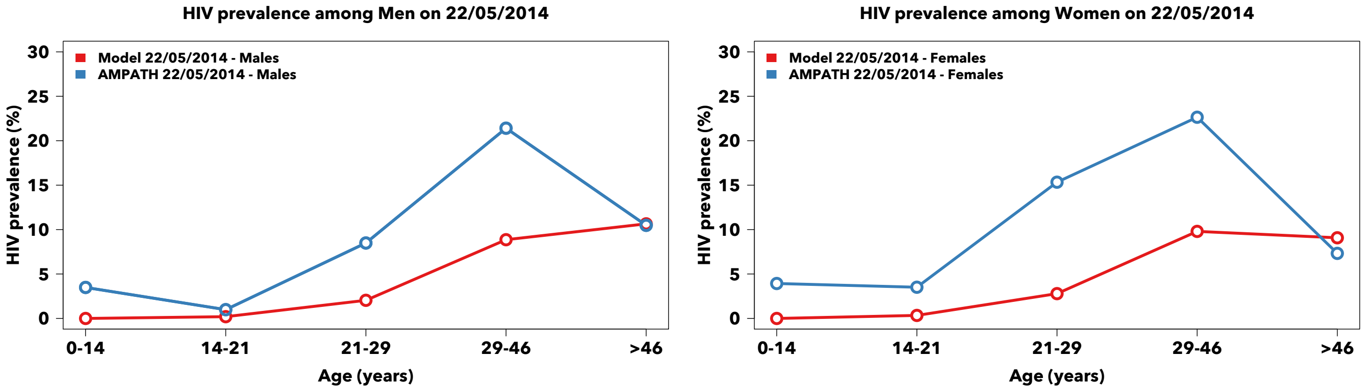

Figure S22. Uniform Care Seeking Calibration - Comparison of HIV-prevalence in the model to AMPATH estimates from Bunyala in 2014.

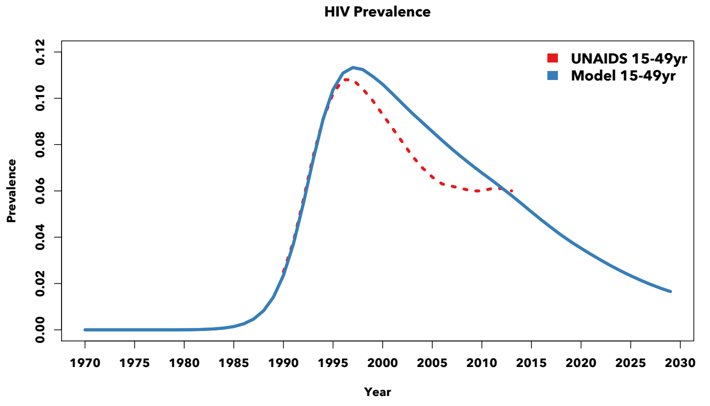

(A) Historical HIV-prevalence trend.

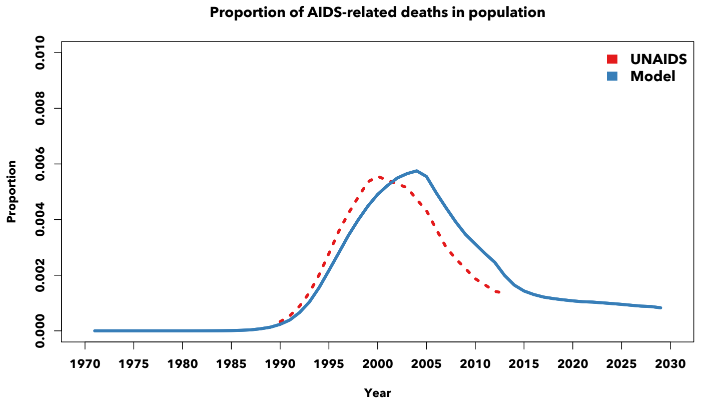

(B) AIDS deaths as a proportion of the total population.

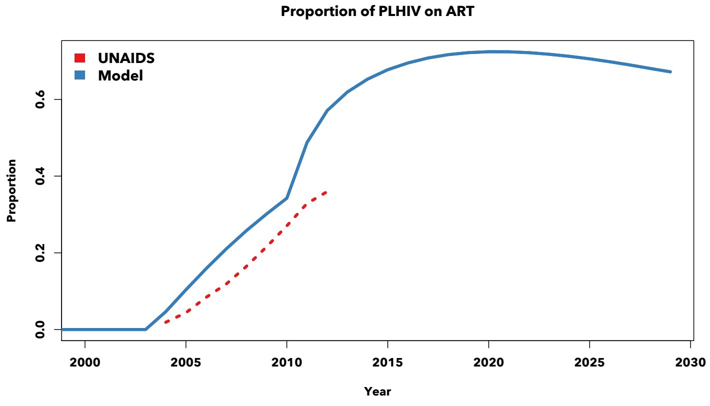

(C) ART coverage.

Figure S23. Uniform Care Seeking Calibration - Comparison to UNAIDS estimates from Spectrum.[2]

4.2.7 Disease-Led Care Seeking Calibration: Results to AMPATH Data

A second alternative calibration of the model, the "Disease-Led Care Seeking" calibration was created to illustrate the impact of a third set of assumptions around care seeking behaviour. In the standard calibration (Section 4.2.3), individuals have a strong tendency to seek care when they are symptomatic and additionally seek care at a higher rate after previous care experience. However, the "Uniform Care Seeking" calibration (Section 4.2.5), illustrates how removing this strong tendency to seek care when symptomatic or with previous care experience impacts results.

Now, this third "Disease-Led Care Seeking" calibration aims to illustrate the impact of only allowing the presence of symptoms to increase care seeking behaviour. That is, having previous experience of care does not increase the tendency of an individual to seek further care. By presenting the results of three different calibrations to the data, we show how various assumptions can be made around health care seeking behaviour in the model, but that the primary outcomes of this paper remain the same. The care seeking behaviour parameters for the standard calibration are shown in table S10, and the updated parameter values for this calibration are shown in table S13.

Table S13. Summary of updated parameters used in the Disease-Led Care Seeking calibration.

Definition			Values	Notes
VCT test time			8 years (0.1250py)	Baseline VCT testing rate.
PICT test time	Asymptomatic	No previous care experience	25 years (0.0400py)	Baseline PICT testing rate given no previous experience of care.
		Never received CD4 result	25 years (0.0400py)	Baseline PICT testing rate given diagnosed but unaware of CD4 count.
		Ever received CD4 result	25 years (0.0400py)	Baseline PICT testing rate given diagnosed and aware of CD4 count.
	Symptomatic	No previous care experience	1 years (1.0000py)	Baseline PICT testing rate given symptomatic but undiagnosed.
		Diagnosed	1 years (1.0000py)	Baseline PICT testing rate given symptomatic and diagnosed.

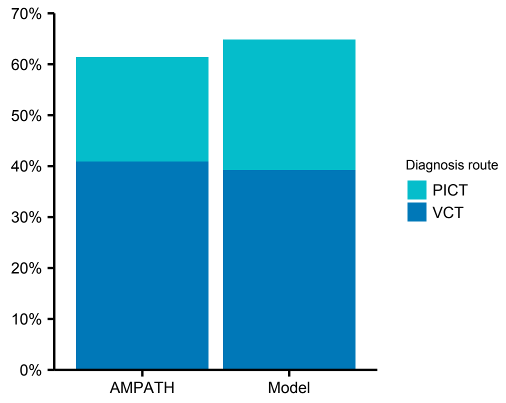

Figure S24. Disease-Led Care Seeking Calibration - Proportion of infected individuals aware of HIV-status by 2010.

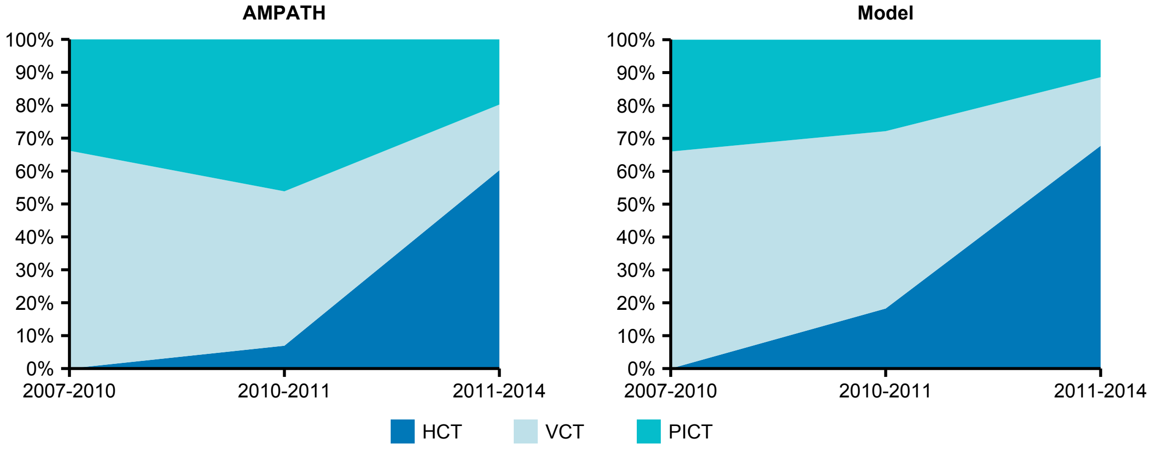

Figure S25. Disease-Led Care Seeking Calibration - Distribution of individuals across the three routes of entry into care.

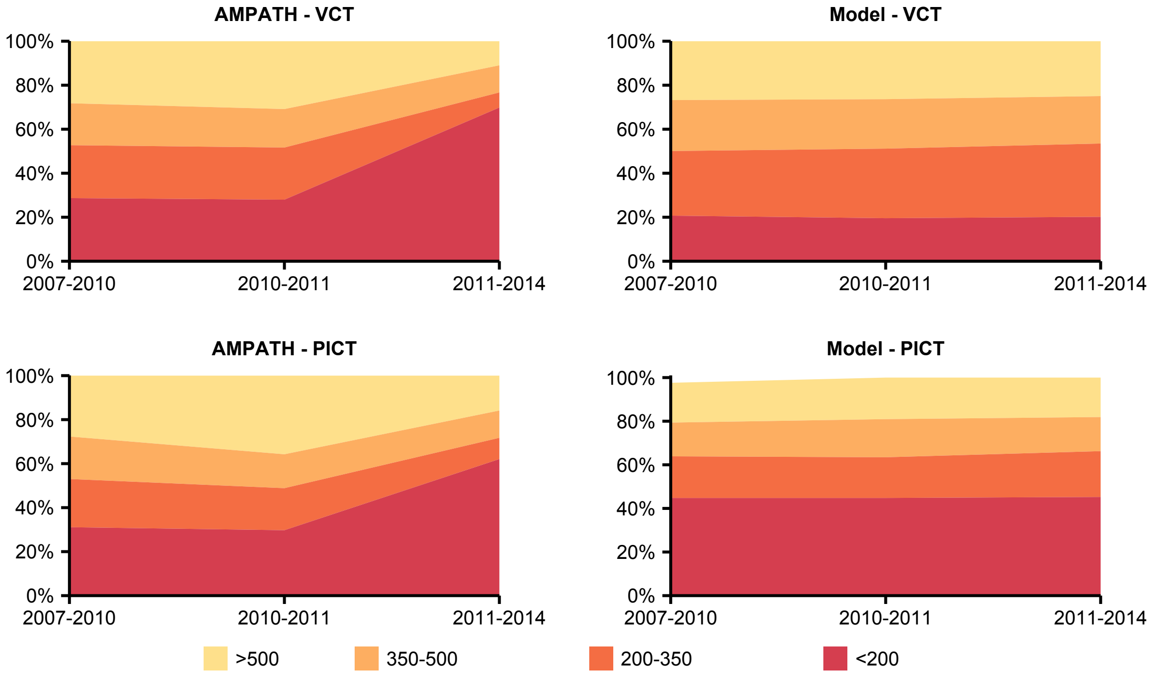

Figure S26. Disease-Led Care Seeking Calibration - CD4 distribution at entry to care.

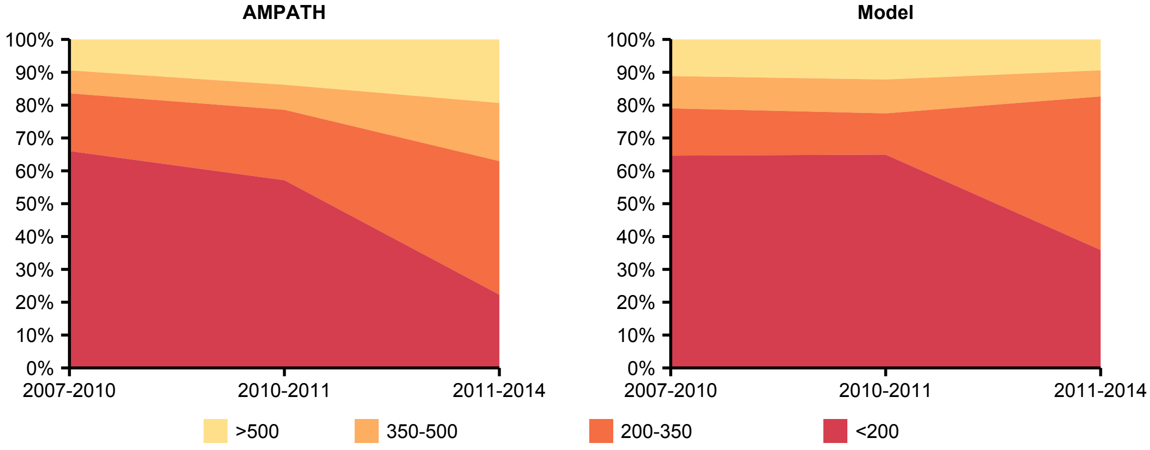

Figure S27. Disease-Led Care Seeking Calibration - CD4 distribution at ART initiation.

(**A**) Among individuals initiating ART between 2007 and 2010.

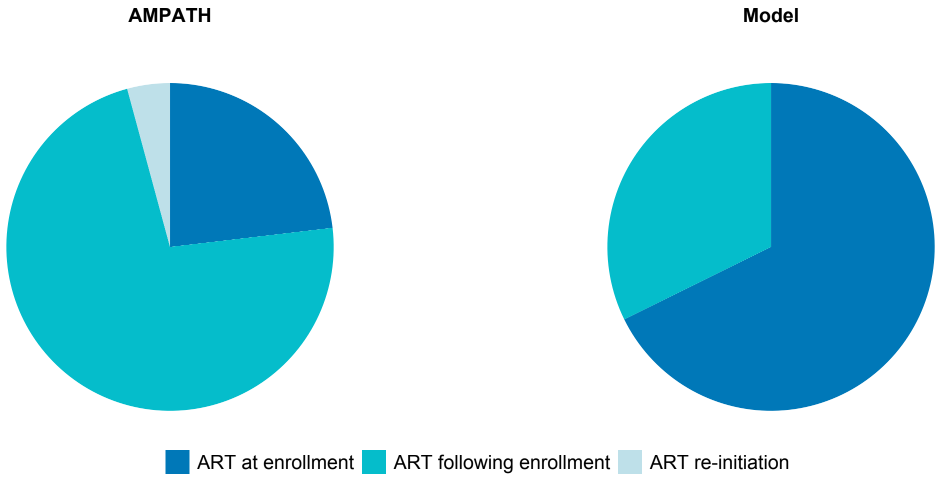

(**B**) Among individuals initiating ART between 2010 and 2011.

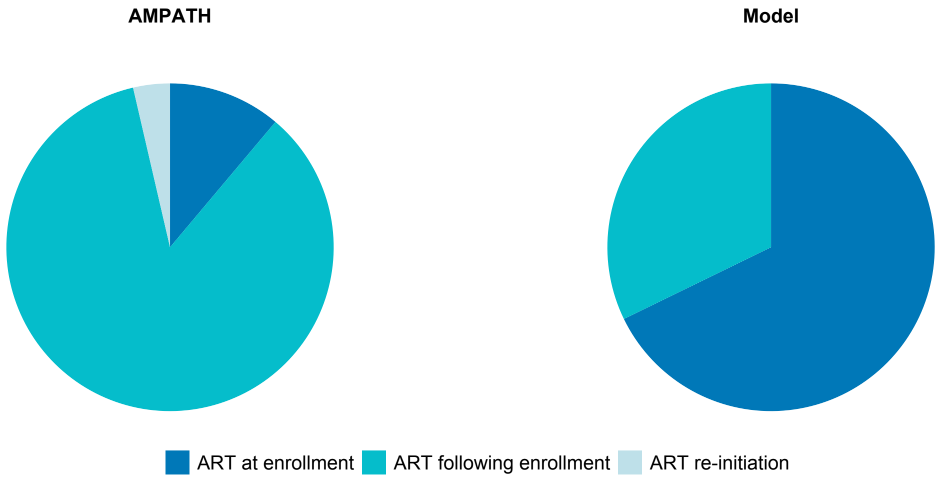

(**C**) Among individuals initiating ART between 2011 and 2014.

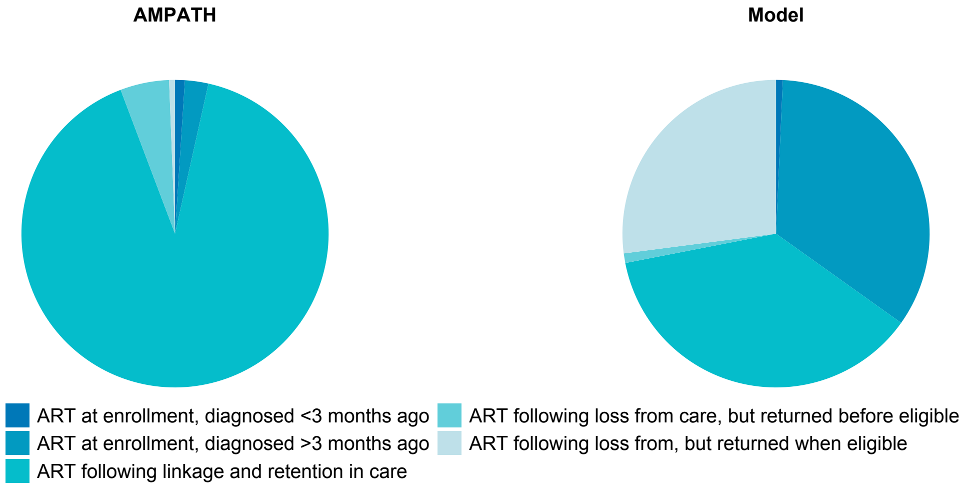

Figure S28. Disease-Led Care Seeking Calibration - Prior care experience among individuals initiating ART.

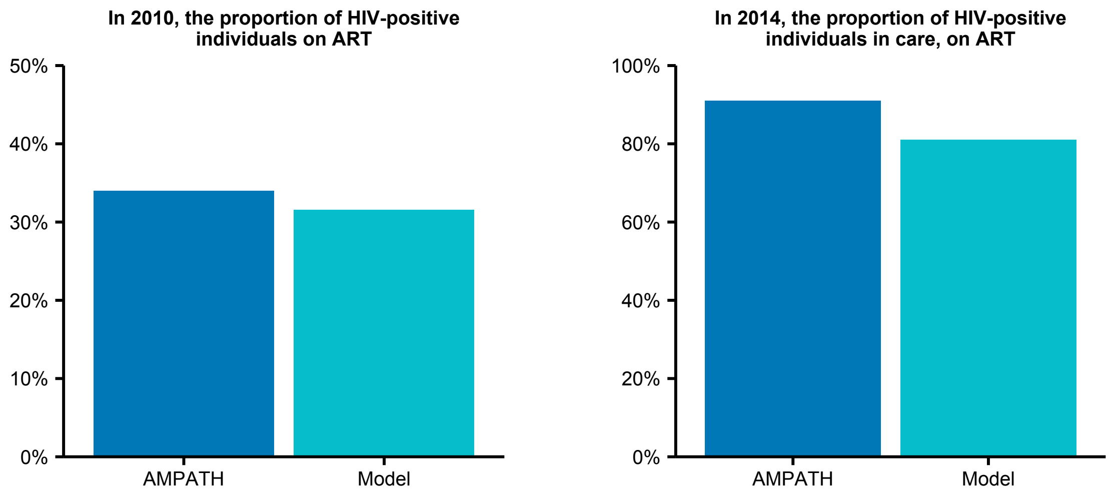

Figure S29. Disease-Led Care Seeking Calibration - Proportion of individuals initiating ART per year.

4.2.8 Disease-Led Care Seeking Calibration: Results to Province-Level Estimates

After calibration to data from AMPATH, we independently compared this "Disease-Led Care Seeking" version of the model to estimates of HIV prevalence from the 2012 Kenya AIDS Indicator Surveys at the province-level, estimates from AMPATH, and to national UNAIDS estimates. With the 'calibration version' of HBCT implemented from 2010 (Section 4.2.2), we compared HIV prevalence in the model to estimates from the 2012 Kenya AIDS Indicator Survey (Fig S30),[38] estimates from AMPATH (Fig S31), and to national estimates of prevalence, AIDS-related death estimates and ART coverage to data from UNAIDS (Fig S32).

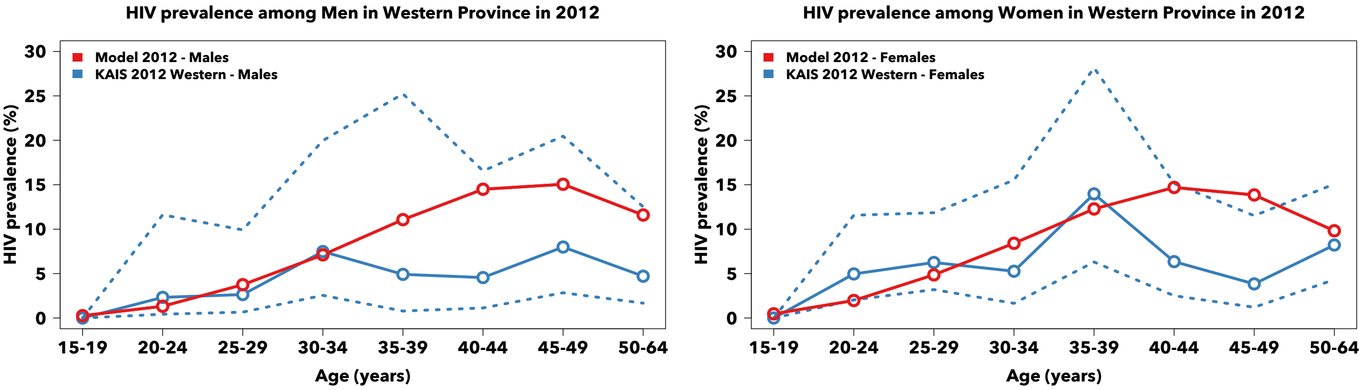

Figure S30. Disease-Led Care Seeking Calibration - Comparison of HIV-prevalence in the model to KAIS 2012 estimates from Western Province.

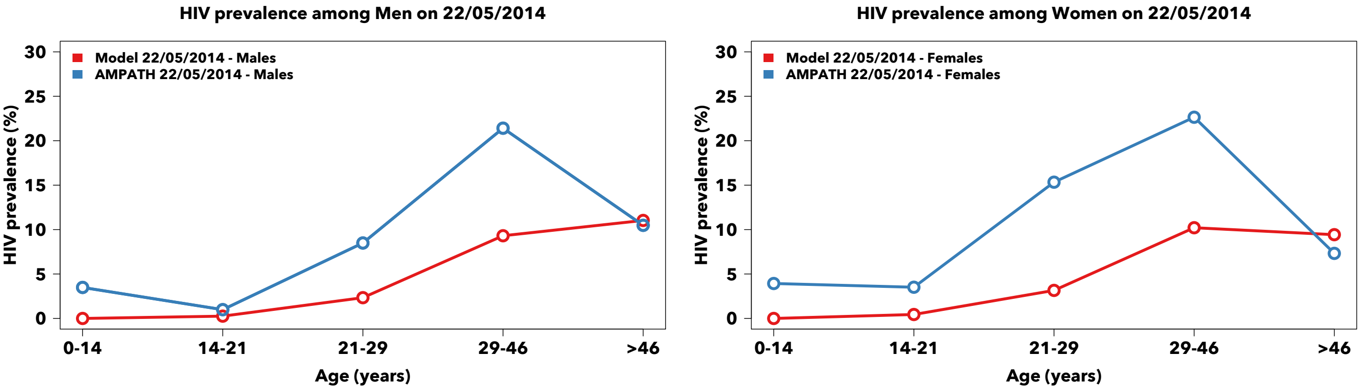

Figure S31. Disease-Led Care Seeking Calibration - Comparison of HIV-prevalence in the model to AMPATH estimates from Bunyala in 2014.

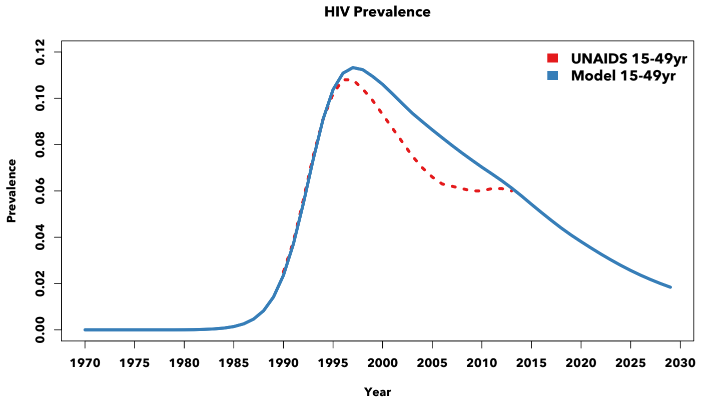

(A) Historical HIV-prevalence trend.

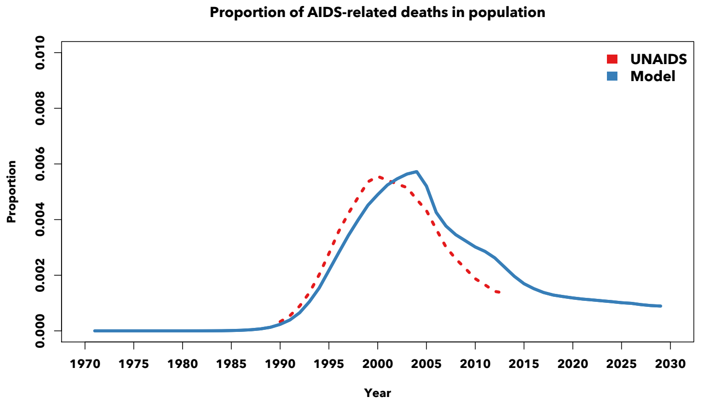

(B) AIDS deaths as a proportion of the total population.

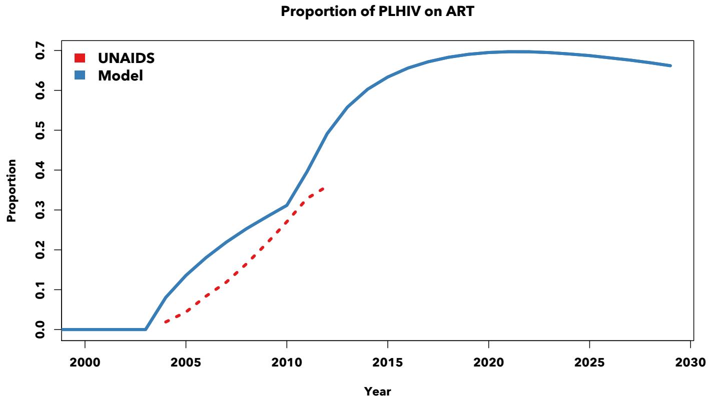

(C) ART coverage.

Figure S32. Disease-Led Care Seeking Calibration - Comparison to UNAIDS estimates from Spectrum.[2]

5 Cost

The cost of care was broken down into individual unit costs. The majority of costs, including the cost of ART care, pre-ART clinic visits and CD4 lab-based tests, were derived from the MATCH Study, a multi-country analysis of 161 treatment facilities across five countries in sub-Saharan Africa.[41,42] The remaining costs were identified in the literature by searching for robust cost estimates from sub-Saharan Africa. Due to the lack of cost data from Kenya, costs were sourced from other countries within sub-Saharan Africa and converted to a US dollar (USD) value. As various sources list component costs from different years, we used the GDP deflator, the ratio of GDP in current local currency to GDP in a constant local currency, to adjust all costs to 2013 USD.

All costs were discounted at 6% per annum from 2010 onwards. All unit costs are shown in table S14 with a flow diagram describing how these costs accumulate over person-time in fig S33.

Table S14. HIV-care cost breakdown.

**Source**	**Item**	**Country**	**Original Year**	**Cost in original year (USD)**	**GDP Deflator in original year**	**GDP Deflator in 2013**	**Difference in GDP Deflator**	**Adjusted Cost**	**Average**
Wright *et al.*[43]	Rapid HIV-test	Zambia	2003	$2.00	178.22	569.53	391.31	**$10**	-
Larson *et al.*[26]	POC CD4 test	South Africa	2010	$23.76	145.08	169.83	24.75	**$30**	-
van Rooyen *et al.*[44]*	HBCT home visit	South Africa	2013	$8.46	169.83	169.83	0	**$8**	-
CHAI MATCH Study[41]	CD4-test (lab)	Ethiopia	2010	$7.11	90.52	160.57	70.06	$12	**$12**
		Malawi	2010	$7.21	126.45	196.92	70.47	$12	
		Rwanda	2010	$6.28	140.21	167.40	27.19	$8	
		Zambia	2010	$6.20	444.35	569.53	125.18	$14	
	Pre-ART Clinic Appointment	Ethiopia	2010	$6.64	90.52	160.57	70.06	$11	**$28**
		Malawi	2010	$6.89	126.45	196.92	70.47	$12	
		Rwanda	2010	$19.61	140.21	167.40	27.19	$25	
		Zambia	2010	$28.56	444.35	569.53	125.18	$64	
	Annual ART Cost	Ethiopia	2010	$158.00	90.52	160.57	70.06	$269	**$367**
		Malawi	2010	$124.00	126.45	196.92	70.47	$211	
		Rwanda	2010	$245.00	140.21	167.40	27.19	$312	
		Zambia	2010	$300.00	444.35	569.53	125.18	$676	

GDP deflator values from World Bank (`http://data.worldbank.org/indicator/NY.GDP.DEFL.ZS`). Cost adjusted for inflation by multiplying cost in original year by one plus the percentage change in GDP deflator. Values in bold are those used in the model. * Secondary analysis of data from van Rooyen *et al.*[44]

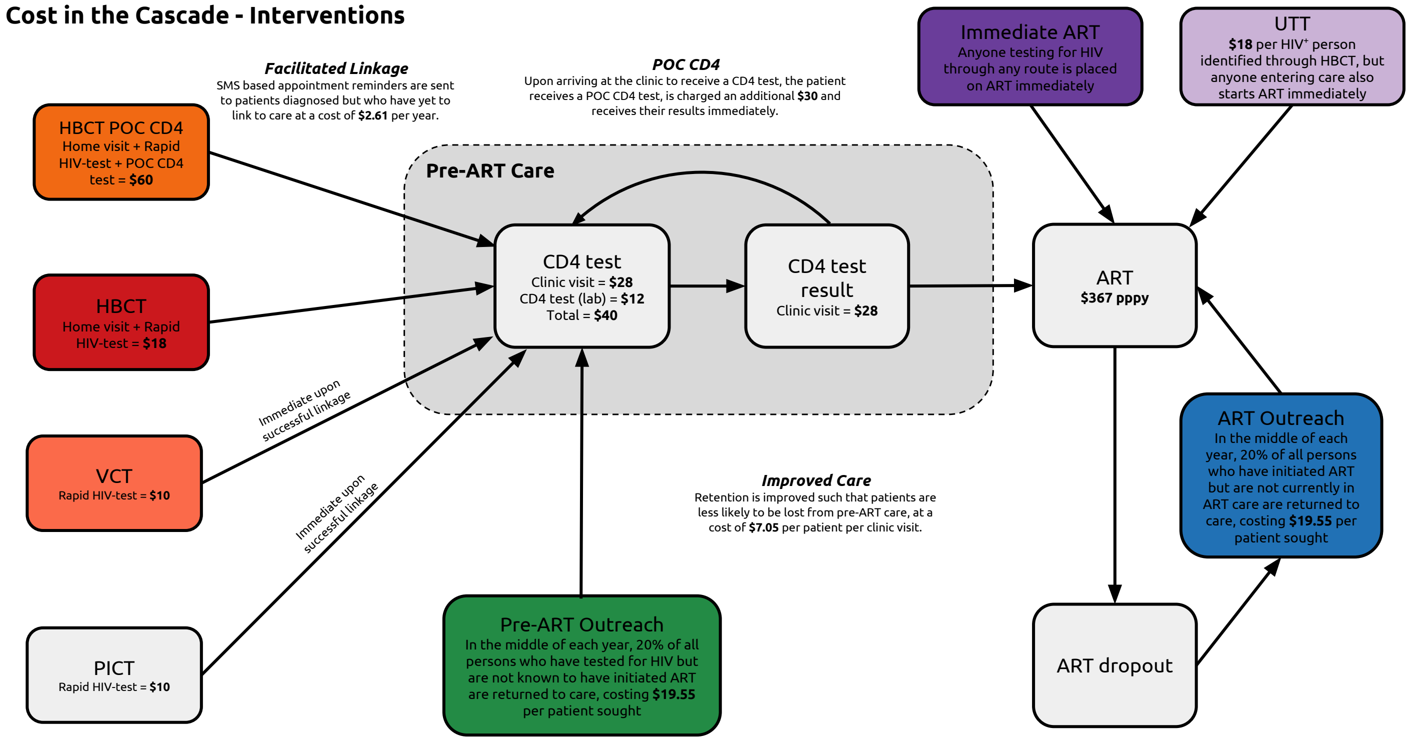

Figure S33. Flow diagram describing the cost function in the model. The cost of the individual components of care accumulate over time and additional costs are incurred when interventions are implemented.

6 DALYs

Patient outcomes are quantified in terms of disability-adjusted life years (DALYs). The impact of individual interventions was assessed by comparing DALYs averted relative to a baseline scenario in the absence of any interventions between 2010 and 2030. The disability weights used in this model were sourced from the Global Burden of Disease Study 2010, comparing life-years lived in different health states to full health, values used are shown in table S15.[45] It is assumed that untreated HIV-infection with a CD4 count of >350 cells/µl carries the same weight as an HIV-positive individual receiving ART. Upon death from HIV, an individual carries a disability weight of one until they reach their natural death date. Disability weights, along with costs, were discounted at 6% per annum from 2010 onwards.

Table S15. Disability weights by health state.

Health State	Disability Weight
HIV-positive, CD4 count >350 cells/µl (untreated)	0.053
HIV-positive, CD4 count 200-350 cells/µl (untreated)	0.221
HIV-positive, CD4 count <200 cells/µl (untreated)	0.547
HIV-positive, on-ART	0.053

7 Interventions

7.1 Intervention Detail

The interventions implemented in the model were identified by first defining specific intervention categories, with each containing interventions targetting a specific aspect of care. We then conducted a review of the literature for relevant interventions to populate each category. We picked one indicative intervention, from each category, on the basis of strong empirical evidence and the explicit quantification of impact and cost (Table 2 & S16). This provides a useful indication of the relative actions of the different categories of intervention, but will not impart the specificity to determine the nature of interventions that would be recommended with that category.

Table S16. Evidence of indicative intervention.

Intervention	Intervention Detail	Assumptions	Reference
**HBCT**	Pilot of home-based counselling and testing intervention in rural Kwazulu-Natal, South Africa. Intervention involved home-based HIV-testing with point-of-care CD4 testing and follow-up visits to improve linkage to care. Pilot intervention achieved 91% coverage and 90% of HIV-infected individuals had linked to care within 3 months.	We ignore predicted linkage values to simulate an HBCT intervention in the absence of POC CD4 testing.	van Rooyen *et al.* (2013)[44]
**Enhanced CT**	Project Accept (HPTN 043) – Trial to assess if HIV-testing could be increased by supplementing clinic-based VCT testing with community-based VCT testing in Tanzania, Zimbabwe and Thailand. 40.2% difference in the proportion of clients receiving HIV-testing through community-based VCT vs. clinic-based VCT.	The intervention involves a 25% increase in baseline HIV-testing.	Sweat *et al.* (2011)[46]
**HBCT (with POC CD4)**	Pilot of home-based counselling and testing intervention in rural Kwazulu-Natal, South Africa. Intervention involved home-based HIV-testing with point-of-care CD4 testing and follow-up visits to improve linkage to care. Pilot intervention achieved 91% coverage and 90% of HIV-infected individuals had linked to care within 3 months.	We assume that linkage is improved by 50%.	van Rooyen *et al.* (2013)[44]
**Facilitated Linkage**	This is based on a trial in Mozambique that explored the impact of a combination of three interventions to improve linkage and retention in pre-ART care in comparison to the standard of care in Mozambique. This combination intervention strategy consisted of providing point-of-care CD4 testing, accelerating ART initiation for eligible individuals and providing SMS based appointment reminders for patients. The trial is still underway and is estimated to be complete by June 2016; however, the study protocal has been published by Elul *et al.* (2014).	We implement a hypothetical SMS based appointment reminder intervention that increases linkage to care by 50% and costs $2.61 per patient per year. No cost data was provided by Elul *et al.* (2014). This cost was derived from Lester *et al.* (2010), which describes a trial assessing the impact of SMS based reminders for ART adherence in Kenya.	Elul *et al.* (2014)[47] & Lester *et al.* (2010)[48]
**VCT POC**	Implementation of point-of-care CD4 testing to assess the impact on loss to follow-up before completion of immunological staging. POC CD4 reduced the proportion of patients lost from care before completing CD4 staging by 36%.	We provide POC CD4 testing to all patients testing through VCT and assume that the risk of not linking to care is eliminated.	Jani *et al.* (2011)[49]
**Pre-ART Outreach**	Pilot intervention utilising a 'patient tracer' to contact patients lost from ART care, ascertain their outcome and if possible assist in returning them to care. Over the four-month intervention, the patient tracer returned 21% of patients lost from care to the clinic.	We assume that a 'patient tracer' is used to assist in returning 20% of patients lost from pre-ART care only.	Rosen *et al.* (2010)[50]
**Improved Care**	Based on a trial of Nurse-led HIV clinics in South Africa by Fairall *et al.* (2012). The trial looked at the impact of task-shifting from doctor-led to nurse-led care through the Streamlining Tasks and Roles to Expand Treatment and Care for HIV (STRETCH) programme on mortality, retention and viral suppression. Amongst HIV-infected patients not on ART, retention in pre-ART care was 10% higher after 12 months when enrolled in nurse-led care compared to doctor-led care. The cost-effectiveness of this intervention was assessed in a follow-up article by Barton *et al.* (2013) in which the per-patient cost of a nurse-led clinic visit was estimated to be $6.49 (2009 USD).	We assume a generic intervention whereby the risk of loss from pre-ART care is reduced by 50%.	Fairall *et al.* (2012)[51] & Barton *et al.* (2013)[52]
**POC CD4**	Implementation of point-of-care CD4 testing to assess the impact on loss to follow-up before completion of immunological staging. POC CD4 reduced the proportion of patients lost from care before completing CD4 staging by 36%.	We provide POC CD4 testing to all patients at the clinic who successfully linked to care. We assume that here, POC CD4 has no impact on linkage but the risk of loss from care between CD4 test and result is eliminated.	Jani *et al.* (2011)[49]
**On-ART Outreach**	Pilot intervention utilising a 'patient tracer' to contact patients lost from ART care, ascertain their outcome and if possible assist in returning them to care. Over the four-month intervention, the patient tracer returned 21% of patients lost from care to the clinic.	We assume that a 'patient tracer' is used to assist in returning 40% of patients lost from ART care only.	Rosen *et al.* (2010)[50]
**Adherence**	m-DOT (modified directly observed therapy) trial in three outpatient clinics in Mombasa, Kenya. Intervention consisted of twice-weekly health centre visits for nurse-observed pill ingestion, adherence support and medication collection. Compared to control group, patients receiving m-DOT were 4.8-fold more adherent to ART.	We assumed that an intervention such as m-DOT was used to improve adherence by 50%.	Sarna *et al.* (2008)[53]
**Immediate ART**	No specific study was used as a basis for this intervention. Using baseline-testing routes (VCT and PICT) we provide ART to all HIV-infected individuals immediately after diagnosis.	We circumnavigate linkage for patients testing through VCT and PICT, and provide ART to patients as soon as they are diagnosed.	-
**Universal Test & Treat**	No specific study was used as a basis for this intervention. In addition to providing immediate ART for all patients, we implement testing outreach in the form on an HBCT intervention.	Patients testing through HBCT must first link to care before receiving ART as we assume that all ART is dispensed at the clinic. VCT and PICT testing are performed next to the clinic and we assume linkage is perfect for patients testing through those routes.	-

7.2 Uniform Care Seeking Calibration: Results

The impact and cost of applying each of the 12 interventions using the "Uniform Care Seeking" calibration is shown in fig S34. Under the assumptions of this calibration that there is a stronger and more uniform health-care seeking behaviour amongst patients (Section 4.2.5), pre-ART interventions generate less impact.

The 'Improved Care' intervention generates 40% less impact when patients seek care more uniformally, as opposed to seeking care faster when symptomatic. This is because of the two main routes into care (VCT and PICT), and in the absence of any other interventions, health-care seeking behaviour drives patients to enter care through PICT, while the rate of entry to care through VCT remains constant. The decision to not allow the rate of seeking care through PICT to be driven by symptoms was due to the characterisation of each care route; whereby PICT was envisaged to be a hospital or health clinic that patients sought when sick. As a result, in this calibration, health-care seeking behaviour is increased to match the number of individuals entering care and initiating ART in the data (Fig S20 & Table S12), thereby increasing the likelihood that any individual will enter care, and reducing the importance of retention in pre-ART care.

While the impact of individual pre-ART interventions decreased, the impact of interventions targeting ART remained roughly constant. This is because the ART outreach and adherence interventions are restricted by the number of patients that ever initiate ART at baseline, and as both model scenarios are calibrated to replicate the same number of individuals to ever initiate ART, the impact of these interventions does not alter significantly. The exception to this is the 'Universal Test & Treat' intervention that generates 39% less impact under this calibration in comparison to a scenario without any interventions implemented. This is because pre-ART retention is increased during calibration and as a result attenuates the impact of this intervention as UTT acts by avoiding pre-ART care and initiating individuals onto treatment immediately.

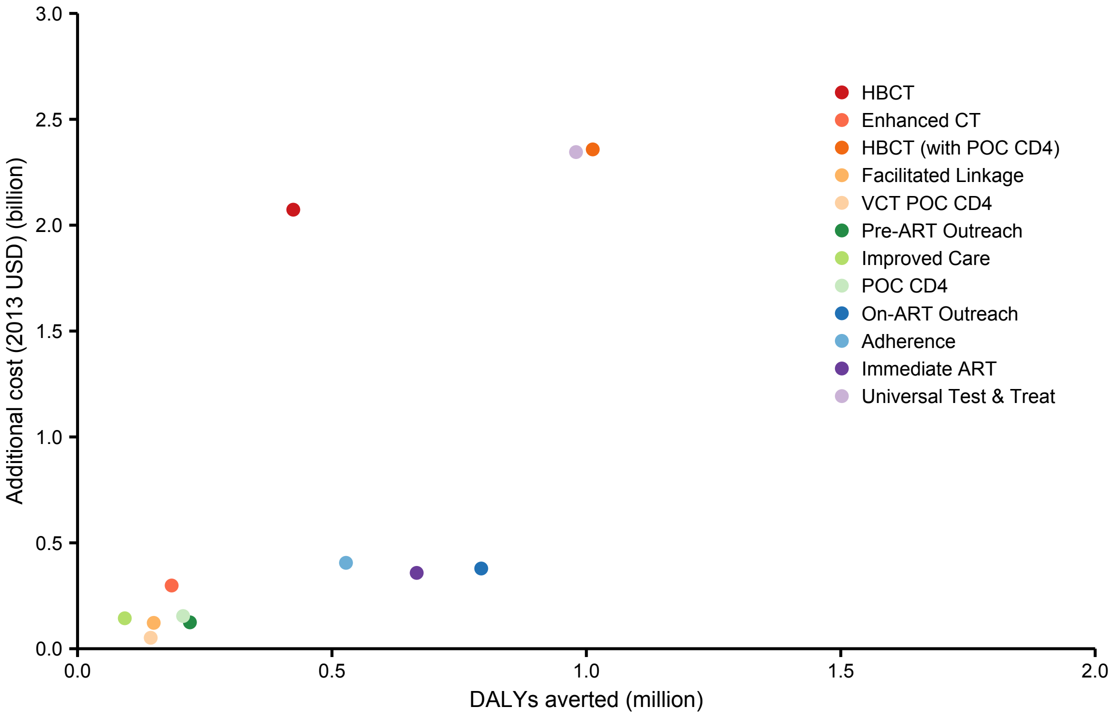

Figure S34. DALYs averted and additional cost of care for individual interventions between 2010 and 2030 when care seeking behaviour is not a function of current health status or previous care experience.

7.3 Disease-Led Care Seeking Calibration: Results

To illustrate the impact of various assumptions regarding the drivers behind health care seeking behaviour, we implemented a second alternate calibration of the model, the "Disease-Led Care Seeking" calibration, in which only the presence of symptoms was able to increase health care seeking behaviour (Section 4.2.7). This is in contrast to the standard calibration (Section 4.2.3), where health care seeking behaviour is a function of symptoms and previous experience of care, and also to the "Uniform Care Seeking" calibration (Section 4.2.5), where care seeking behaviour is stronger and more uniform.

Under the assumptions of this "Disease-Led Care Seeking" calibration, we find that interventions are 17% more impactful and 11% more expensive on average (Fig S35). The 'Pre-ART Outreach' intervention averts over twice as many DALYs as in the standard calibration, but at a cost 43% higher. This is because patients do not seek care faster when lost from pre-ART care; therefore, if a patient is lost from pre-ART care in the absence of any interventions, only the development of symptoms will return them to care faster. This provides the 'Pre-ART Outreach' intervention with a larger pool of individuals with which to have an impact on.

In contrast, under this scenario the 'HBCT' intervention averts 28% fewer DALYs. This is because HBCT diagnoses many infected individuals and links them to pre-ART care, but the impact of this intervention is reduced if patients lost from pre-ART care have a smaller chance of returning. Yet, improving linkage by providing POC CD4 testing to all individuals tested through HBCT will overcome this reduction in impact, as seen by the 'HBCT POC CD4' intervention which averts 9% more DALYs under this calibration.

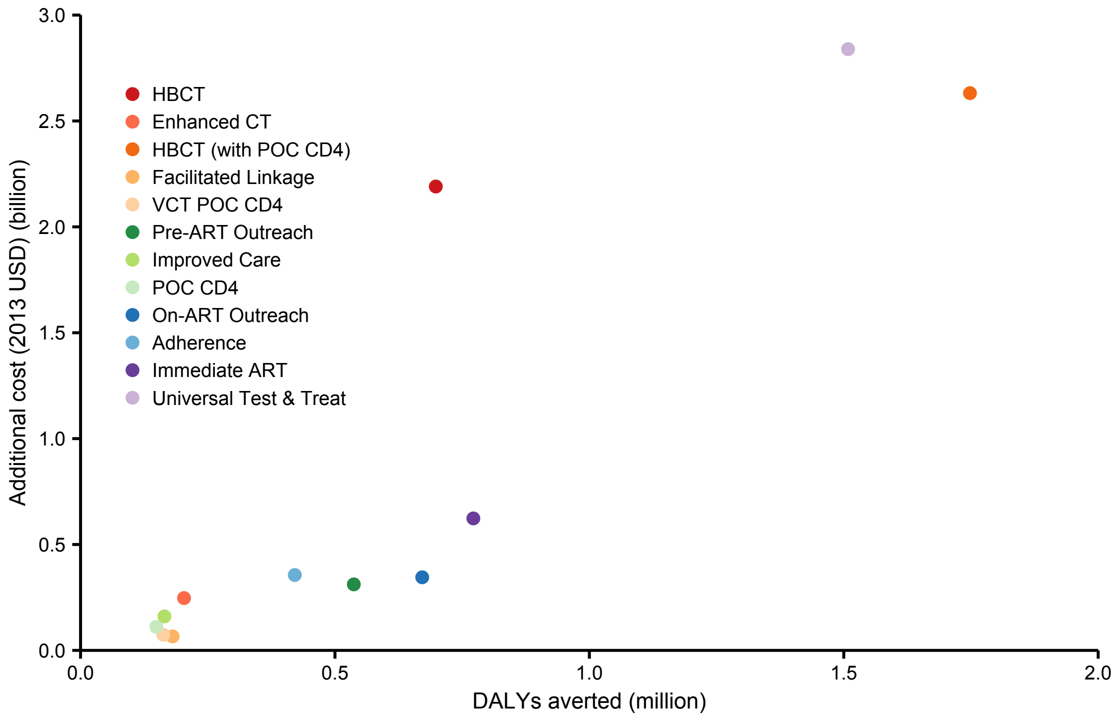

Figure S35. DALYs averted and additional cost of care for individual interventions between 2010 and 2030 when care seeking behaviour is only a function of current health state.

7.4 No Transmission Benefit Scenario: Results

An additional analysis was conducted to investigate the proportion of health benefits that accrue from the indirect impact of interventions reducing the potential for onward transmission of HIV. Interventions impact patient outcomes by directly reducing losses from care, and indirectly through reducing onward transmission. This reduction in transmission is brought about by an intervention increasing the health of infected individuals and reducing their infectiousness, thereby decreasing the force of infection (Section 2).

We simulated an alternate projection in which incidence was not affected by any interventions. Therefore, the impact produced by interventions in this scenario would be entirely from the direct effects of improving patient health. Without transmission benefits, interventions on average, avert 6% fewer DALYs and are 31% more expensive (Fig S36).

The linkage intervention has the largest reduction in impact (33%) compared to the baseline scenario with transmision benefits. While efficiently linking individuals to care and preventing them being lost and potentially initiating ART late, improves population health, a large proportion of this impact comes from indirectly reducing HIV incidence. In contrast, the cost of the 'Immediate ART' intervention increases by 61% without transmission benefits. This is because in the absence of an intervention reducing incidence, a larger number of infected individuals will seek care and initiate ART, increasing the total person-time spent on treatment and thus the overall cost of care.

These results indicate that interventions produce on average 94% of their impact through direct effects on patient outcomes and the remaining 6% from indirectly reducing onward transmission over the period 2010 to 2030. In the event that these indirect effects are overestimated, interventions will still accrue the majority of health benefits from direct effects.

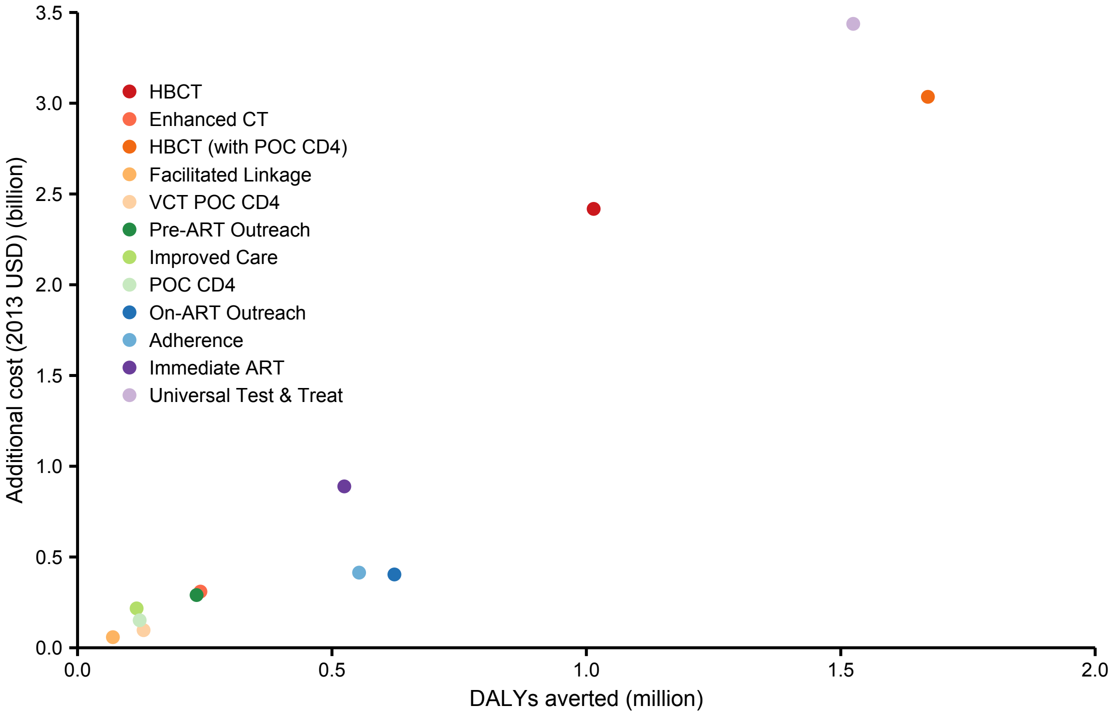

Figure S36. DALYs averted and additional cost of care for individual interventions between 2010 and 2030 where interventions provide no transmission benefits.

7.5 Sources of Mortality

The alternative approaches for strengthening the care cascade generate their impact in different ways (Fig S37). The combination approach does not substantially reduce deaths among those who would not present for HIV-testing (a budget increase of $101M is required for this), but it does reduce deaths among those who have tested by facilitating linkage, improving pre-ART retention and re-engagement, and by reducing deaths among patients in ART care by improving ART outreach (Fig S37, Bar 3). The 'Immediate ART' approach also does not reduce deaths among those who would not naturally present for HIV-testing, but by placing all successfully linked patients onto treatment immediately, avoids the potential for patients disengaging from care and dying before initiating ART (Fig S37, Bar 2). With the 'UTT' approach, there is a substantial reduction in the number of persons that die who were not diagnosed, due to the large outreach component (in the form of HBCT), but impact is moderated by the persisting large number of deaths among those who start ART but subsequently disengage from care (Fig S37, Bar 4).

For comparison, with all interventions operating ('UTT' and combination of cascade interventions), deaths are reduced the most and almost all remaining deaths are among persons on ART, the majority among those who did not start late (Fig S37, Bar 6). This represents the maximum impact of an ART programme in this setting and is a reduction of 43% relative to the baseline scenario, and a reduction of 75% relative to the absence of an ART programme. Further increases are limited by the remaining small excess risk of death for those on ART compared with those uninfected, which may be reduced through new drugs and therapies in the future.

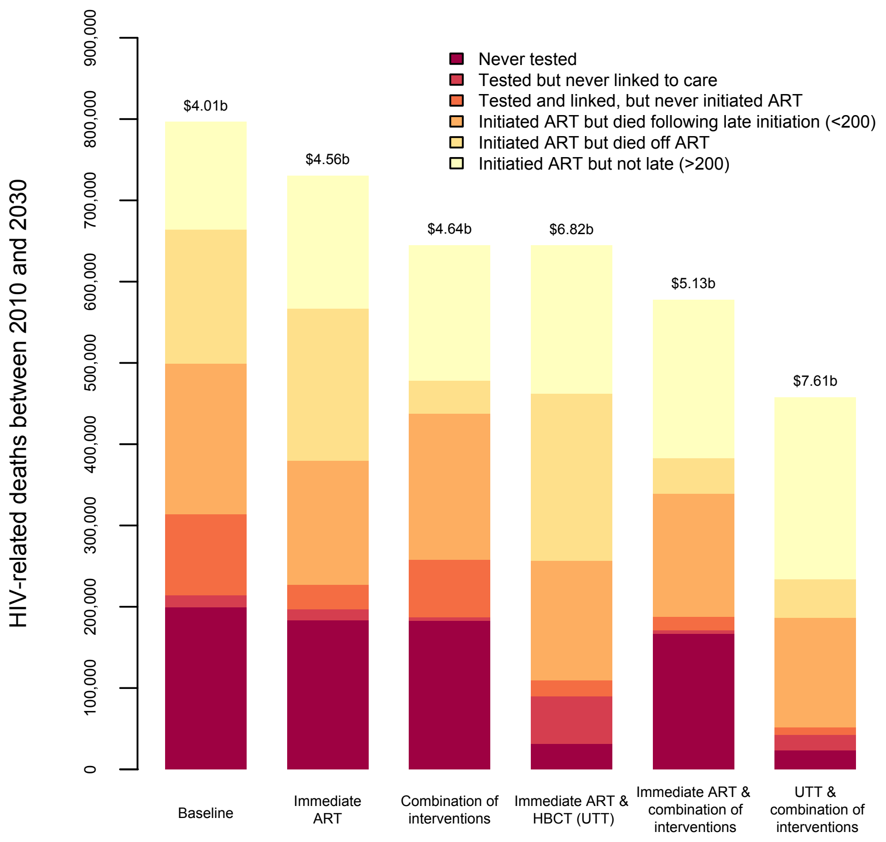

Figure S37. Care experience of deceased individuals who suffered an HIV-related death between 2010 and 2030. Cost at the top of each bar is the total cost of care for all individuals between 2010 and 2030 for each scenario.

8 Sensitivity Analysis

8.1 Methods

Here we analyse the sensitivity of the model to variations in the cost of individual units of care. We are interested in the additional cost of different interventions given differences in underlying costs of care, and what influence this has on the relative cost-effectiveness of each intervention.

To begin, we list all individual units of cost used in the model (Table S17). To analyse the sensitivity of the overall cost of care between 2010 and 2030, to variations in the cost of individual components of care, we first define an upper and lower bound for each unit cost used which we can then sample between. The minimum, or lower bound, is equal to 50% of the 'Initial Cost' (the baseline cost normally used by the model), while the maximum, or upper bound, is equal to 150% of the 'Initial Cost' (Table S17).

Table S17. Costs associated with individual components of care. Row names with an * are intervention specific. All costs are in 2013 USD.

Unit Cost	Initial Cost	Minimum	Maximum
Rapid HIV Test	$10.00	$5.00	$15.00
Pre-ART Clinic Appointment	$28.00	$14.00	$42.00
Lab-based CD4 Test	$12.00	$6.00	$18.00
Annual ART	$367.00	$183.50	$550.50
HBCT Visit*	$8.00	$4.00	$12.00
Linkage*	$2.61	$1.31	$3.92
Improved Care*	$7.05	$3.53	$10.58
POC CD4 Test*	$42.00	$21.00	$63.00
Annual Adherence*	$33.54	$16.77	$50.31
Outreach*	$19.55	$9.78	$29.33

With the upper and lower bounds defined for each unit cost, we have defined the parameter space from which we can sample. We use a Latin Hypercube sampling (LHS) algorithm from the FME package in R to take one thousand random draws from the parameter space.[54]

More specifically, we use LHS to draw one thousand parameter sets by randomly sampling between the upper and lower bounds of each unit cost. For each draw, we calculate the baseline cost (the total cost of care in the absence of any interventions), before calculating the intervention cost (the total cost of care in the presence of the relevant intervention), for each intervention. This is repeated for each draw, until we are left with one thousand results.

We then calculate the additional cost associated with implementing an intervention by taking the intervention cost away from the baseline cost. The cost-effectiveness of each intervention, in terms of the cost per DALY averted, is calculcated by dividing the cost associated with implementing an intervention by the DALYs averted by that intervention. The cost-effectiveness of each intervention was then ranked in order from most to least cost-effective for each draw. The mean ranked position of each intervention, in terms of its relative cost-effectiveness, across all draws was calculated along with the interquartile and absolute ranges.

8.2 Results

Our sensitivity analysis finds that, the cost interval (the difference between the largest and smallest cost for each intervention), increases with the mean cost of the intervention (Fig S38). For example, the most expensive intervention, 'Universal Test & Treat', also has the largest cost interval ($2,714M). The size of the cost interval is directly related to the uptake and unit cost of individual resources by a particular intervention. If more resources, or more expensive resources, are consumed, the cost interval increases.

However, the majority of costs are driven by the unit cost of ART ($367 per year)), which incorporates the cost of ARV's, clinic appointments and monitoring (Table S14). For example, in the absence of any interventions, ART comprises 95% of the total cost of care among HIV-positive individuals. Therefore, interventions prioritising ART, increase person-time spent on ART and hence the proportion of total cost that ART represents. As a result, ART represents 98% of the total cost of care among HIV-positive individuals if 'Universal Test & Treat' is implemented.

If we consider the cost-effectiveness of each intervention in terms of the cost per DALY averted, and order interventions from most to least cost-effective, we find that across all random draws of unit cost (n = 1000), the mean ranked order of interventions is preserved when compared to the cost-effectiveness of interventions in the

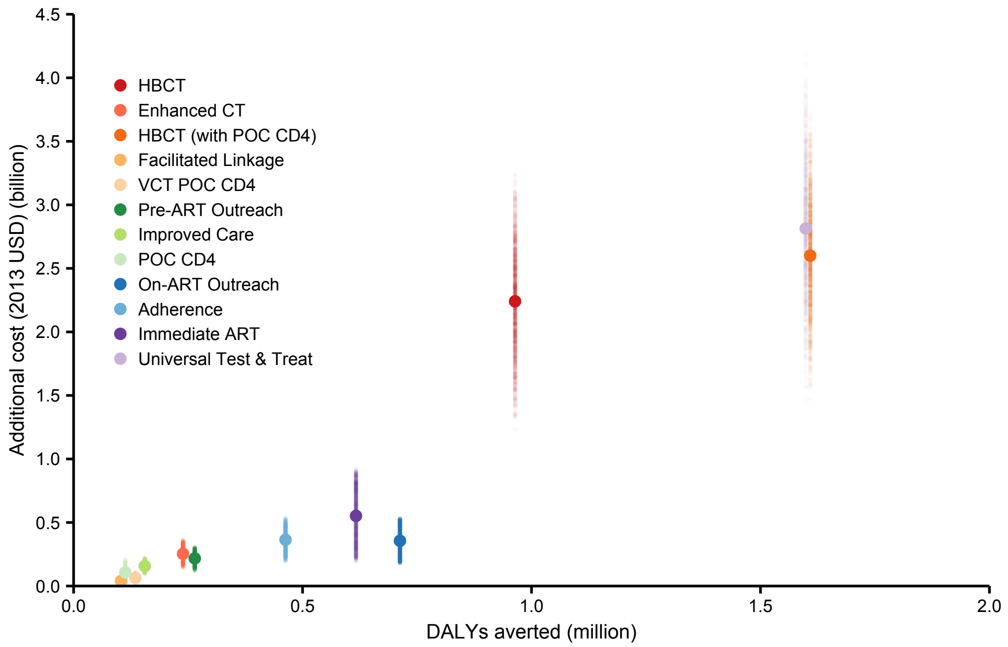

Figure S38. DALYs averted and additional cost of care for interventions acting on the cascade between 2010 and 2030. The sensitivity of each result to variations in the unit cost of each component of care is also illustrated.

main text (Table S18 vs. Table 3). Whilst the rank of the least cost-effective ('HBCT', 'Universal Test & Treat' and 'HBCT (with POC CD4)'), and most cost-effective ('Facilitated Linkage') interventions are highly preserved across all draws, the rank interquartile range (IQR) for interventions in the middle illustrates some variation across draws (Table S18), but the mean rank order is preserved (Fig S39). This analysis indicates that modelled results are not sensitive to fluctuations in unit costs.

Table S18. Ranked table of interventions. Interventions are ranked in order of increasing cost-effectiveness (cost per DALY averted).

Mean Rank	Interventions	Rank IQR	Rank Range
1.22	Facilitated Linkage	1-1	1-4
2.31	VCT POC CD4	2-3	1-4
2.76	On-ART Outreach	2-3	1-5
5.37	Adherence	4-7	1-11
5.45	Pre-ART Outreach	5-6	4-9
6.24	Immediate ART	5-8	3-10
6.59	POC CD4	5-9	1-12
7.47	Improved Care	7-8	4-10
7.67	Enhanced CT	7-9	4-9
9.99	HBCT (with POC CD4)	10-10	8-11
10.93	Universal Test & Treat	11-11	9-11
12.00	HBCT	12-12	11-12

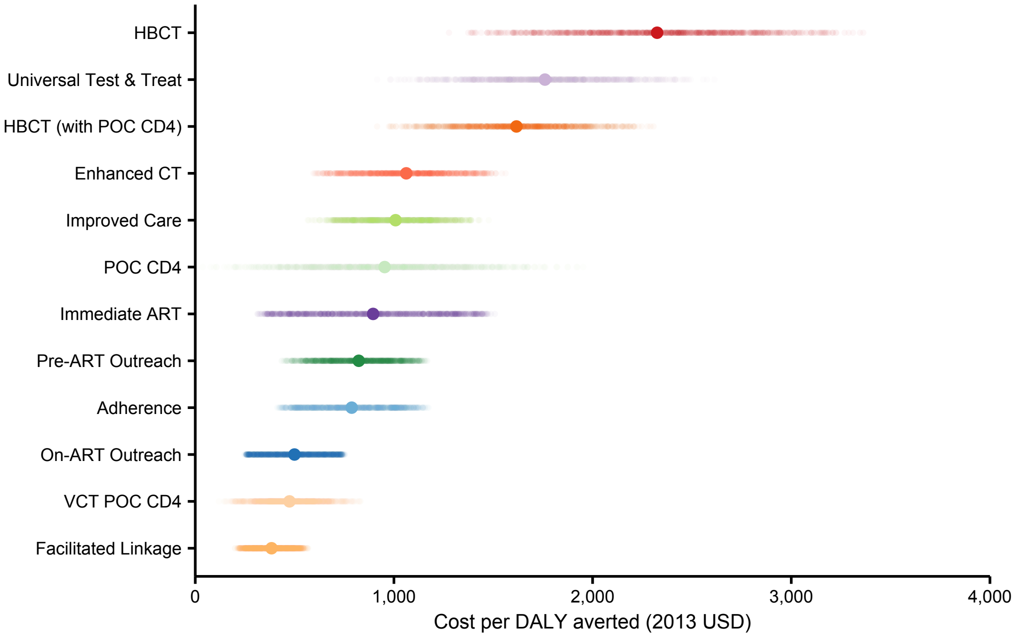

Figure S39. Ranked cost-effectiveness of interventions (cost per DALY averted between 2010 and 2030). One thousand random draws of unit costs for each intervention. The mean cost-effectivenss is denoted by the largest dot.

9 HBCT Analysis

It is important to note several differences between AMPATH HBCT programmes and the HBCT interventions simulated in the model. Firstly, we calibrated our model to a dataset from Bunyala which was closed on the 3rd June, 2014. This dataset detailed the early rounds of HBCT which involved passive linkage of individuals to care following a positive diagnosis.[36] We calibrated our model to these data, and replicated the rollout of HBCT in 2010 with slowly increasing coverage of the population (Section 4.2.2). However, when simulating interventions in the main text, we removed the AMPATH HBCT programme at baseline to allow analysis in the absence of any other interventions. All HBCT interventions implemented thereafter between 2010 and 2030 were hypothetical and not intended to represent the situation at AMPATH.

Secondly, current AMPATH HBCT campaigns now achieve very high coverage of the population and include active follow-up to ensure that patients link to care.[31] As a result, linkage rates are expected to be considerably higher; although, revised estimates of HBCT with passive referral indicate that linkage was potentially underestimated. However, in the absence of an entirely refreshed dataset from Bunyala, we elected to explore an alternative scenario of the HBCT intervention in the model.

The "HBCT" intervention alone links 30% of patients previously unaware of their infection to care, but if patients were aware of their infection, 40% link to care (Table 2). This intervention was intented to simulate HBCT with passive referral to care. In contrast, we simulate "HBCT (with POC CD4)" to illustrate the impact of an HBCT intervention with higher linkage rates afforded by providing POC CD4 to patients immediately alerting them to their current CD4 count. This intervention links 65% of patients previously unaware of their infection to care, and 70% of those aware of infection status (Table 2). This increased linkage and immediate awareness of CD4 count means that patients eligible for treatment at diagnosis will initiate ART faster than patients receiving traditional laboratory-based CD4 tests (Section 4.1). Additionally, there is a further cost of $42 associated with providing a patient with a POC CD4 test (Section 5).

Motivated by the active follow-up to ensure linkage to care developed in recent AMPATH programme efforts,[31] we simulated an additional HBCT intervention with "active referral" in which 90% of patients diagnosed through HBCT are linked to care. This 90% assumption was derived in line with UNAIDS 90-90-90 targets.[55] Analysis of the effectiveness of AMPATH active referral HBCT is currently underway. We find that this increased linkage averts 50% more DALYs between 2010 and 2030 than the "HBCT" intervention with passive referral to care (1.44M vs. 0.96M), at a cost 14% higher ($2,554 vs. $2,241) (Fig S40). However, because patients are not immediately made aware of their CD4 count, this active-referral HBCT intervention averts 10% fewer DALYs than the "HBCT (with POC CD4)" intervention (1.44M vs. 1.61M) (Fig S40), despite the "HBCT (with POC CD4)" intervention linking patients to care at a lower rate (Table 2).

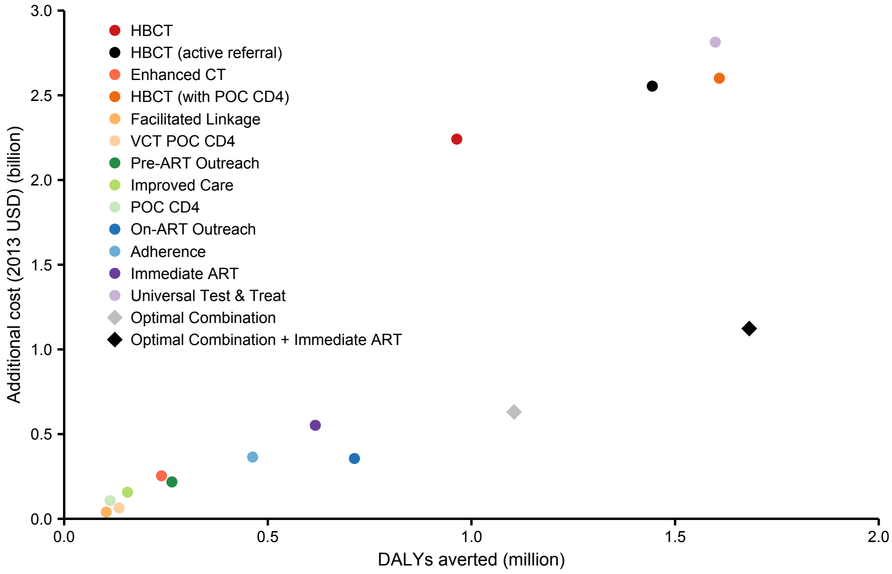

Figure S40. DALYs averted and additional cost of care for individual interventions between 2010 and 2030. Here, we simulate an additional HBCT intervention with active referral, whereby 90% of diagnosed individuals are linked to care.

References

1. United Nations. United Nations World Population Prospects: The 2012 Revision - Mortality;. Available from: http://esa.un.org/unpd/wpp/Excel-Data/mortality.htm.

2. Avenir Health. Spectrum 2013;. Available from: http://www.avenirhealth.org/software-spectrum.php.

3. World Bank. World Development Indicators - Kenya;. Available from: http://data.worldbank.org/country/kenya.

4. Central Intelligence Agency. The World Factbook 2013-2014; 2013. Available from: https://www.cia.gov/library/publications/the-world-factbook/geos/ke.html.

5. United Nations. United Nations World Population Prospects: The 2012 Revision;. Available from: http://esa.un.org/unpd/wpp/Excel-Data/population.htm.

6. World Health Organization. Guidelines for Antiretroviral Drug Therapy in Kenya (2005);. Available from: http://www.who.int/hiv/pub/guidelines/kenya_art.pdf?ua=1.

7. World Health Organization. Antiretroviral Therapy for HIV Infection in Adults and Adolescents (2010);. Available from: http://whqlibdoc.who.int/publications/2010/9789241599764_end.pdf?ua=1.

8. Donnell D, Baeten JM, Kiarie J, Thomas KK, Stevens W, Cohen CR, et al. Heterosexual HIV-1 transmission after initiation of antiretroviral therapy: a prospective cohort analysis. Lancet. 2010;375(9731):2092–2098.

9. World Health Organization. Interim proposal for a WHO staging system for HIV infection and disease. Weekly Epidemiol Record. 1990;65:221–224.

10. Lawn SD, Myer L, Bekker LG, Wood R. CD4 cell count recovery among HIV-infected patients with very advanced immunodeficiency commencing antiretroviral treatment in sub-Saharan Africa. BMC Infect Dis. 2006;6(1):59.

11. Macey R, Oster G. Berkeley Madonna;. Available from: http://www.berkeleymadonna.com/.

12. Todd J, Glynn JR, Marston M, Lutalo T, Biraro S, Mwita W, et al. Time from HIV seroconversion to death: a collaborative analysis of eight studies in six low and middle-income countries before highly active antiretroviral therapy. AIDS. 2007;21:S55–S63.

13. Lodi S, Phillips A, Touloumi G, Geskus R, Meyer L, Thiébaut R, et al. Time from human immunodeficiency virus seroconversion to reaching CD4+ cell count thresholds <200, <350, and <500 cells/mm^3: assessment of need following changes in treatment guidelines. Clin Infect Dis. 2011;53(8):817–825.

14. Schechter MT, Le N, Craib KJ, Le TN, O'Shaughnessy MV, Montaner JS. Use of the Markov model to estimate the waiting times in a modified WHO staging system for HIV infection. J Acquir Immune Defic Syndr. 1995;8(5):474–hyhen.

15. Cascade Collaboration and others. Short-term risk of AIDS according to current CD4 cell count and viral load in antiretroviral drug-naive individuals and those treated in the monotherapy era. AIDS. 2004;18(1):51–58.

16. Badri M, Lawn SD, Wood R. Short-term risk of AIDS or death in people infected with HIV-1 before antiretroviral therapy in South Africa: a longitudinal study. Lancet. 2006;368(9543):1254–1259.

17. Morgan D, Maude GH, Malamba SS, Okongo MJ, Wagner HU, Mulder DW, et al. HIV-1 disease progression and AIDS-defining disorders in rural Uganda. Lancet. 1997;350(9073):245–250.

18. Malamba SS, Morgan D, Clayton T, Mayanja B, Okongo M, Whitworth J. The prognostic value of the World Health Organisation staging system for HIV infection and disease in rural Uganda. AIDS. 1999;13(18):2555–2562.

19. Kagaayi J, Makumbi F, Nakigozi G, Wawer MJ, Gray RH, Serwadda D, et al. WHO HIV clinical staging or CD4 cell counts for antiretroviral therapy eligibility assessment? An evaluation in rural Rakai district, Uganda. AIDS. 2007;21(9):1208–1210.

20. Kassa E, de Wit TFR, Hailu E, Girma M, Messele T, Mariam HG, et al. Evaluation of the World Health Organization staging system for HIV infection and disease in Ethiopia: association between clinical stages and laboratory markers. AIDS. 1999;13(3):381–389.

21. Jaffar S, Birungi J, Grosskurth H, Amuron B, Namara G, Nabiryo C, et al. Use of WHO clinical stage for assessing patient eligibility to antiretroviral therapy in a routine health service setting in Jinja, Uganda. AIDS Res Ther. 2008;5(4).

22. Baveewo S, Ssali F, Karamagi C, Kalyango JN, Hahn JA, Ekoru K, et al. Validation of World Health Organisation HIV/AIDS clinical staging in predicting initiation of antiretroviral therapy and clinical predictors of low CD4 cell count in Uganda. PLoS ONE. 2011;6(5):e19089.

23. May M, Boulle A, Phiri S, Messou E, Myer L, Wood R, et al. Prognosis of patients with HIV-1 infection starting antiretroviral therapy in sub-Saharan Africa: a collaborative analysis of scale-up programmes. Lancet. 2010;376(9739):449–457.

24. Johnson LF, Mossong J, Dorrington RE, Schomaker M, Hoffmann CJ, Keiser O, et al. Life expectancies of South African adults starting antiretroviral treatment: collaborative analysis of cohort studies. PLoS Med. 2013;10(4):e1001418.

25. Lawn SD, Little F, Bekker LG, Kaplan R, Campbel E, Orrell C, et al. Changing mortality risk associated with CD4 cell response to antiretroviral therapy in South Africa. AIDS. 2009;23(3):335.

26. Larson B, Schnippel K, Ndibongo B, Long L, Fox MP, Rosen S. How to estimate the cost of point-of-care CD4 testing in program settings: an example using the Alere Pima Analyzer in South Africa. PLoS ONE. 2012;7(4):e35444.

27. Rachlis B, Ochieng D, Geng E, Rotich E, Ochieng V, Maritim B, et al. Evaluating outcomes of patients lost to follow-up in a large comprehensive care treatment program in western Kenya. J Acquir Immune Defic Syndr. 2015;68(4):e46–e55.

28. Tierney WM, Rotich JK, Hannan TJ, Siika AM, Biondich PG, Mamlin BW, et al. The AMPATH medical record system: creating, implementing, and sustaining an electronic medical record system to support HIV/AIDS care in western Kenya. Stud Health Technol Inform. 2007;129(1):372.

29. Braitstein P, Siika A, Hogan J, Kosgei R, Sang E, Sidle J, et al. A clinician-nurse model to reduce early mortality and increase clinic retention among high-risk HIV-infected patients initiating combination antiretroviral treatment. J Int AIDS Soc. 2012;15(1):7.

30. Wachira J, Ndege S, Koech J, Vreeman RC, Ayuo P, Braitstein P. HIV testing uptake and prevalence among adolescents and adults in a large home-based HIV testing program in western Kenya. J Acquir Immune Defic Syndr. 2014;65(2):e58–e66.

31. AMPATH. Case Study: Identifying best practices for HIV service delivery. AMPATH; 2014. Available from: `http://www.ampathkenya.org/media/78349/final_ampath_case_study.pdf`.

32. Kimaiyo S, Were M, Shen C, Ndege S, Braitstein P, Sidle J, et al. Home-based HIV counselling and testing in western Kenya. East Afr Med J. 2010;87(3):100–108.

33. Tierney WM, Achieng M, Baker E, Bell A, Biondich P, Braitstein P, et al. Experience implementing electronic health records in three East African countries. Stud Health Technol Inform. 2010;160(Pt 1):371–5.

34. Einterz RM, Kimaiyo S, Mengech HN, Khwa-Otsyula BO, Esamai F, Quigley F, et al. Responding to the HIV pandemic: the power of an academic medical partnership. Acad Med. 2007;82(8):812–818.

35. Boender TS, Sigaloff KC, McMahon JH, Kiertiburanakul S, Jordan MR, Barcarolo J, et al. Long-term virological outcomes of first-line antiretroviral therapy for HIV-1 in low-and middle-income countries: a systematic review and meta-analysis. Clin Infect Dis. 2015;p. civ556.

36. Genberg BL, Naanyu V, Wachira J, Hogan JW, Sang E, Nyambura M, et al. Linkage to and engagement in HIV care in western Kenya: an observational study using population-based estimates from home-based counselling and testing. Lancet HIV. 2015;2(1):e20–e26.

37. Hallett TB, Eaton JW. A side door into care cascade for HIV-infected patients? J Acquir Immune Defic Syndr. 2013;63:S228–S232.

38. KAIS. Kenya AIDS Indicator Survey - 2012. KAIS; 2014. Available from: `http://www.nacc.or.ke/images/documents/KAIS-2012.pdf`.

39. Camlin CS, Kwena ZA, Dworkin SL. Jaboya vs. jakambi: Status, negotiation, and HIV risks among female migrants in the "sex for fish" economy in Nyanza Province, Kenya. AIDS Educ Prev. 2013;25(3):216.

40. Eaton JW, Rehle TM, Jooste S, Nkambule R, Kim AA, Mahy M, et al. Recent HIV prevalence trends among pregnant women and all women in sub-Saharan Africa: implications for HIV estimates. AIDS. 2014;28:S507–S514.

41. Tagar E, Sundaram M, Condliffe K, Over M, Matatityo B, Chimbwandira F, et al.. Multi-Country Analysis of Treatment Costs for HIV/AIDS (MATCH): Unit Costing at 161 Representative Facilities in Ethiopia, Malawi, Rwanda, South Africa and Zambia. Harvard Dataverse;. Available from: `http://hdl.handle.net/1902.1/18843`.

42. Tagar E, Sundaram M, Condliffe K, Matatiyo B, Chimbwandira F, Chilima B, et al. Multi-Country Analysis of Treatment Costs for HIV/AIDS (MATCH): Facility-Level ART Unit Cost Analysis in Ethiopia, Malawi, Rwanda, South Africa and Zambia. PLoS ONE. 2014 11;9(11):e108304. Available from: `http://dx.doi.org/10.1371%2Fjournal.pone.0108304`.

43. Wright RJ, Stringer JS. Rapid testing strategies for HIV-1 serodiagnosis in high-prevalence African settings. Am J Prev Med. 2004;27(1):42–48.

44. van Rooyen H, Barnabas RV, Baeten JM, Phakathi Z, Joseph P, Krows M, et al. High HIV testing uptake and linkage to care in a novel program of home_based HIV counseling and testing with facilitated referral in KwaZulu-Natal, South Africa. J Acquir Immune Defic Syndr. 2013;64(1):e1.

45. Salomon JA, Vos T, Hogan DR, Gagnon M, Naghavi M, Mokdad A, et al. Common values in assessing health outcomes from disease and injury: disability weights measurement study for the Global Burden of Disease Study 2010. Lancet. 2013;380(9859):2129–2143.

46. Sweat M, Morin S, Celentano D, Mulawa M, Singh B, Mbwambo J, et al. Community-based intervention to increase HIV testing and case detection in people aged 16–32 years in Tanzania, Zimbabwe, and Thailand (NIMH Project Accept, HPTN 043): a randomised study. Lancet Infect Dis. 2011;11(7):525–532.

47. Elul B, Lahuerta M, Abacassamo F, Lamb MR, Ahoua L, McNairy ML, et al. A combination strategy for enhancing linkage to and retention in HIV care among adults newly diagnosed with HIV in Mozambique: study protocol for a site-randomized implementation science study. BMC Infect Dis. 2014;14(1):549.

48. Lester RT, Ritvo P, Mills EJ, Kariri A, Karanja S, Chung MH, et al. Effects of a mobile phone short message service on antiretroviral treatment adherence in Kenya (WelTel Kenya1): a randomised trial. Lancet. 2010;376(9755):1838–1845.

49. Jani IV, Sitoe NE, Alfai ER, Chongo PL, Quevedo JI, Rocha BM, et al. Effect of point-of-care CD4 cell count tests on retention of patients and rates of antiretroviral therapy initiation in primary health clinics: an observational cohort study. Lancet. 2011;378(9802):1572–1579.

50. Rosen S, Ketlhapile M. Cost of using a patient tracer to reduce loss to follow-up and ascertain patient status in a large antiretroviral therapy program in Johannesburg, South Africa. Trop Med Int Health. 2010;15(s1):98–104.

51. Fairall L, Bachmann MO, Lombard C, Timmerman V, Uebel K, Zwarenstein M, et al. Task shifting of antiretroviral treatment from doctors to primary-care nurses in South Africa (STRETCH): a pragmatic, parallel, cluster-randomised trial. Lancet. 2012;380(9845):889–898.

52. Barton GR, Fairall L, Bachmann MO, Uebel K, Timmerman V, Lombard C, et al. Cost-effectiveness of nurse-led versus doctor-led antiretroviral treatment in South Africa: pragmatic cluster randomised trial. Trop Med Int Health. 2013;18(6):769–777.

53. Sarna A, Luchters S, Geibel S, Chersich MF, Munyao P, Kaai S, et al. Short-and long-term efficacy of modified directly observed antiretroviral treatment in Mombasa, Kenya: a randomized trial. J Acquir Immune Defic Syndr. 2008;48(5):611–619.

54. Soetaert K, Petzoldt T. Inverse Modelling, Sensitivity and Monte Carlo Analysis in R Using Package FME. J Stat Softw. 2010;33(3):1–28. Available from: http://www.jstatsoft.org/v33/i03/.

55. UNAIDS. 90-90-90 - An ambitious treatment target to help end the AIDS epidemic. UNAIDS; 2014. Available from: http://www.unaids.org/en/resources/documents/2014/90-90-90.
